# Exploring the impact of analysis software on task fMRI results

**DOI:** 10.1002/hbm.24603

**Published:** 2019-05-02

**Authors:** Alexander Bowring, Camille Maumet, Thomas E. Nichols

**Affiliations:** ^1^ Big Data Institute, Li Ka Shing Centre for Health Information and Discovery, Nuffield Department of Population Health University of Oxford Oxford UK; ^2^ Inria, Univ Rennes, CNRS Inserm, IRISA UMR 6074, Empenn ERL U 1228 Rennes France; ^3^ Wellcome Centre for Integrative Neuroimaging, FMRIB, Nuffield Department of Clinical Neurosciences University of Oxford Oxford UK; ^4^ Department of Statistics University of Warwick Coventry UK

**Keywords:** AFNI, analytic flexibility, analytic variability, fMRI, FSL, reproducibility, software comparison, SPM, task‐fMRI

## Abstract

A wealth of analysis tools are available to fMRI researchers in order to extract patterns of task variation and, ultimately, understand cognitive function. However, this “methodological plurality” comes with a drawback. While conceptually similar, two different analysis pipelines applied on the same dataset may not produce the same scientific results. Differences in methods, implementations across software, and even operating systems or software versions all contribute to this variability. Consequently, attention in the field has recently been directed to reproducibility and data sharing. In this work, our goal is to understand how choice of software package impacts on analysis results. We use publicly shared data from three published task fMRI neuroimaging studies, reanalyzing each study using the three main neuroimaging software packages, AFNI, FSL, and SPM, using parametric and nonparametric inference. We obtain all information on how to process, analyse, and model each dataset from the publications. We make quantitative and qualitative comparisons between our replications to gauge the scale of variability in our results and assess the fundamental differences between each software package. Qualitatively we find similarities between packages, backed up by Neurosynth association analyses that correlate similar words and phrases to all three software package's unthresholded results for each of the studies we reanalyse. However, we also discover marked differences, such as Dice similarity coefficients ranging from 0.000 to 0.684 in comparisons of thresholded statistic maps between software. We discuss the challenges involved in trying to reanalyse the published studies, and highlight our efforts to make this research reproducible.

## 1 INTRODUCTION

Functional magnetic resonance imaging (fMRI) for human brain mapping gives researchers remarkable power to probe the underpinnings of human cognition, behaviour, and emotion. As an active field of research for over 25 years, there are now a multitude of ways to analyse a single neuroimaging study. The plethora of techniques and tools available are a platform from which we have the potential to gain remarkable insight into how the human brain works. However, high analytic flexibility has also been pinpointed as a key factor that can lead to false‐positive and nonreproducible results (Hong et al., 2019; Ioannidis, 2005; Wager et al., 2009). Because of this, neuroimagers must be particularly judicious: choice of analysis pipeline, operating system, and even software version may influence the final research outcome of a study.

The extent to which varying processing conditions can lead to discrepancies in observed results has been highlighted throughout the neuroimaging literature. In a study examining the use of FreeSurfer to measure the cortical thickness and volume of structural brain images (Gronenschild et al., 2012), a change in software version was shown to lead to increases of over 10% in observed anatomical measurement; a switch in workstation from which the software was run also manifested significant deviations in the final result. In related work (Glatard et al., 2015), changes in operating system lead to differences in the results of an independent component analysis of resting state fMRI data carried out using FSL. Here, disparities in both the number of components determined as well as information between matched components were found when the analysis was conducted on two separate computing clusters. For task‐based fMRI, the impact of methodological choices has been investigated extensively. Choices for each individual procedure in the analysis pipeline (e.g., head‐motion regression (Lund et al., 2005), temporal filtering (Skudlarski et al., 1999), and autocorrelation correction (Woolrich et al., 2001)) alongside the order in which these procedures are conducted (Carp, 2013) can deeply influence the final determined areas of brain activation. In perhaps the most comprehensive of such studies (Carp, 2012a), a single publicly available fMRI dataset was analysed using over 6,000 unique analysis pipelines, generating 34,560 unique thresholded activation images. These results displayed a substantial degree of flexibility in both the sizes and locations of significant activation. In combination, these examples of research shape a sombre picture for the possibility of study reproducibility.

While each of the aforementioned studies investigated the effect of either software version, operating system, or analysis pipeline on analytic variability, the choice of software package for carrying out the analysis remained fixed in each study. This is despite a vast array of analysis packages that are now freely available to researchers. The three most popular of these packages for fMRI data analysis are AFNI (RRID:SCR_005927; [Cox, 1996]), FSL (RRID:SCR_002823; [Jenkinson et al., 2012]), and SPM (RRID:SCR_007037; [Penny et al., 2011]). While SPM is the oldest, FSL has grown in popularity and together the three packages have been estimated to account for 80% of published functional neuroimaging results (Carp, 2012b). Although there are differences in how each software package models and processes data, the analysis framework for task fMRI—now a mature research area—is expected to be similar across software, and hence the results yielded from each package should be comparable. We therefore seek to answer the question: How much of the variability in neuroimaging results is attributable to the choice of analysis software package?

In this work we reanalyse data from three published neuroimaging studies using each of the three main software packages and quantify differences in the results. We choose three publications with data that have been made publicly available on the OpenfMRI database (RRID:SCR_005031, http://openfmri.org; [Poldrack et al., 2013]), recently relaunched as OpenNeuro (http://openneuro.org), and attempt to recreate the main figure from each publication by replicating the original analysis within each package. These particular studies were selected on the basis that they reported clearly defined regions of brain activation and utilised analysis procedures feasible across the three software packages. We then make a number of comparisons to assess the similarity of our results. While a similar study from our group explored the results produced by each of these packages after implementing analysis pipelines using the default settings in each software (Pauli et al., 2016), here we attempt to make the analysis pipelines as similar as possible while still maintaining comparability across the three packages. While our primary focus is comparing standard results across software, we also aim to address recent concerns about the multiple‐testing‐corrected parametric inferences that each of these studies used (Eklund et al., 2016). For each study, we also conduct equivalent inference procedures (when possible) using nonparametric statistics in each package.

Although our work has been primarily designed to understand the differences between software packages, we also see this as an exercise in computational reproducibility (Peng, 2011). In recent years, a number of initiatives and guidelines (Poldrack et al., 2017) have materialised to ensure research is conducted in an open and transparent fashion. For each of our analyses, we confine ourselves to the respective publication for all information on how to process and model the data. We discuss the challenges involved in this process, and evaluate whether our reanalyses are a success by comparing our results to those given in the main figure of the respective publication. Great care has also been taken to ensure all figures and results presented here are themselves reproducible; we describe the scripts, notebooks, and other tools used to make this possible which we believe are highly generalizable across neuroimaging studies.

## 2 METHODS

### 2.1 Study description and data source

We selected three functional fMRI studies for reanalysis from the publicly accessible OpenfMRI data repository: ds000001 (Revision: 2.0.4; [Schonberg et al., 2012]), ds000109 (Revision 2.0.2; [Moran et al., 2012]), and ds000120 (Revision 1.0.0; [Padmanabhan et al., 2011]). Each of the datasets have been organised in compliance with the Brain Imaging Data Structure (BIDS, RRID:SCR_016124; [Gorgolewski et al., 2016]). These datasets were chosen following an extensive selection procedure (carried out between May 2016 and November 2016), whereby we vetted the associated publication for each dataset stored in the repository. We sought studies with simple analysis pipelines and clearly reported regions of brain activation that would be easily comparable to our own results. Exclusion criteria included the use of custom software, activations defined using small volume correction, and application of more intricate methods such as region of interest and robust regression analysis, which we believed could be impractical to implement across all analysis software. A full description of the paradigm for each of our chosen studies is included in the respective publication; here we give a brief overview.

For the ds000001 study, 16 healthy adult subjects participated in a balloon analogue risk task over three scanning sessions. On each trial, subjects were presented with a simulated balloon, and offered a monetary reward to “pump” the balloon. With each successive pump the money would accumulate, and at each stage of the trial subjects had a choice of whether they wished to pump again or cash‐out. After a certain number of pumps, which varied between trials, the balloon exploded. If subjects had cashed‐out before this point they were rewarded with all the money they had earned during the trial; however, if the balloon exploded all money accumulated was lost. Three different coloured “reward” balloons were used between trials, each having a different explosion probability, as well as a grey “control” balloon, which had no monetary value and would disappear from the screen after a predetermined number of pumps. Here we reproduce the result contrasting the parametrically modulated activations of pumps of the reward balloons versus pumps of the control balloon, corresponding to Figure 3 and Table 2 in the original article.

The ds000109 study investigated the ability of people from different age‐groups to understand the mental state of others. A total of 48 subjects were scanned, although 43 had acceptable data for the false belief task—29 younger adults and 14 older adults. In this task participants listened to either a “false belief” or “false photo” story. A false belief story would entail an object being moved from one place to another, with certain characters witnessing the change in location while others were unaware. False photo stories were similar except involved some physical representation, such as a photo of an object in a location from which it had been subsequently removed. The task had a block design where stories were represented for 10 s, after which participants had to answer a question about one of the character's perceptions of the location of the object. We reproduce the contrast map of false belief versus false photo activations for the young adults, corresponding to Figure 5a and Table 3 from the original publication.

Finally, the the ds000120 study explored reward processing across different age groups. fMRI results are reported on 30 subjects, with 10 participants belonging to each of the three age groups (children, adolescents, and adults). Participants took part in an antisaccade task where a visual stimuli was presented in each trial and subjects were instructed to quickly fixate their gaze on the side of the screen opposite to the stimuli. Prior to a trial, subjects were given a visual cue to signal whether or not they had the potential to win a monetary reward based on their upcoming performance (a “reward” or “neutral” trial). In this article we reproduce the main effect of time activation map—an *F*‐statistic for any nonzero coefficients in the sine HRF basis—corresponding to Figure 3 and Table 1 in the original publication.

**Table 1:** Software processing steps

	Processing step	AFNI	FSL	SPM
Preprocessing	Script	@SSWarper^a^ afni_proc.py	FEAT First‐level analysis	Batch (*multiple modules*)
	Slice‐timing^b^	‐tshift_opts_ts ‐tpattern	Prestats: Slice timing correction	Slice timing
	Realignment/motion correction	‐volreg_align_e2a	Prestats: Motion correction: MCFLIRT	Realign: estimate and reslice
	Segmentation	*Not applied*	*Not applied*	Segment
	Brain extraction (anatomical)	‐copy_anat [@SSWarper result] ‐anat_has_skull no	bet (*command line*)	Image calculator^c^
	Brain extraction (functional)	*Not applied*	Prestats: BET brain extraction	*Not applied*
	Intrasubject Coregistration	‐align_opts_aea ‐giant_move ‐check_flip	Registration: Normal search, BBR	Coregister: Estimate
	Intersubject registration	‐tlrc_base ‐volreg_tlrc_warp ‐tlrc_NL_warp ‐tlrc_NL_warped_dsets [@SSWarper result]	Registration: nonlinear, warp resolution 10 mm	Normalise: Write
	Analysis voxel size	‐volreg_warp_dxyz (*overriding default determined from functional images*)	*Determined by anatomical template voxel sizes*.	Normalise: Write: Writing options: Voxel sizes
	Smoothing	‐blur_size	Prestats: Spatial smoothing FWHM (mm)	Smooth
First‐level	Script	afni_proc.Py	FEAT First‐level analysis	Specify first‐level
	Model specification	‐regress_stim_times ‐regress_stim_labels ‐regress_basis_multi ‐regress_stim_types	Stats: Full model setup:EVs	fMRI model specification
	Inclusion of 6 motion parameters	*Implicitly added within ‘regress’ block*	Stats: Standard motion parameters	fMRI model specification: Data & Design: Multiple regressors: Realignment Param file
	Model estimation	*Nothing to specify*	*Nothing to specify*	Model estimation
	Contrasts	‐regress_3dD_stop ‐regress_reml_exec ‐regress_opts_3dD ‐gltsym	Stats: Full model setup: contrasts	Contrast manager
Second‐level	Script	3dMEMA 3dMVM^b^	FEAT Higher‐level analysis	Specify second‐level
	Model specification	3dMEMA ‐set ‐missing_data 0 3dMVM^b^ ‐dataTable	Stats: Full model setup: EVs	Factorial design specification: One‐sample *T*‐test Full factorial^b^
	Model estimation	*Nothing to specify*	*Nothing to specify*	Model estimation
	Contrasts	*Nothing to specify*	Stats: Full model setup: contrasts	Contrast manager
	Second‐level inference	3dMask_Tool (obtain group‐mask) 3dClustSim 3dClust 3dCalc (Binarizing cluster masks and masking t_stat) 3dTcat (obtaining one image in a 4d volume)	Poststats	Results report
Results sharing	NIDM‐results export	*Not available*	nidmfsl	Results report
	NeuroVault upload	*Upload all statistic images*	*Upload of “group.gfeat.nidm.zip”*	Upload of “spm_****.nidm.zip”

Implementation of each of the processing steps (ds000001, ds000109, ds000120) within AFNI, FSL and SPM.

a The @SSWarper program was ran on each subject prior to afni_proc.py for brain extraction of the anatomical image, and to apply the nonlinear warp of the anatomy to MNI space.

b ds000120 only.

c Image calculator was used to create bain mask from grey matter, white matter, and CSF images; see text.

### 2.2 Data analyses

All data analyses were conducted using AFNI (version AFNI_18.1.09), FSL (version 5.0.10), and SPM (version SPM12, v6906). Computation was performed on a cluster comprised of 12 Dell PowerEdge servers (6 R410, 12 core 2.40 GHz processors, 6 R420, 12 core 2.80 GHz processors) running CentOS 7.3.

#### 2.2.1 Pipeline

A full decomposition of the pipelines implemented within the three packages for each study is presented in Table 1. Here, we give a brief description of the procedures.

In AFNI, preprocessing and subject‐level analyses were conducted using the @SSwarper program and afni_proc.py. For ds000001 and ds000109, we used the 3dMEMA program to perform a one‐sample *T*‐test, while for ds000120 we used the 3dMVM program at the second level to conduct a mixed‐effects analysis, generating an *F*‐statistic for the main effect of time.

In FSL, analyses were carried out using the FMRI Expert Analysis Tool (FEAT, v6.00). For each analysis, at the first level a separate .fsf file was created for each scanning session. Runs were then combined as part of a second level fixed‐effects model, yielding results which were subsequently inputted into a group analysis.

In SPM, preprocessing, subject‐ and group‐level analyses were conducted by selecting the relevant modules within SPM's Batch Editor. In particular, subject‐level and group‐level analyses were conducted using the specify first‐level and specify second‐level modules, respectively.

Once analyses were complete, the results for each software package were exported as NIDM‐Results packs (FSL and SPM only, [Maumet et al., 2016]) and uploaded to a public collection on the NeuroVault (RRID:SCR_003806, http://neurovault.org; [Gorgolewski et al., 2015]) online data repository.

#### 2.2.2 Common processing steps

A number of processing steps for each package were included in all of our analyses, regardless of whether they had been implemented in the original study. While this meant deviating from an exact replication of the original pipeline, these processing steps were either fundamental to ensure that results from each software package could be compared objectively, or steps that are widely accepted as best practice within the community. In this section we describe these steps.

Successful coregistration of the functional data to the structural brain images—and subsequently—registration to the MNI template, was of paramount importance to us for fair comparability of the results. During our first attempt at analysing the ds000001 dataset we discovered that seven subjects had essential orientation information missing from the NIfTI header fields of their functional and structural data. As the source DICOM files were no longer available, the original position matrices for this dataset were unable to be retrieved. This caused coregistration to fail for several subjects across all three software packages in our initial analysis of this data. We rectified the issue by manually setting the origins of the functional and structural data. OpenfMRI released a revision (Revision: 2.0.4) of our amended dataset which we used for the analysis. Further to this, we also set a number of common preprocessing steps within each package to be applied in all our analyses.

Firstly, brain extraction was conducted on the structural image in all software. We did this to improve registration and segmentation. In AFNI, brain extraction was carried out using 3dskullstrip, that was called implicitly from within the @SSwarper program. The skull‐stripped anatomical volume obtained here was inputted into our afni_proc.py scripts where further preprocessing and first‐level analyses were carried out. In FSL, brain extraction was performed on both the functional and structural data. The Brain Extraction Tool (BET; [Smith, 2002]) was applied to each structural image from the command line before preprocessing, and for functional data with the BET option within the Prestats module of FEAT. In SPM, brain extraction was implemented via the segmented structural images. Grey matter, white matter and CSF images were summed and binarised at 0.5 to create a brain mask, which was applied to the bias corrected structural image using the Image Calculator.

Coregistration of the functional data to the anatomy was carried out for the most part using the default settings in each software. In AFNI, alignment of the data was conducted using the align_epi_anat.py program called implicitly from the align block within the afni_proc.py scripts. We included the ‐volreg_align_e2a option within our scripts to specify alignment of the functional data onto the anatomy, as by default AFNI conducts the inverse transformation of anatomy onto functional. Further to this, we also added the ‐align_opts_aea program to all of our scripts with the ‐giant_move and ‐check_flip options to allow for larger transformations between the images. In FSL, coregistration was carried out within FEAT using the default linear registration methods with a Boundary‐Based Registration (BBR) cost function. The default methods were also applied within SPM's Coregister: Estimate module, using a normalised mutual information cost function.

Registration of the structural and functional data to the anatomical template was executed using each packages nonlinear settings. In AFNI, nonlinear registration of the anatomical data to the MNI template was conducted as part of the @SSwarper program ran prior to the afni_proc.py script. The warps computed by @SSwarper were passed to afni_proc.py using the ‐tlrc_NL_warpred_dsets option, and applied to the functional data within the tlrc block using the ‐volreg_tlrc_warp option. By default, the resampled functional data in MNI space has voxel size determined from the raw 4D data; we forced 2 mm cubic voxels with the ‐volreg_warp_dxyz option for compatibility with FSL and SPM's 2 mm default. In FSL, registration to the MNI template was conducted using FMRIB's Nonlinear Image Registration Tool (FNIRT; [Andersson et al., 2007]), controlling the degrees of freedom of the transformation with a warp resolution of 10 mm. In SPM, the nonlinear deformations to MNI space were obtained as part of the Segment module and then applied to the structural and functional data within the Normalise: Write module.

As a form of quality control, we created mean and standard deviation images of the subject‐level MNI‐transformed anatomical and mean functional images. Alongside the subject‐level data, these images were assessed to check that registration to MNI space had been successful. When intersubject registration failed remedial steps were taken within each software; these are described in the software implementation parts of the following study‐specific analysis sections.

Across all software packages six motion regressors were included in the analysis design matrix to regress out motion‐related fluctuations in the BOLD signal. Use of six or more derived motion regressors is commonly recommended as good practice, and we chose to use just six regressors as this could be easily implemented across software.

Finally, we note that each software package uses a different default connectivity criterion for determining significant clusters: 6‐connectivity for AFNI, 18‐connectivity for SPM, and 28‐connectivity for FSL. Since these settings are not typically modified we have kept these defaults in all of our analyses to reflect standard practices carried out within each software.

We now describe the task‐specific analysis procedures for each of the three studies as carried out in the original publications, and how these methods were implemented within each package. While we decided to keep the above steps of the analysis pipelines fixed, for all remaining procedures we attempted to remain true to the original study. Any further deviations necessitated are discussed in the software implementation sections. Notably, apart from the addition of six motion regressors, *all* of our common steps relate to preprocessing, and hence for first‐ and group‐level analysis we attempt to exactly replicate the original study.

#### 2.2.3 ds000001 analyses

In the publication associated with the ds000001 study all preprocessing and analysis was conducted within FSL (version 4.1.6). Data on all 16 subjects were available to us on OpenfMRI. In the original preprocessing, the first two volumes of the functional data were discarded and the highpass‐filter was set to a sigma of 50.0 s. Motion correction was conducted using MCFLIRT and brain extraction of the functional data was applied with BET, after which FSL's standard three‐step registration procedure was carried out to align the functional images to the structural scan. Spatial normalisation was implemented with FMRIB's Linear Image Registration Tool (FLIRT; [Jenkinson et al., 2002]), and data were smoothed using a 5 mm full‐width‐half‐maximum (FWHM) Gaussian kernel. At the run level, each of the events were convolved using a canonical double‐gamma haemodynamic response function (HRF); FEAT's (then newly available) outlier de‐weighting was used. Subject‐level analysis of the functional data were conducted using a general linear model (GLM) within FEAT, where a selection of the regressors were orthogonalized. The three scanning sessions for each participant were carried out separately and then combined together at the second level. A pair of one‐sided *T*‐tests were conducted at the group‐level to test for positive and negative effects separately. For each test, clusterwise inference was performed using an uncorrected cluster‐forming threshold of *p* < .01, FWE‐corrected clusterwise threshold of *p* < .05 using Gaussian random field theory.

We opted to not use outlier de‐weighting on the basis that such methods were impractical to implement across all software packages.

##### *AFNI implementation*

Using our default procedure for the AFNI analysis, we found that coregistration of the functional scans onto the anatomy failed for four subjects. To remedy this issue, for this study we modified our afni_proc.py scripts: Within the ‐align_opts_aea module, the “‐ginormous move” option was added to align centers of the functional and anatomical volumes, and the “‐cost lpc + ZZ” option was used to apply a weighted combination of cost functionals. Both of these changes are recommended for data with little structural detail. Following these modifications all coregistrations were successful.

To replicate the orthogonalization methods from the original study, a separate orthogonalization script was ran for each subject prior to the first‐level analyses. Within this script, the (un‐orthogonalized) regressors were generated by passing the event timing files to 3dDeconvolve, after which the 3dTproject command was used to obtain the desired projections. The orthogonalized regressor files outputted from this script were then entered into afni_proc.py to replicate the original subject‐level analysis model.

Trials were convolved with a single gamma HRF using either the BLOCK or dmBLOCK option within the ‐regress_basis_multi module, determined by whether the event file had fixed or variable duration times respectively. The ‐regress_stim_types option was added to our afni_proc.py script to specify event files for regressors which had been parametrically modulated in the original study, and identify the orthogonalized regressors.

At the group level, we performed a mixed‐effects analysis using 3dMEMA. The critical cluster size threshold was determined by Monte Carlo simulation with the 3dClustSim program.

##### *FSL implementation*

Implementation in FSL closely followed the original procedure described above, with the exception that nonlinear registration was used to transform the data to standard space.

##### *SPM implementation*

Implementation in SPM closely followed the pipeline outlined in Table 1.

#### 2.2.4 ds000109 analyses

The original preprocessing and statistical analysis for the ds000109 study was carried out using SPM8. Data were shared on 36 of the 40 subjects, 21 of which were young adult subjects that had fMRI data compatible for our reanalysis. First, functional data were realigned and unwarped to correct for head motion and geometric distortions. After transforming the data into a standardised space, the normalised data were smoothed with an 8 mm FWHM Gaussian kernel. Further to this, custom software was applied to exclude functional volumes where head motion had exceeded a certain limit; however, this process was omitted from our pipelines since this feature was not available in any of the software packages. The preprocessed data were entered into a GLM for first level analysis where trials were modelled using a block design and convolved using SPM's canonical HRF. Each participant's contrast images were then entered into a one‐sample group analysis using clusterwise inference, cluster forming threshold of *p* < .005, 5% level FWE using random field theory; in their analysis, this amounted to a critical cluster size threshold of 56 voxels.

##### *AFNI implementation*

Intersubject registration to the MNI atlas failed for one subject, for which part of the frontal lobe was missing. We addressed this by revising this study's AFNI pipeline to use the ‐pad_base 60 option within the ‐tlrc_opts_at module included in afni_proc.py. This gave extra padding to the MNI template so that no part of the functional image was lost during the alignment.

The HRF was modelled with SPM's canonical HRF using the SPMG1 option for each event within the ‐regress_basis_multi option and passing the duration of the regressor as an argument to the function.

At the group level, we performed a mixed‐effects analysis using 3dMEMA. *p*‐values were determined by Monte Carlo simulations with 3dClustSim.

##### *FSL implementation*

To recreate the original HRF model in FSL, we chose the Double‐Gamma HRF from the convolution options within FEAT.

##### *SPM implementation*

Implementation in SPM closely followed the original procedure described above.

#### 2.2.5 ds000120 analyses

A multi‐software analysis procedure was used for the ds000120 study, where data were preprocessed with FSL and then analysed using AFNI. fMRI data were shared on OpenfMRI for 26 of the original 30 subjects, and 17 had data available on the task of interest. This was the only study that applied slice‐timing correction, adjusting the functional data for an interleaved slice acquisition. Functional scans were realigned to the middle volume, and following brain extraction with BET, registered to the structural scan in Talairach space using FLIRT and FNIRT. Data were high‐pass filtered with a sigma value of 30.0 s and smoothed with a 5 mm FWHM Gaussian kernel. Like the previous study, further methods were used to remove functional volumes with excessive motion which have been left out from our analyses due to discordance across software. Subject‐level analysis was conducted within AFNI. To allow for flexible modelling of the response to the saccade task, this study used a HRF basis consisting of eight sine functions with a poststimulus window length of 24.0 s. At the group level, subjects were entered into a mixed‐effect model, with subjects as a random factor, trial type (reward, neutral) and time as within‐group factors, and age group (child, adolescent, adult) as a between‐group factor. Clusterwise inference was used on the main effect of time activation map (*F*_{8,142} statistic), cluster‐forming threshold of *p* < .001, controlling FWE at the 5% level, obtained with Monte Carlo methods. This computed critical cluster size threshold was 23 voxels.

For our replication exercise we only consider the main effect of time. This analysis is based on the corresponding time effect contrasts for each subject and requires a simpler model, with one random effect (subject) and one fixed effect (time).

##### *AFNI implementation*

Slice timing was conducted using the ‐tshift_opts_ts program within afni_proc.py with the ‐tpattern option applied to specify an interleaved slice acquisition.

The sine basis set used for the HRF was modelled using the ‐regress_basis_multi module with the SIN option.

At the group level, a mixed‐effect analysis was carried out with the 3dMVM program. Following this, 3dClustSim was used to obtain the cluster extent corresponding to the original study threshold. In our analysis we found the cluster size threshold to be 48 voxels.

##### *FSL implementation*

The repeated‐measures design used in the group‐level analysis of the original study was not feasible to implement for parametric inference in FSL, and as such, we did not attempt an FSL reanalysis for this study. (The FEAT manual does describes “Repeated Measures” examples, but these are based on a restrictive assumption of compound symmetry; here this would entail assuming that all 8*7/2 = 28 correlations among the basis regression coefficients are equal.)

##### *SPM implementation*

Slice timing was conducted using the Slice Timing module within the Batch Editor of SPM.

Although an exact equivalent of the original HRF model was not possible in SPM, we chose the closest equivalent using the Fourier basis set with an order of 4, leading to a total of 9 basis functions fit to each of the reward and neutral conditions for each of the three runs. A set of 9 first level contrasts computed the average Fourier coefficients over conditions and runs.

To reproduce the group‐level analysis in SPM, a full factorial design was chosen within the “Factorial design specification” module of the Batch Editor, with a time factor (9 levels) and adding age‐group to the model using two covariates (adolescent vs. child, adult vs. child); the main effect of time was tested with an *F*‐contrast.

### 2.3 Comparison methods

We applied three separate quantitative methods to measure the similarity between the group results obtained within each software package for each of the three studies.

Firstly, Bland–Altman plots comparing the unthresholded group statistic maps were created for each pairwise combination of software packages. These plotted the difference between the statistic values (*y*‐axis) against the mean statistic value (*x*‐axis) for all voxels lying inside the intersection of the two software's analysis masks. The plots provide an assessment of the level of agreement between two software packages about the magnitude of the statistic value observed at each voxel. If two software packages were in perfect agreement, all points on the bland–altman plot would lie on the *x*‐axis, since the difference between the statistic values at each voxel would be zero. The degree of disagreement is therefore evaluated by the perpendicular distance of points from the *x*‐axis; for example, for a “AFNI‐FSL” Bland–Altman plot, points above *x*‐axis are where AFNI's statistic is larger than FSL's. With the difference plotted against the average, general patterns of disagreement can be discerned.

In addition to this, we also created Bland–Altman plots to compare percentage BOLD change maps (for ds000120, partial *R* ^2^ maps) between software. For each package, an appropriate normalisation of the group‐level beta maps was conducted to convert to percentage BOLD change units. Due to differences in how each package scales the data, a different normalisation was required for each of the three packages. For ds000120, the partial *R* ^2^ maps were computed via a transformation of the group‐level *F*‐statistic images. We provide full details on how each of these procedures were carried out in Appendix (Appendix S1. for percent BOLD change, Appendix S2. for partial *R* ^2^). In all of our Bland–Altman comparisons, we excluded white matter and cerebral spinal fluid voxels according to the MNI tissue probability maps thresholded at 0.5.

We also computed the Dice similarity coefficient for each pairwise combination of the group‐level thresholded statistic maps. The coefficient is calculated as the cardinality of the intersection of the thresholded maps divided by the average of the cardinality of each thresholded map. While Bland–Altman is interested in the similarity between statistic values, Dice measures the overlap of voxels as a means to assess the *spatial similarity* of activated clusters. The coefficient takes a value between 0 and 1, where one indicates complete congruence between the size and location of clusters in both thresholded maps, while zero indicates no agreement. Dice coefficients were computed over the intersection of the pair of analysis masks, to assess only regions where activation could occur in both packages. We also calculated the percentage of “spill over” activation, that is, the percentage of activation in one software's thresholded statistic map that fell outside of the analysis mask of the other software.

A particular concern we had was that a pair of statistic images could in essence be very similar, but differs by a scale factor over all voxels. Another possibility was that one software could have greater sensitivity for voxels where signal was present, causing differences between images only for relatively higher statistical values. Both of these features would not be identifiable using our previous comparison methods. To address this, we computed the Euler Characteristic (EC) for each software's group *T*‐statistic map (*F*‐statistic for ds000120), thresholded using *T*‐values between −6 and 6 (0–6 for ds000120; increasing with an increment of 0.2). Alongside the EC, we also computed the number of clusters in the statistic images using the same thresholds. For a given threshold *t*, the EC calculates the number of clusters minus the numbers of “handles” plus the number of “holes” in the thresholded image. For large *t*, we expect the handles and holes to disappear, and therefore the EC provides an approximation of the number of clusters in an image. For smaller *t*, we expect our thresholded image to be one connected cluster with many holes and handles (like Swiss cheese)—it is in this situation where the EC is clearly more informative about differences between images than the cluster count alone. Over all *t*, the EC curve provides a signature of an entire statistic image, and provides a means to assess whether only superficial scaling differences are responsible for disparities between a pair of images.

For a qualitative assessment of whether similar activation patterns were displayed between packages, a NeuroSynth (RRID:SCR_006798, http://neurosynth.org) association analysis was conducted on each software's unthresholded statistic maps. These analyses performed a cognitive decoding of the unthresholded statistic image with images in the NeuroSynth database, to find the words or phrases most strongly associated with the activation patterns found in the statistic map.

Finally, we visually compared the corresponding slices of each software's thresholded statistic map to those presented in the publication figure we had attempted to recreate. Ensuring we had found activation in approximately the same regions as the original publication gave us an indication that we had successfully replicated the study's analysis pipeline.

### 2.4 Permutation test methods

For ds000001 and ds000109, in parallel to our replication analyses we computed an additional set of group‐level results applying nonparametric permutation test inference procedures available within each software package (a one‐sample repeated measures permutation test needed for ds000120 was not available in AFNI). The first level contrast maps obtained from our initial replications for each subject were entered into a group‐level one‐sample *T*‐test where clusterwise inference was conducted using the same cluster‐forming thresholds, and then 5% level FWE corrected thresholds were computed by permutation, using 10,000 permutations.

##### *AFNI implementation*

In AFNI, permutation inference was carried out using the 3dttest++ module with the ‐ClustSim option. By applying this option, permutation generated noise realisations which 3dClustSim used to generate cluster‐threshold tables. Significant clusters in the group‐activation map were found with 3dclust, using a critical cluster size threshold extracted from the 3dClustSim output.

##### *FSL implementation*

Permutation test inference was conducted in FSL using randomise version 2.9 (Winkler et al., 2014). This outputted a “corrp” image which was then used to mask the raw *T*‐statistic image to show significant voxels for the appropriate thresholds.

##### *SPM implementation*

The Statistical nonParametric Mapping (SnPM, version SnPM13; RRID:SCR_002092; [Nichols and Holmes, 2002]) toolbox was used to carry out permutation tests in SPM. The “MultiSub: one‐sample *T‐*test on diffs/contrasts,” Compute and Inference modules within SnPM were applied to obtain the final group‐level activation maps.

Each of the comparison methods described in the previous section were also applied to our permutation results to assess cross‐software differences for nonparametric inference methods. In addition, we also generated intrasoftware Bland–Altman plots and Dice coefficients to understand differences between the parametric and nonparametric methods applied within each package.

These methods were excluded for ds000120, since it was not possible to conduct permutation inference for an *F*‐test within AFNI, and parametric inference was unfeasible in FSL for this study as discussed in the previous section.

### 2.5 Scripting of analyses and figures

AFNI and FSL scripts were written in Python 2.7.14 and SPM scripts were written in Matlab R2016b. Scripts were made generalizable, such that the only study‐specific differences for each of the analyses in a software package were the raw data and working directory inputs, subject‐ and group‐level analysis templates (as well as a run‐level template for FSL), and a unique conditions structure necessary for creating the onset files for the specified study. For each analysis package, a script was written to extract the stimulus timings from the raw data to create event files that were compatible within the software. Subject‐level analysis templates were batch scripts created for each study containing all processing steps of the subject analysis pipeline for the respective software, with holding variables used where subject‐ or run‐specific inputs were required. The main script would take the template as an input, and cycling through each of the subjects, replace the holding variables with appropriate pathnames to create distinct batch scripts for each subject. These were then executed to obtain subject‐level results for all participants in the study.

A Python Jupyter Notebook (Kluyver et al., 2016) was created for each of the three studies. Each notebook harvests our results data from NeuroVault and applies the variety of methods discussed in the previous section using NiBabel 2.2.0 (Brett et al., 2017), NumPy 1.13.3 (Walt et al., 2011), and Pandas 0.20.3 (McKinney and Others, 2010) packages. Figures were created using Matplotlib 2.1.0 (Hunter, 2007) and Nilearn 0.4.0 (Abraham et al., 2014).

## 3 RESULTS

All scripts and results are available through our Open Science Framework (OSF; Erin D. Foster, 2017) Project at https://osf.io/U2Q4Y/ (Bowring et al., 2018a), and group‐level statistic maps used to create the figures in this section are available on NeuroVault: https://neurovault.org/collections/4110/, https://neurovault.org/collections/4099/, https://neurovault.org/collections/4100/, for ds0000001, ds000109, and ds000120, respectively. All analysis scripts, results reports, and notebooks for each study are available through Zenodo (Nielsen and Smith, 2014) at https://doi.org/10.5281/zenodo.1203654 (Bowring et al., 2018b).

Registration of each subject's functional data onto the anatomy was visually assessed. The mean and standard deviation images of the MNI structural and (mean) functional data (Figure S1, note that axial slices are slightly different between software due to different bounding boxes of the images) substantiate that registration was successful in all packages across the three studies.

### 3.1 Cross‐software variability

While qualitatively similar, variability in *T*‐statistic values and locations of significant activation was substantial between software packages across all three studies.

**Figure 1:**
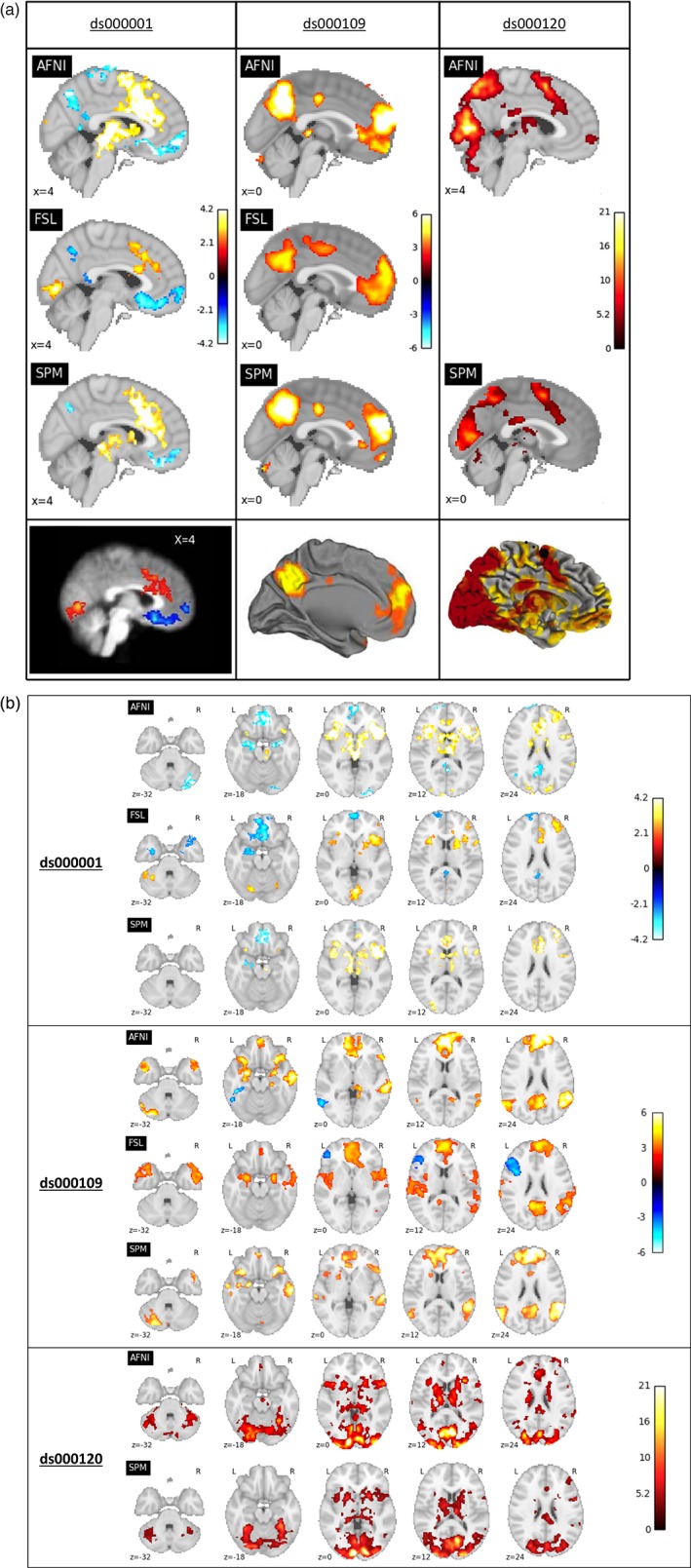
(a) Comparison of the thresholded statistic maps from our reanalysis with the main figures from each of the three publications. Left: For ds000001 data, thresholded *T*‐statistic images contrasting the parametric modulation of pumps of reward balloons versus the parametric modulation of the control balloon; beneath, a sagittal slice taken from fig. 3 in Schonberg et al. (2012). Middle: For ds000109, thresholded *T*‐statistic maps of the false belief versus false photo contrast; beneath, a midsagittal render from Moran et al. (2012). Right: For ds000120, thresholded *F*‐statistic images of the main effect of time contrast; beneath, a midsagittal render from fig. 3 in Padmanabhan et al. (2011). Note that for ds000109 and ds000120 the publication's figures are renderings onto the cortical surface while our results are slice views. While each major activation area found in the original study exists in the reanalyses, there is substantial variation between each reanalysis. (b) Comparison of the thresholded statistic maps from our reanalysis displayed as a series of axial slices. Top: ds000001’s thresholded *T*‐statistic maps contrasting parametric modulations of the reward balloons versus pumps of the control balloons. Middle: ds000109’s thresholded *T*‐statistic maps of the false belief versus false photo contrast. Bottom: ds000120’s thresholded *F*‐statistic maps of the main effect of time contrast. This figure complements the single slice views shown in Figure 1 [Color figure can be viewed at http://wileyonlinelibrary.com]

Comparisons of the thresholded results with the published findings are shown in Figure 1a, with further multi‐slice comparisons across software in Figure 1b (also in Figures S2, S4, and S6). The ds000001 study described positive activation in the bilateral anterior insula, dorsal anterior cingulate cortex, and right dorsolateral prefrontal cortex, and negative activation in the ventromedial prefrontal cortex and bilateral medial temporal lobe. In our reanalysis (Figure 1a, left) all three software found activation in these set of regions, with the exception that decreases in the medial temporal lobe were unilateral in FSL and SPM (left only). FSL also detected a visual response that neither AFNI or SPM picked up on (Figure 1a, left, and Figure 1b, top, *z* = 0 slice).

The ds000109 study reported activations in the bilateral temporoparietal junction. (TPJ), precuneus, anterior superior temporal sulcus (aSTS), and dorsal medial prefrontal cortex (dmPFC). Similar activations from our reanalyses are seen in Figure 1a, middle, although FSL only found activation in the right TPJ and aSTS. Further comparisons shown in Figure 1b, middle, highlight disagreement in the results: AFNI and FSL detected significant deactivations in distinct brain regions (inferior temporal gyrus for AFNI, inferior frontal gyrus for FSL), while SPM did not determine *any* significant deactivation. FSL also found a positive response in the superior temporal gyrus (STG) where AFNI and SPM did not (Figure 1b, middle, *z* = 0 and *z* = 12 slices).

**Figure 2:**
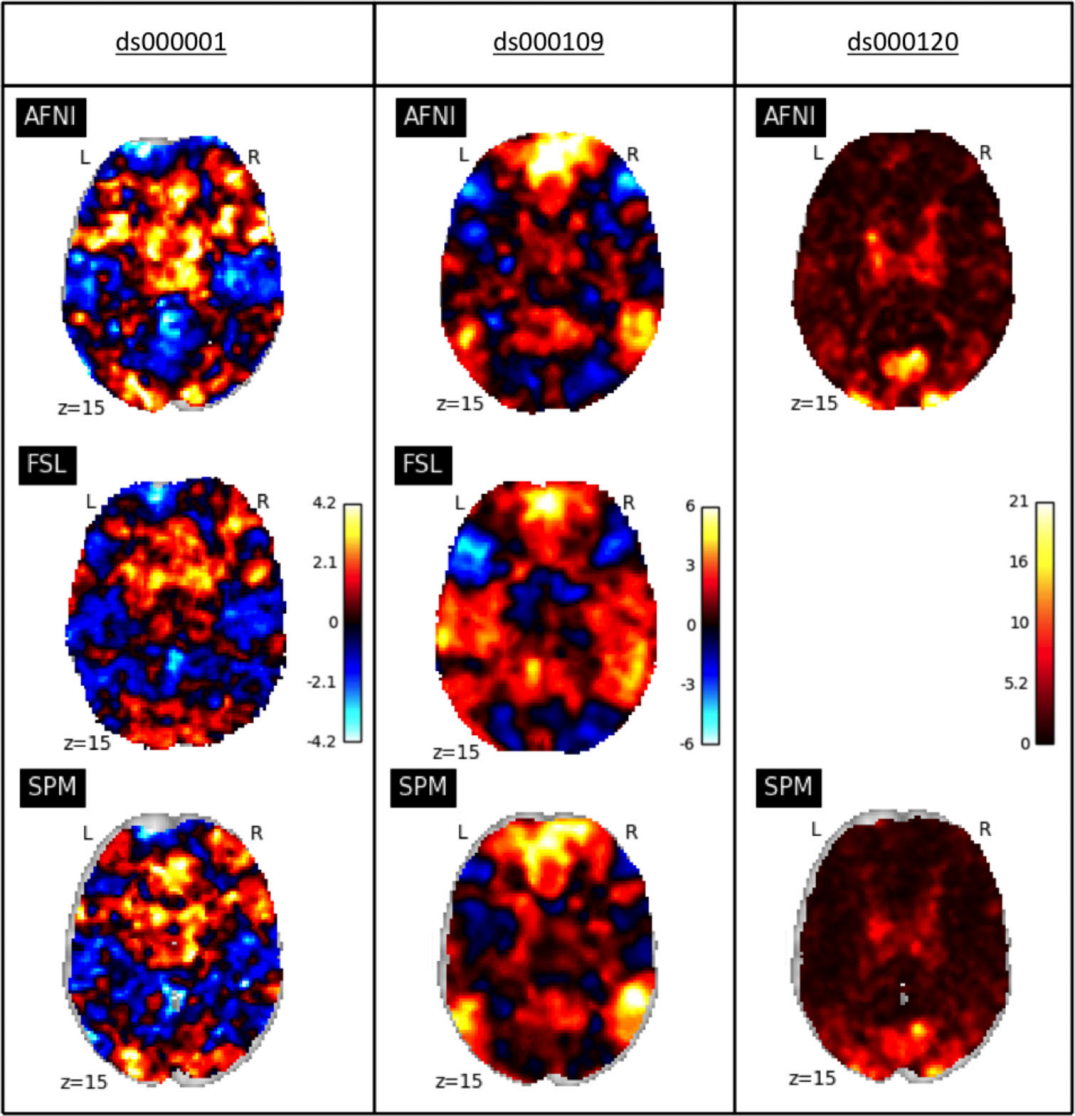
Comparison of the unthresholded statistic maps from our reanalysis of the three studies within each software package. Left: ds000001’s unthresholded *T*‐statistic maps of the parametric modulation of pumps of reward balloons versus the parametric modulation of the control balloon contrast. Middle: ds000109’s unthresholded *T*‐statistic maps of the false belief versus false photo contrast. Right: ds000120’s unthresholded *F*‐statistic maps of the main effect of time contrast. While areas of strong activation are somewhat consistent across all three sets of reanalyses, there is substantial variation in nonextreme values [Color figure can be viewed at http://wileyonlinelibrary.com]

The original ds000120 study found extensive activations for the main effect of time—the frontal, supplementary, posterior parietal cortex, basal ganglia, prefrontal cortex, ventral striatum, and orbitofrontal cortex all showed significant activation. Our reanalyses (Figure 1, right) are consistent with these findings, with the exception that neither AFNI nor SPM exhibited orbitofrontal (OFC) activation (though, the SPM analysis mask had poor OFC coverage). AFNI's *F*‐statistic values look to be generally larger than SPM here (Figure 1b, bottom, *z* = 0 and *z* = 12 slices). The unthresholded statistic maps from our reanalyses (Figure 2, Figures S7, S9, and S11) also show that while extreme values display moderate agreement, there are considerable differences across the brain in each given study.

NeuroSynth association analyses conducted on the unthresholded T‐statistic maps (Table 2) show that the most strongly related term to the activation patterns displayed across all three sets of results was the same: “anterior insula” for each software's ds000001 map, “medial prefrontal” for ds000109, and “visual” for ds000120. Phrases related to the task paradigm used in each study (“goal” for ds000001, “theory mind” for ds000109, “visual” for ds000120) were found across all software's activation patterns, alongside a range of common anatomical terms.

**Table 2:** Neurosynth analyses

	AFNI		FSL		SPM	
	Neurosynth analysis	Corr.	Neurosynth analysis	Corr.	Neurosynth analysis	Corr.
**ds000001**	Anterior insula	0.359	Anterior insula	0.240	Anterior insula	0.322
	Insula	0.276	**Task**	0.233	Anterior	0.245
	Anterior	0.243	**Tasks**	0.203	Insula	0.240
	Insula anterior	0.233	Parietal	0.190	**Goal**	0.229
	Thalamus	0.221	**Goal**	0.188	**Task**	0.225
	**Goal**	0.211	**Working memory**	0.184	Insula anterior	0.214
	**Pain**	0.198	**Working**	0.181	Thalamus	0.201
	Supplementary	0.197	Basal ganglia	0.173	Acc	0.199
	Premotor	0.196	Ganglia	0.172	Anterior cingulate	0.196
	Anterior cingulate	0.192	Basal	0.169	Ganglia	0.188
**ds000109**	Medial prefrontal	0.422	Medial prefrontal	0.355	Medial prefrontal	0.361
	Medial	0.381	Medial	0.309	**Theory mind**	0.331
	**Default**	0.366	Default	0.301	**Default**	0.329
	**Theory mind**	0.348	Posterior cingulate	0.299	Precuneus	0.314
	**Default mode**	0.341	**Default mode**	0.290	**Default mode**	0.310
	Precuneus	0.334	**Social**	0.282	Medial	0.301
	Posterior cingulate	0.327	Cingulate	0.275	**Mind**	0.296
	**Social**	0.322	**Theory mind**	0.270	Prefrontal	0.294
	**Mind**	0.311	**Resting**	0.261	**Mind tom**	0.289
	**Mind tom**	0.287	Precuneus	0.259	Posterior cingulate	0.287
**ds000120**	**Visual**	0.377			**Visual**	0.481
	v1	0.317			Occipital	0.367
	Occipital	0.293			v1	0.340
	**Eye**	0.261			Visual cortex	0.267
	**Eye movements**	0.252			**Spatial**	0.248
	Visual cortex	0.243			Spl	0.245
	Early visual	0.241			**Eye**	0.242
	**Spatial**	0.232			Early visual	0.238
	**Task**	0.229			Lingual	0.238
	Parietal	0.222			Intraparietal	0.237

The Neurosynth analysis terms most strongly associated (via Pearson correlation) to each software's group‐level statistic map across the three studies. Nonanatomical terms are shown in bold.

**Figure 3:**
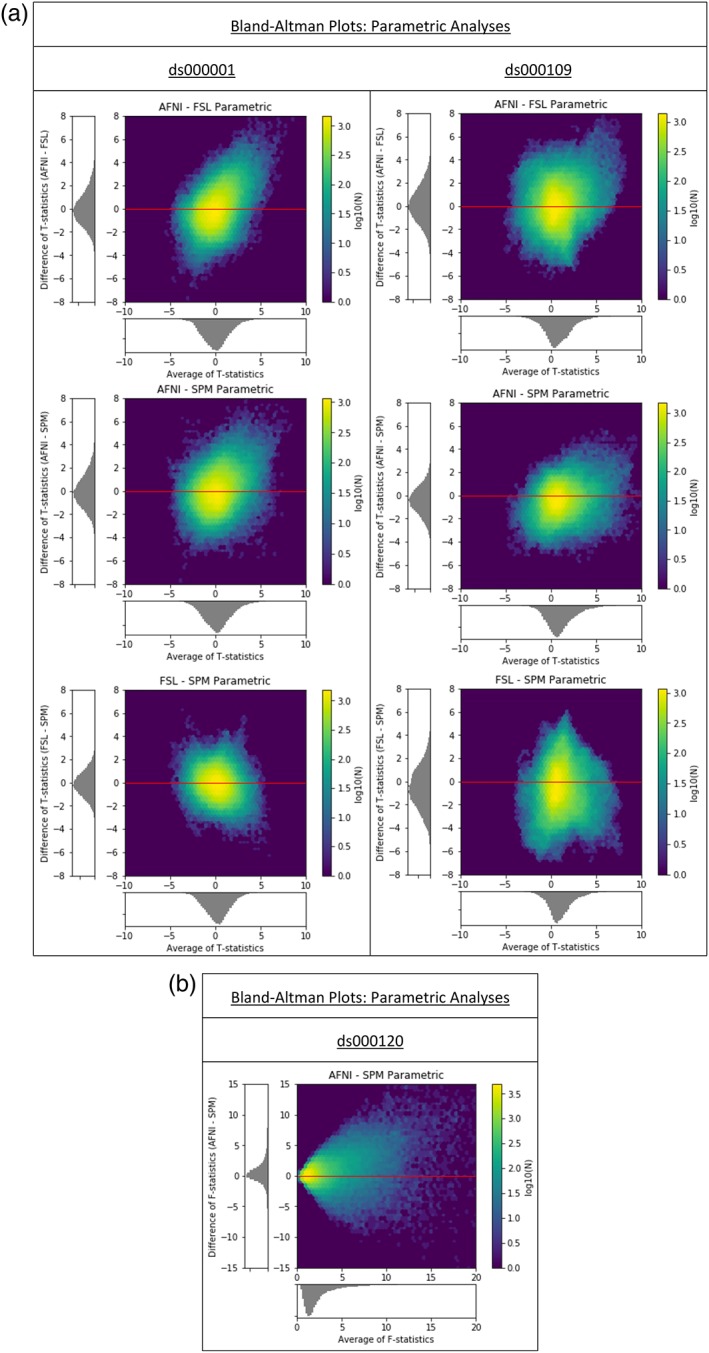
(a) Cross‐software Bland–Altman 2D histograms comparing the unthresholded group‐level *T*‐statistic maps computed as part our reanalyses of the ds000001 and ds000109 studies within AFNI, FSL, and SPM. Left; Comparisons for ds000001’s balloon analog risk task, *T*‐statistic images contrasting the parametric modulation of pumps of the reward balloons versus parametric modulation of pumps of the control balloon. Right; Comparisons for ds000109’s false belief task, *T*‐statistic images contrasting the false belief versus false photo conditions. Density images show the relationship between the average *T*‐statistic value (abscissa) and difference of *T*‐statistic values (ordinate) at corresponding voxels in the unthresholded Tstatistic images for each pairwise combination of software packages. While there is no particular pattern of bias, as the *T*‐statistic differences are centered about zero, there is remarkable range, with differences exceeding ±4 in all comparisons. (b) Cross‐software Bland‐Altman 2D histogram comparing the unthresholded main effect of time Fstatistic maps computed in AFNI and SPM for reanalyses of the ds000120 study. The differences are generally centered about zero, with a trend of large *F*‐statistics for AFNI. (The funnel‐like pattern is a consequence of the *F*‐statistic taking on only positive values) [Color figure can be viewed at http://wileyonlinelibrary.com]

Figure 3a compares statistic values across packages using Bland–Altman plots (rendered as 2D histograms) for ds000001 and ds000109. The distribution of the pairwise differences in *T*‐statistics (*y*‐axis) is generally centered about zero, indicating no particular bias; however, there is substantial variation here, with *T*‐statistic differences exceeding 4.0 in magnitude. Pairwise correlations ranged from 0.429 to 0.747 for intersoftware comparisons (Table 3). The Bland–Altman plots comparing percentage BOLD change maps (Figure S13) are more conclusive, showing a clear trend for SPM to report larger effect estimates than the other two packages. Figure 3b presents the Bland–Altman plot comparing unthresholded *F*‐statistic images for ds000120, which has a very different appearance due to *F*‐statistics being nonnegative. The corresponding Bland–Altman plot comparing partial *R* ^2^ values (Figure S14) for this study is similar in shape. Broadly speaking, while there are no gross differences in sensitivity, there is a slight tendency for AFNI's extreme statistics to exceed FSL's and SPM's, and SPM's to exceed FSL's, most evident in ds000109.

**Table 3:** Summary of test statistics mean differences and correlations for each pair of test statistic images

		ds000001		ds000109		ds000120	
		Mean diff.	Corr	Mean diff.	Corr	Mean diff.	Corr
AFNI vs. FSL	Parametric	0.009	0.616	0.035	0.585		
	Nonparametric	0.271	0.577	0.006	0.573		
AFNI vs. SPM	Parametric	0.061	0.614	−0.490	0.747	0.415	0.748
	Nonparametric	−0.096	0.628	−0.445	0.787	n/a	n/a
FSL vs. SPM	Parametric	−0.047	0.684	−0.529	0.429		
	Nonparametric	−0.479	0.720	−0.439	0.438		
AFNI	Para. vs. NonP.	0.155	0.984	−0.048	0.981		
FSL	Para. vs. NonP.	0.382	0.844	−0.064	0.946		
SPM	Para. vs. NonP.	0.000	1.000	0.000	1.000		

Mean differences correspond to the *y*‐axes of the Bland–Altman plots displayed in Figures 3a,b, and 7. Each mean difference is the first item minus second; for example, AFNI versus FSL mean difference is AFNI‐FSL. Correlation is the Pearson's *r* between the test statistic values for the pair compared. Intersoftware differences are greater than intrasoftware.

**Figure 4:**
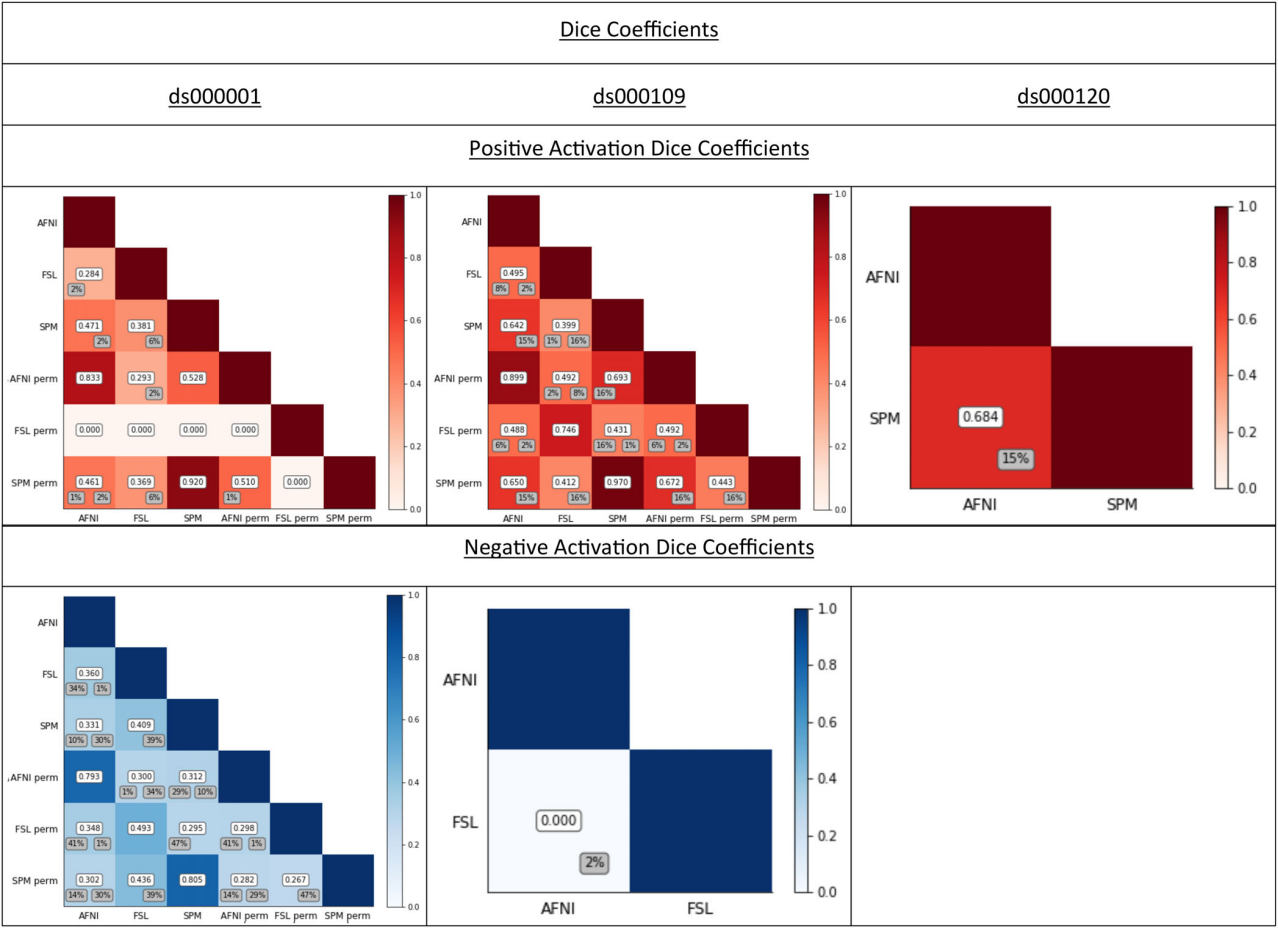
Dice coefficients comparing the thresholded positive and negative *T*‐statistic maps computed for each pair of software package and inference method for each of the three reproduced studies. Dice coefficients were computed over the intersection of the pair of analysis masks, to assess only regions where activation could occur in both packages. Percentage of “spill over” activation, that is, the percentage of activation in one software's thresholded statistic map that fell outside of the analysis mask of the other software is displayed in grey; left value for row software, right value for column software. For ds000001 increases, FSL permutation obtained no significant results, thus generating Dice coefficients of zero; for ds000109 decreases, only AFNI and FSL parametric obtained a result and hence only one coefficient is displayed. Dice coefficients are mostly below 0.5, parametric‐nonparametric intrasoftware results are generally higher; ds000120’s *F*‐statistic results are notably high, at 0.684, perhaps because it is testing a main effect with ample power [Color figure can be viewed at http://wileyonlinelibrary.com]

Spatial localization of significant activation in the thresholded *T*‐statistic images also varied across software packages. Figure 4 shows the Dice coefficients for all pairs of analyses (parametric results are presented in first 3 rows of larger triangles). For ds000001, the average value of Dice coefficients comparing locations of activations across reanalyses is 0.379. These values improve for ds000109, where the mean Dice coefficient for positive activations is 0.512. Here, AFNI and FSL were the only software packages to report significant negative clusters for the ds000109 study. Strikingly, these activations were found in completely different anatomical regions for each package, witnessed by the negative activation AFNI/FSL dice coefficient of 0. Finally, the AFNI/SPM Dice coefficient for the thresholded *F*‐statistic images obtained for ds000120 is 0.684; it is notable that across all studies, the AFNI/SPM dice coefficients are consistently the largest.

Spill over values, given by the grey values beneath the dice coefficients in Figure 4, are generally largest for SPM comparisons. They are particularly prominent in the negative activation plot for ds000001, where there is at least 30% spill over for all parametric pairwise comparisons, the largest being 39% for SPM/FSL. Recalling that these values are the percentage of activation which occurred within one package that was outside the other package's analysis mask, this is likely due to the fact SPM consistently had the smallest analysis mask out of the three packages, while FSL had the largest. In our ds000001 reanalyses, SPM's group‐level analysis mask was made up of 175,269 voxels, while AFNI's had 198,295 voxels and 251,517 for FSL. For ds000109, SPM's group‐level mask contained 178,461 voxels compared to AFNI's 212,721 and FSL's 236,889. Finally, for ds000120, SPM had 174,059 voxels to AFNI's 208,340. Note FSL's mask image has slightly but consistently more nonzero voxels than in its statistical result images).

**Figure 5:**
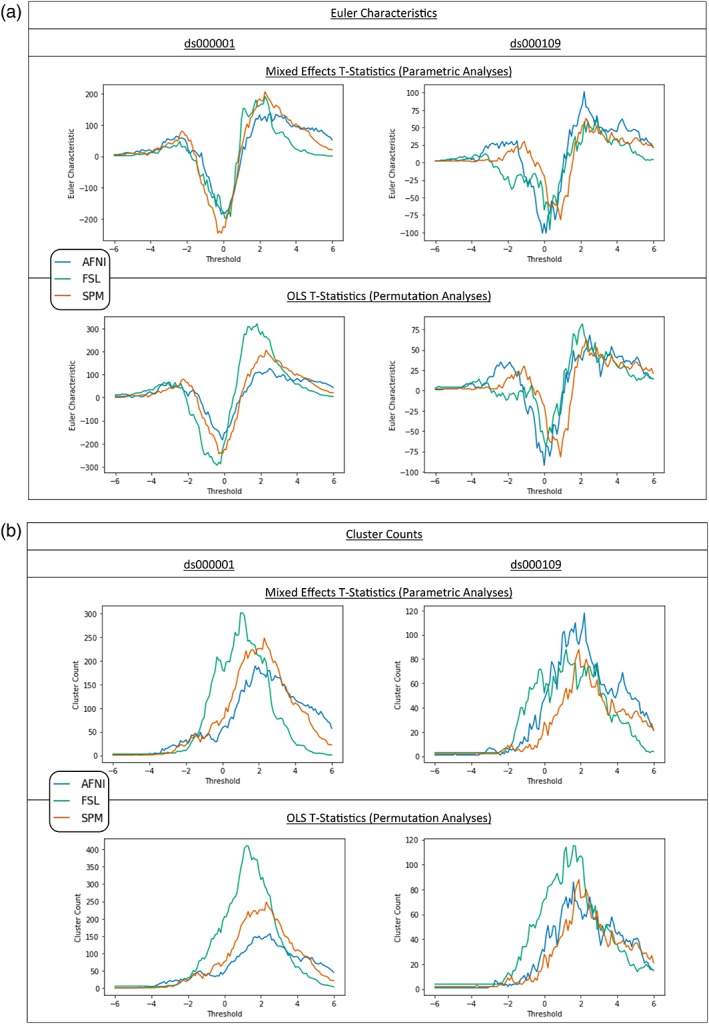
(a) Euler characteristic (EC) plots for ds000001 and ds000109. On top, comparisons of the Euler characteristic computed for each software's *T*‐statistic map from our reanalyses using a range of *T*‐value thresholds between −6 and 6. Below, comparisons of the ECs calculated using the same thresholds on the corresponding *T*‐statistic images for permutation inference within each package. For each *T*‐value the EC summarises the topology of the thresholded image, and the curves provide a signature of the structure of the entire image. For extreme thresholds the EC approximates the number of clusters, allowing a simple interpretation of the curves: For example, for ds000001 parametric analyses, FSL clearly has the fewest clusters for positive thresholds. (b) Cluster count plots for ds000001 and ds000109. On top, comparisons of the number of cluster found in each software's *T*‐statistic map from our reanalyses using a range of *T*‐value thresholds between −6 and 6. Below, comparisons of the cluster counts calculated using the same thresholds on the corresponding *T*‐statistic images for permutation inference within each package [Color figure can be viewed at http://wileyonlinelibrary.com]

Further evidence of spatial variability is also exhibited by the Euler characteristic (EC) plots for the parametric analyses presented in Figure 5a, top (and Figure S12 for ds000120), complemented by the cluster count plots in Figure 5b, top. We note that because the EC plots were created by thresholding each software's statistic map at a fixed range of *T*‐values, without the computation of *p*‐values, differences between the parametric and nonparametric EC curves are due to the different first level models used in each case (mixed‐effects for parametric, OLS for nonparametric), and not due to the actual parametric and nonparametric inference procedures used to obtain *p*‐values. Since the EC counts the number of clusters minus the number of “handles” plus the numbers of “holes” in an image, for large thresholds we expect the EC to closely approximate the number of clusters of significant activation present in the equivalent thresholded map. This is confirmed by Figures 5a,b, both figures showing that across the two studies FSL had a smaller number of activated clusters at larger thresholds. For ds000001, the peak cluster count value (Figure 5b, top left) occurs at a lower threshold for FSL. This plot suggests that in general FSL's *T*‐statistic values were more liberal here—the initial rise of the FSL curve signifies the *T*‐statistic image breaking up into clusters at lower thresholds than AFNI and SPM, and then as the clusters begin to get “thresholded out” this causes the FSL curve to dip below the other two packages. The EC plots highlight overall topological differences in the statistic maps: If the images were the same up to an image‐wide monotonic transformation, this would be revealed by the EC curves having the same general shape but with some portions shifted or compressed. In this sense, the distinct shapes seen in portions of the curves (e.g., for ds000109, negative thresholds) suggest differences in the topologies of each softwares activation pattern.

### 3.2 Cross‐software variability for nonparametric inference

Consistent with the parametric inference results, activation localization and statistic values varied greatly between packages for the permutation test results computed for ds000001 and ds000109.

Before reviewing statistic map comparisons, we stress that the goal of these nonparametric analyses is to obtain FWE‐corrected cluster *p*‐values with weaker assumptions. Thus the permutation test unthresholded statistic maps are not “nonparametric” maps, but rather usual one‐sample *T*‐test maps that form the basis of permutation analyses. While SPM's parametric analysis uses the same one‐sample *T*‐test, AFNI's and FSL's parametric models use a mixed‐effects model and weighted least squares. Hence all comparisons of the nonparametric test statistic values (in contrast to thresholded maps) do not convey information about nonparametric inference per se, but compare different preprocessing and first level modelling from the three packages while holding the second level model constant.

Quantitative assessment with Dice coefficients are shown in Figure 4 (“perm” vs. “perm” cells) and—in accordance with the parametric results—are generally poor. Like the parametric analyses, AFNI/SPM Dice values are altogether better than the other comparisons. For ds000001, FSL's nonparametric method found no significant clusters, and thus all Dice coefficients connected to this analysis are zero. However, note that the significant regions found in the other parametric and nonparametric results for this study mostly comprise of a single activation cluster spanning the lateral and medial frontal cortex, insular cortex, basal ganglia, and brainstem—an extensive and irregularly‐shaped cluster that could easily become disconnected and thus lose significance. As before, ds000109 Dice values are also generally better than ds000001.

**Figure 6:**
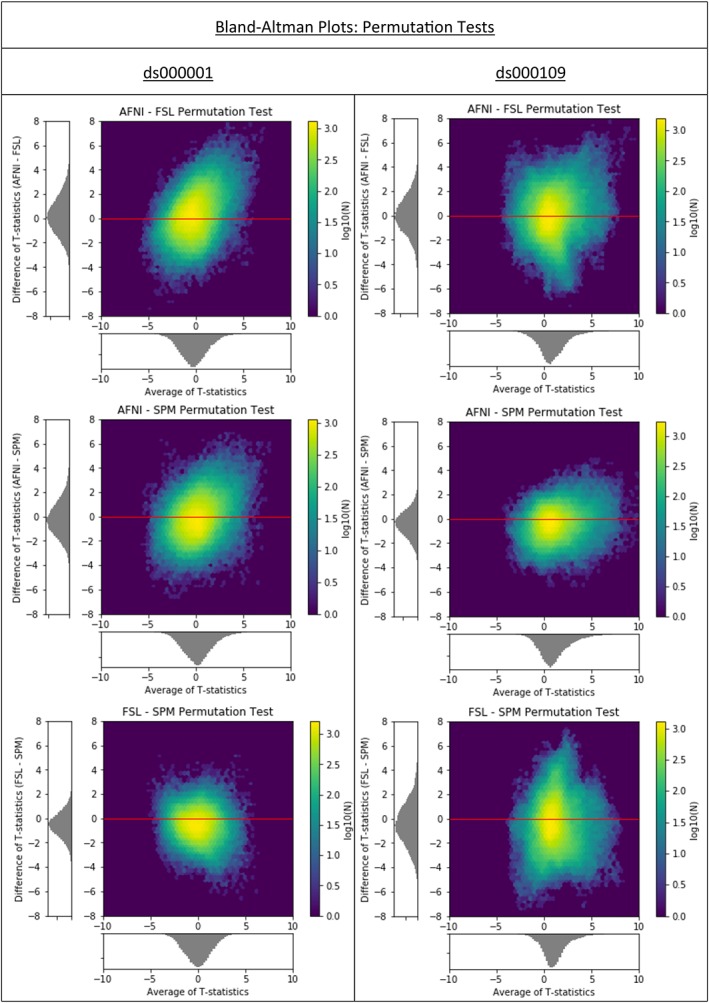
Cross‐software Bland–Altman 2D histograms for the ds000001 and ds000109 studies comparing the unthresholded group‐level *T*‐statistic maps computed using permutation inference methods within AFNI, FSL, and SPM. Similar to the results obtained using parametric inferences in Figure 3, all of the densities indicate large differences in the size of activations determined within each package [Color figure can be viewed at http://wileyonlinelibrary.com]

The nonparametric Bland–Altman plots (Figure 6) show substantial spread qualitatively similar to the parametric ones (Figure 3), and correlations between statistics maps are similar for nonparametric in congruence with the parametric comparisons (Table 3). EC curves (Figure 5, bottom) again exhibit considerable topological variation between software packages. Notably, while AFNI and SPMs EC curves are relatively similar across choice of inference method, FSL permutation inference determined substantially more clusters than parametric for low positive thresholds in both studies (Figure 5a,b, bottom).

### 3.3 Intrasoftware variability, parametric versus nonparametric

Comparisons of parametric and permutation test inference results within each package hold all preprocessing and first level modelling constant, only varying the second level model and inference procedure. The level of agreement between the two inferences *within* each package varied greatly across software. Before making comparisons, we note that since SPM's parametric and nonparametric inference share the same group level model, the unthresholded statistic images produced using each inference model are identical here.

The thresholded statistic maps are generally similar within each of the software packages (ds000001: Figures S2 vs. S3; ds000109: Figures S4 vs. S5), with the exception of FSL's nonparametric inference “decreases‐only” finding for ds000001. Unthresholded maps are notably more similar for ds000109 (Figures S9 vs. S10) than for ds000001 (Figures S7 vs. S8), again noting that SPM's pairs of maps here are identical.

**Figure 7:**
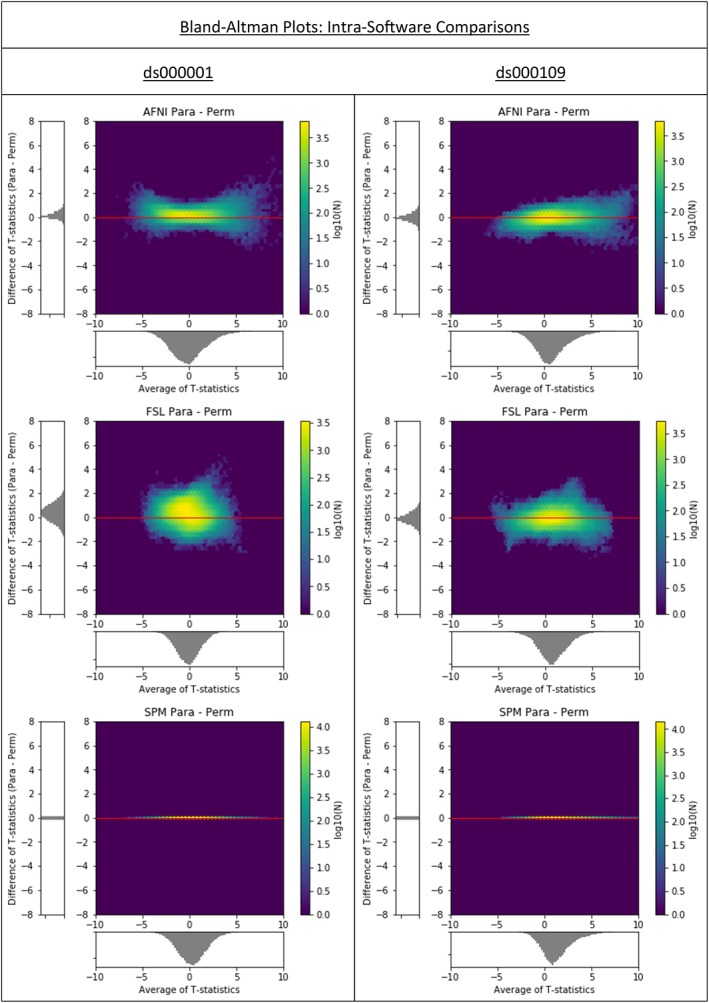
Intrasoftware Bland–Altman 2D histograms for the ds000001 and ds000109 studies comparing the unthresholded group‐level *T*‐statistic maps computed for parametric and nonparametric inference methods in AFNI, FSL and SPM. Each comparison here uses the same preprocessed data, varying only the second level statistical model. SPM's parametric and nonparametric both use the same (unweighted) onesample *T*‐test, and thus show no differences. AFNI and FSL's parametric models use iterative estimation of between subject variance and weighted least squares and thus show some differences, but still smaller than between‐software comparisons [Color figure can be viewed at http://wileyonlinelibrary.com]

Bland–Altman plots (Figure 7) reveal much greater levels of parametric‐nonparametric agreement, with AFNI displaying greater agreement than FSL. For FSL, we selectively investigated voxels that differed by the greatest amount, and often found individual subjects responsible: A single subject with a large observation can drive a conventional one‐sample *T*‐test, but when that same subject also has large intrasubject variance FSL's mixed effect model downweights that subject leading to a substantially different *T*‐test. The increased difference in AFNI's values for ds000109 for larger statistic values could also reflect a similar downweighting procedure within the software.

The Dice coefficients comparing the thresholded permutation test and parametric inferences are generally the best of any (Figure 4, 3‐element lower diagonal). In general the origin of parametric‐nonparametric differences are parametric inference finding a slightly larger number of clusters significant.

### 3.4 Conclusion

Across all three of the studies reanalysed here we have discovered considerable differences between the AFNI, FSL, and SPM results. The scale of these differences has been highlighted by each of the quantitative metrics applied to compare the group‐level statistic maps: Dice coefficients were commonly less than 50% for cross‐software comparisons, Bland–Altman plots showed that differences between reported *T*‐statistic values were as large as 4 for a considerable quantity of voxels, and EC curves displayed a divergence in the number of clusters being reported in each software—even at large thresholds.

In reporting these comparisons, we are not making any statements as to which software package is better or worse. Without a gold standard to compare against no such claims can be made, and we believe further development of well‐validated pipelines by multiple groups can encourage innovation and ultimately benefit the field. Rather, we feel that the key contribution of our work is the quantitative measurement of intersoftware differences on common datasets. Our finding that exceedingly weak effects may not generalise across packages—evidenced across all three of our analyses—is the primary take‐home message of this work. While larger effects were found to be more robust—demonstrated by the similar Neurosynth association analysis results that suggest some alignment in the final qualitative conclusions that can be drawn from all three software's statistical maps—we stress that our analyses have been conducted under particularly favourable conditions: The use of studies with a strong, primary effect and extensive efforts made to harmonise the three analyses. Because of this, at best our results present an optimistic view of intersoftware disparities. To better understand the underlying differences between software, further work on quantification of pipeline‐related variation is needed, which in the long‐term will hopefully lead to harmonisation in software implementation to reduce these differences in the future. Another line of work would be the creation of integrative intrastudy, ensemble learning techniques to integrate inconsistent findings. An additional contribution with this effort is to provide generalizable measures and metrics to enable software validation, which we hope may benefit any further comprehensive comparison of software packages.

## 4 DISCUSSION

Our results have displayed extensive differences in the results between the analysis pipelines of the three software packages. The low Dice values and differences in ECs (Figures 4 and 5) are particularly salient, showing heterogeneity in the sizes and shapes of clusters determined across packages, indicating a strong dependance on software in terms of the anatomical regions covered by the activation. While some authors have pointed out the limitations of interpreting differences in the set of voxels that make up a significant activation when using clusterwise inference (which makes assessments based on topological properties of statistical maps; Chumbley and Friston, 2009), we see merit in the use of quantitative measurements such as Dice due to the ultimate application of statistical maps to infer on precise areas of the brain active during a task. While a deeper analysis on the differences between topological aspects of images across software would be valuable, there are inherent difficulties in this approach, such as identifying corresponding features between maps when the number and size of activations reported are variable across software.

It is notable that the level of variation in our analysis results also fluctuated across the datasets we analysed. This is highlighted in our Dice comparisons, where the ds000001 Dice coefficients are considerably smaller than ds000109 for both the inter and intrasoftware comparisons. The relatively poor performance of ds000001 may be due to the smaller sample size for this study (16 vs. 21 for d000109), as well as the particular inference method used. For ds000001, group‐level inference was conducted using a cluster‐forming threshold of *p* < .01 uncorrected. A recent study (Eklund et al., 2016) found that parametric inference for a one‐sample *T*‐test at this threshold in AFNI, FSL, and SPM resulted in false‐positive rates far exceeding the nominal level—severely for cluster‐forming threshold *p* < .01, modestly for *p* < .001—while nonparametric permutation performed closer to the expected 5% FWE level. The results obtained here for ds000001 are consistent with these findings: across all three software packages, the thresholded images produced from permutation test inference display fewer significant clusters than the corresponding parametric maps. While the cluster‐defining threshold *p* < .005 applied in the ds000109 study was not analysed in Eklund et al., consistency between packages using parametric and nonparametric inference was greater for this study.

Notably, while all packages are purportedly using the same MNI atlas space, an appreciable amount of activation detected by AFNI and FSL fell outside of SPM's analysis mask (shown by the “spill over” values displayed in grey, Figure 4). Considerable differences in mask sizes are likely to have been a major factor for the disparities in activation and low dice coefficients seen across packages. For effects close to the edge of the brain, a larger analysis mask allows for a larger cluster volume, which can ultimately be the difference as to whether a cluster is determined as significantly active or not. This may explain why only FSL—which had the largest analysis mask—found an auditory response in the ds000109 study, or why Dice coefficients are generally worse for negative activations than positive in our ds000001 renanalyses, where positive clusters were on‐the‐whole reported in more central anatomical regions. Another possible reason for poor Dice values here is that the size and number of clusters determined for negative activations was smaller than that of positive activations. Since Dice and spill‐over values are proportional measurements, this means they will have been more susceptible to differences in cluster and mask size for the negative activations relative to the positive. Disagreement in atlas space may have contributed to the lack of structure in the Bland–Altman plots, however no gross misalignment between packages was evident (Figure S1). While far from perfect, the ds000120 AFNI and SPM thresholded results have the best Dice similarity score, likely due to the use of a very strong main effect as an outcome of interest.

Qualitative comparison of the results provide some optimism, with certain patterns of activation found across all packages. For example, the ds000001 parametric analyses were unanimous in determining significant activation in the anterior insula. While there is greater discordance over the precise location of activation within the anterior insular region, as well as the precise statistic values here, altogether our results align. This may substantiate that the strongest effects are robust across packages, supported by our own comparisons of the unthresholded maps that showed moderate agreement between software packages in areas with strong signal (many of these anatomical regions were identified across all three package's NeuroSynth association analyses) but greater disagreement elsewhere, ds000109 and ds000120 displaying more consistency than ds0000001. However, in making these qualitative comparisons, what has become most transparent is the importance for researchers to—at the very least—share their final statistical maps. The reasons for this are exhibited most clearly by our ds000109 analyses; the visual slice comparison of our replications of the main figure from the original study in Figure 1a, shown alongside the publication figure itself, look remarkably similar and could lead to the conclusion that each package's results highly agree. It is only when analysing these results over the whole brain, that we discover broad differences in these activation patterns, for example, positive activation identified in the auditory cortex in FSL that was not reported by AFNI and SPM, and significant deactivation determined only by AFNI and FSL.

At the start of our investigations, we selected a common set of preprocessing steps to be applied within each software package across all studies regardless of whether they had been used in the original analysis. This was to maximise the comparability of the results while being consistent with best practices within the community. However, several complications arose during our analyses. For ds000001, orientation information was missing from seven of the subject's structural and functional scans. Because the source DICOM files were no longer available, it was not possible to retrieve the original position matrices. As a consequence of this, the structural and functional images were misaligned, resulting in suboptimal coregistration during our analyses. Additionally, a bug in the event‐files induced during data conversion to the BIDS standard had resulted in some of the event timings being lost. Thanks to the cooperation of BIDS and OpenfMRI these problems were solved; a revised dataset (Revision: 2.0.4) was uploaded to OpenfMRI and used in our analysis.

Future efforts would be strengthened by additional sharing of analysis scripts and statistic maps, enabling confirmation of analyses that follow original procedures and permitting more quantitative comparison of statistic maps. We have made all of our analysis scripts available and statistic maps available, and we hope more researchers join this trend to advance openness in neuroimaging science.

### 4.1 Limitations

This study has mainly focused on comparing statistic maps, since these are the images studied to make judgments about localisation and determine the neuroscientific interpretation of results. However, by comparing the statistic maps obtained at the end of the pipeline, we have only assessed the net accumulation of differences across the entire analysis procedure. To illuminate the specific steps that contribute most to this variation, further in‐depth assessment of software differences at each stage of the analysis pipeline will be required. One recent example of this was a study that investigated differences in the prewhitening procedures conducted in AFNI, FSL, and SPM, by employing an analysis pipeline that used a single software package to carry out all other stages of processing (Olszowy et al., 2018). Further work could consider the factorial expansion of all possible combinations of preprocessing, first level modelling, and second level modelling, akin to previous efforts in assessing reproducibility over a number of pipelines (Strother et al., 2002).

Due to the restrictive requirements of this investigation—the necessity for published task‐based fMRI data using analysis methods compatible in AFNI, FSL, and SPM—the three studies analysed here were found to be the only datasets hosted on OpenfMRI suited to the aims of our investigation. Of the datasets that were not used, the most common reasons for exclusion were that no publication was associated to the data, that the sample size of the study was too small, or that custom software or region of interest analysis had been used as part of the analysis pipeline which was not feasible across the three software packages. Nevertheless, a greater sample of studies will need to be replicated across the packages to gain a more comprehensive understanding of the variability between software and validate the results found here. With increasing access to population neuroimaging studies, where thousands of fMRI subject data are available, a future study could test for nonzero software‐related variation by splitting a large dataset (e.g., UK Biobank [Alfaro‐Almagro et al., 2018], *N* > 10,000) into smaller subsets to generate an extensive collection of replication analyses across the three packages. This may allow for the creation of a null‐distribution from which differences between software results could be assessed in terms of statistical significance and confidence intervals, expanding on the raw concrete differences between *T*‐statistics maps highlighted in this effort. As simulation techniques become more advanced, there is also the potential for creation of synthetic subject‐level fMRI data as a ground truth to which each software package's results could be compared (Ellis et al., 2019).

Of the datasets we did use, subject data were missing from both the ds000109 and ds000120 datasets. For ds000109, while 29 young adults were scanned for the false belief task, only 21 were present in the dataset; for ds000120, we analysed 17 subjects instead of 30 used in the original study. These analyses therefore should not be compared like‐for‐like with the published results, and have substantially less statistical power than the original studies. Overall, our sample sizes for the three datasets analysed (ds000001, ds000109, ds000120) are 16, 21, and 17, respectively. While small, these sample sizes are fairly representative of a typical functional neuroimaging study over the past two decades—between 1995 and 2015, the median sample size of an fMRI study increased steadily from 8 to 22 (Poldrack et al., 2017). This increase has continued, and a review of 2017 publications found a median sample size of 33 (Yeung, 2018). Hence while our datasets are important for judging previous work, a future comparison exercise with larger datasets would be a valuable addition to the literature.

We have kept many parameters fixed in our analyses, such as the use of nonlinear registration for all software packages, and the addition of motion regressors in all our design matrices. How changes in these variables influence the analysis results warrants further investigation; for example, while we decided to fix a 2 mm cubic voxel size in all packages (since this is the default in FSL, SPM), a recent study found that alterations in this parameter can significantly impact statistical inference (Mueller et al., 2017). There are also many areas of the parameter space we have not explored, such as the inclusion of analyses that use small volume corrections, more stringent cluster‐forming thresholds (Eklund et al., 2016); (Woo et al., 2014), and two‐tailed testing (Chen et al., 2018).

Finally, the use of a wider range of software packages (e.g., FreeSurfer; [Dale et al., 1999]), as well as different software versions which were not accounted for in the present study would also strengthen any future analysis.

## DATA AVAILABILITY STATEMENT

The data that support the findings of this study are available in the NITRC Software Comparison Project ‐ Task fMRI repository at https://scp_taskfmri.projects.nitrc.org/. These data were derived from the following resources available in the public domain: OpenfMRI ds000001 dataset, https://www.openfmri.org/dataset/ds000001/; OpenfMRI ds000109 dataset, https://www.openfmri.org/dataset/ds000109/; OpenfMRI ds000120 dataset, https://www.openfmri.org/dataset/ds000120/.

## Supporting information


**Appendix S1.** Percentage BOLD change Maps
**Appendix S2.** Partial *R* ^2^ Maps

**Figure S1** Registration QC: Mean and standard deviation of anatomical and mean functional images
**Figure S2.** ds000001 Inter‐Software Comparison, 5% FWE Clusterwise Inference
**Figure S3.** ds000001 Inter‐Software Comparison, 5% FWE Clusterwise Permutation Inference
**Figure S4.** ds000109 Inter‐Software Comparison, 5% FWE Clusterwise Inference
**Figure S5.** ds000109 Inter‐Software Comparison, 5% FWE Clusterwise Permutation Inference
**Figure S6.** ds000120 Inter‐Software Comparison, 5% FWE Clusterwise Inference
**Figure S7.** ds000001 Inter‐Software Comparison, T‐Statistic Maps
**Figure S8.** ds000001 Inter‐Software Comparison, T‐Statistic Maps from Permutation
**Figure S9.** ds000109 Inter‐Software Comparison, T‐Statistic Maps
**Figure S10.** ds000109 Inter‐Software Comparison, T‐Statistic Maps from Permutation
**Figure S11.** ds120 Inter‐Software Comparison, F‐Statistic Maps
**Figure S12.** ds000120 Inter‐Software Comparison, Euler Characteristic and Cluster Count Curves for F‐Statistic Maps
**Figure S13.** Bland–Altman Percent BOLD Comparisons
**Figure S14.** ds000120 R^2^ Comparisons
