# Erratum: Exploring the impact of analysis software on task fMRI results

**DOI:** 10.1002/hbm.25302

**Published:** 2020-12-14

**Authors:** Alexander Bowring, Camille Maumet, Thomas E. Nichols

**Affiliations:** ^1^ Big Data Institute, Li Ka Shing Centre for Health Information and Discovery, Nuffield Department of Population Health University of Oxford Oxford UK; ^2^ Univ Rennes, Inria CNRS, Inserm Rennes France; ^3^ Wellcome Centre for Integrative Neuroimaging, FMRIB, Nuffield Department of Clinical Neurosciences University of Oxford Oxford UK; ^4^ Department of Statistics University of Warwick Coventry UK

In 2019 we published *Exploring the impact of analysis software on task fMRI results* in *Human Brain Mapping* (https://doi.org/10.1002/hbm.24603), showing how the choice of software package used for analysing task fMRI data can influence the final results of a study. We reanalysed data from three published task fMRI studies: Schonberg et al., 2012 (also referred to as “ds000001” in the article); Moran et al., 2012 (“ds000109”); Padmanabhan et al., 2011 (“ds000120”). For each study we reproduced the main group‐level effect from each publication using each of the three main fMRI software packages: AFNI, FSL, and SPM. We then applied a variety of quantitative and qualitative comparison methods to assess the similarity of the statistical maps between the three software packages.

From further work we are currently carrying out on this topic, we have recently become aware that five out of the 14 analysis results used in the article contained errors.

The first of these two results were our AFNI nonparametric reanalyses of the Schonberg et al., 2012 (ds000001) and Moran et al., 2012 (ds000109) datasets. In both cases, the wrong sub‐bricks of the 4D subject‐level results files had been specified in the group‐level permutation test model, meaning that permutation tests were carried out on subject‐level statistic images rather than subject‐level *beta* images as was intended.

A similar problem also was found for our AFNI parametric analysis of the Padmanabhan et al., 2011 (ds000120), where again, the subject‐level statistic images were wrongly entered into the group‐level mixed‐effects analysis rather than the intended beta images.

The final two results were our FSL parametric and nonparametric reanalyses of the Moran et al., 2012 (ds000109) dataset. In both cases, the linear model contrast had been incorrectly specified; the FSL parametric and permutation results in the article were for the False Photo Question vs False Belief Question contrast rather than the intended False Photo Story vs False Belief Story contrast used in the original publication.

We have now corrected and reanalysed these five sets of results, and updated all of the comparison figures in the article accordingly.

For the nonparametric AFNI analyses, the corrected results have led to only minor changes in the quantitative comparisons that were originally reported. Perhaps most notably, the within‐software Dice coefficients comparing the thresholded parametric and nonparametric results obtained within AFNI for both the ds000001 and ds000109 studies are now slightly *worse* in light of these new results. This is likely to be because the use of statistic images (instead of betas or contrasts) in the permutation group model mimicked a type of mixed effects inference more similar to the parametric mixed effects analysis. For ds000001, the AFNI parametric/nonparametric dice coefficient has decreased from 0.833 to 0.700, and for ds000109, the corresponding dice coefficient has decreased from 0.899 to 0.819.

A similar conclusion also holds for our corrected parametric ds000120 AFNI analysis results, which are now *less* similar to the corresponding set of results obtained in SPM compared to our original findings. Here, the AFNI‐SPM Dice coefficient has dropped from 0.684 to 0.545, and the AFNI‐SPM correlation coefficient for the threshold maps has dropped from 0.748 to 0.650.

For the parametric and nonparametric FSL ds000109 analyses, our corrected analyses have led to notable improvements in the ds000109 AFNI‐FSL and FSL‐SPM inter‐software comparisons, in the form of higher correlations between the unthresholded statistical maps and larger Dice coefficients for comparisons of the thresholded statistical maps. Specifically, correlations now range from 0.429 to 0.870 (was 0.429 to 0.747), and Dice coefficients range from 0 to 0.769 (was 0 to 0.684) for between‐software comparisons.

Importantly, the main conclusion from the article, that is, that weak effects may not generalise across fMRI software, has not changed in light of these corrections. Since finding these errors, we have investigated and verified all other models as correct and consistent across the three studies and software packages.

All slice views of the thresholded and unthresholded statistical maps (Figures 1 and 2), Bland Altman comparisons (Figures 3, 6 and 7), Dice similarity measures (Figure 4), Euler Characteristic and Cluster Counts (Figure 5), Neurosynth analyses (Table 2), and mean difference and correlation values (Table 3) involving the erroneous AFNI/FSL results are superseded by the amended figures/tables. New Neurovault repositories containing the corrected ds000001 statistical maps (https://neurovault.org/collections/8447/), corrected ds000109 statistical maps (https://neurovault.org/collections/7782/), and corrected ds000120 statistical maps (https://neurovault.org/collections/8468/) also replace the corresponding ds000001 (https://neurovault.org/collections/4110/), ds000109 (https://neurovault.org/collections/4099/), and ds000120 (https://neurovault.org/collections/4100/) repositories as given in the original article, and we have created a new release on github including all the code used for our corrected analyses (Release Software_Comparison_0.9.0, https://github.com/NISOx‐BDI/Software_Comparison/releases/tag/0.9.0). Derived data for the corrected analyses (and all other analyses) are available at https://scp_taskfmri.projects.nitrc.org/.

We now itemise corrections to the text in the article as published in *Human Brain Mapping* made in light of the corrected results:

**Abstract**

*p*.*3362 “However*, *we also discover marked differences*, *such as Dice similarity coefficients ranging from 0*.*000 to 0*.*684 in comparisons of thresholded statistic maps between software*.*”*

Should instead appear as:

*“However*, *we also discover marked differences*, *such as Dice similarity coefficients ranging from 0*.*000 to 0*.*769 in comparisons of thresholded statistic maps between software*.*”*

**3. Results**

*p*.*3373 “… group‐level statistic maps used to create the figures in this section are available on NeuroVault*: https://neurovault.org/collections/4110/, https://neurovault.org/collections/4099/, https://neurovault.org/collections/4100/, *for ds0000001*, *ds000109*, *and ds000120*, *respectively*.*”*

Should instead appear as:

*“… group‐level statistic maps used to create the figures in this section are available on NeuroVault*: https://neurovault.org/collections/8447/, https://neurovault.org/collections/7782/, https://neurovault.org/collections/8468/, *for ds0000001*, *ds000109*, *and ds000120*, *respectively*.*”*

**3.1 Cross‐software variability**

*p*.*3374 ‘The ds000109 study reported activations in the bilateral temporoparietal junction*. *(TPJ)*, *precuneus*, *anterior superior temporal sulcus (aSTS)*, *and dorsal medial prefrontal cortex (dmPFC)*. *Similar activations from our reanalyses are seen in* Figure 1(a), *middle*, *although FSL only found activation in the right TPJ and aSTS*. *Further comparisons shown in* Figure 1(b), *middle*, *highlight disagreement in the results*: *AFNI and FSL detected significant deactivations in distinct brain regions (inferior temporal gyrus for AFNI*, *inferior frontal gyrus for FSL)*, *while SPM did not determine any significant deactivation*. *FSL also found a positive response in the superior temporal gyrus (STG) where AFNI and SPM did not (*Figure 1(b), *middle*, *z = 0 and z = 12 slices)*.*’*

Should instead appear as:

*‘The ds000109 study reported activations in the bilateral temporoparietal junction*. *(TPJ)*, *precuneus*, *anterior superior temporal sulcus (aSTS)*, *and dorsal medial prefrontal cortex (dmPFC)*. *Similar activations from our reanalyses are seen in* Figure 1(a), *middle*, *however*, *further comparisons shown in* Figure 1(b), *middle*, *highlight some disagreement in the results*: *AFNI detected significant deactivations in the inferior temporal gyrus*, *while FSL and SPM did not determine any significant deactivation*. *AFNI and SPM also found a positive response in the cerebellum where FSL did not (*Figure 1(b), z *= −32 slices)*.*’*

*p*.*3375 “Pairwise correlations ranged from 0*.*429 to 0*.*747 for intersoftware comparisons (Table 3)*.*”*

Should instead appear as:

*“Pairwise correlations ranged from 0*.*429 to 0*.*870 for intersoftware comparisons (Table 3)*.*”*

*p*.*3376 “These values improve for ds000109*, *where the mean Dice coefficient for positive activations is 0*.*512*. *Here*, *AFNI and FSL were the only software packages to report significant negative clusters for the ds000109 study*. *Strikingly*, *these activations were found in completely different anatomical regions for each package*, *witnessed by the negative activation AFNI/FSL dice coefficient of 0*. *Finally*, *the AFNI/SPM Dice coefficient for the thresholded F‐statistic images obtained for ds000120 is 0*.*684*; *it is notable that across all studies*, *the AFNI/SPM dice coefficients are consistently the largest*.*”*

Should instead appear as:

*“These values improve considerably for ds000109*, *where the mean Dice coefficient for positive activations is 0*.*707*. *Since AFNI was the only software package to report any significant negative clusters for the ds000109 study*, *the AFNI/FSL and AFNI/SPM dice coefficient for negative activations is effectively 0 here*. *Finally*, *the AFNI/SPM Dice coefficient for the thresholded F‐statistic images obtained for ds000120 is 0*.*684*.*”*

**3.2 Cross‐software variability for nonparametric inference**

*p*.*3378 ‘Quantitative assessment with Dice coefficients are shown in* Figure 4 *(“perm”* vs. *“perm” cells) and—in accordance with the parametric results—are generally poor*. *Like the parametric analyses*, *AFNI/SPM Dice values are altogether better than the other comparisons*.*’*

Should instead appear as:

*‘Quantitative assessment with Dice coefficients are shown in* Figure 4 *(“perm”* vs. *“perm” cells) and are on‐the‐whole similar to the corresponding parametric Dice comparisons*.*’*

*p*.*3379 ‘Notably*, *while AFNI and SPMs EC curves are relatively similar across choice of inference method*, *FSL permutation inference determined substantially more clusters than parametric for low positive thresholds in both studies (*Figure 5(a),(b), *bottom)*.*’*

Should instead appear as:

‘*Notably*, *while AFNI and SPMs EC curves are relatively similar across choice of inference method*, *FSL permutation inference determined substantially more clusters than parametric at low positive thresholds for ds000001(*Figure 5(a),(b), *bottom left)*.*’*

**3.3 Intra‐software variability, parametric vs nonparametric**

*p*.*3380 ‘Bland–Altman plots (*Figure 7 *) reveal much greater levels of parametric‐nonparametric agreement*, *with AFNI displaying greater agreement than FSL*.*’*

Should instead appear as:

*‘Bland–Altman plots (*Figure 7 *) reveal much greater levels of parametric‐nonparametric agreement relative to the corresponding plots for between‐software comparisons (*Figure 3 and Figure 6 *)*.*’*

*p*.*3380 “The increased difference in AFNI’s values for ds000109 for larger statistic values could also reflect a similar downweighting procedure within the software*.*”*

Should instead appear as:

*p*.*3380 “The same parametric‐nonparametric differences seen in the AFNI BA plots for larger statistic values may also reflect a similar downweighting procedure used for parametric inference within the software*.*”*

**4. Discussion**

*p*.*3381 “This may explain why only FSL—which had the largest analysis mask—found an auditory response in the ds000109 study*, *or why Dice coefficients are generally worse for negative activations than positive in our ds000001 renanalyses*, *where positive clusters were on‐the‐whole reported in more central anatomical regions*.*”*

Should instead appear as:

*“This may explain why Dice coefficients are generally worse for negative activations than positive in both the ds000001 and ds000109 renanalyses*, *where positive clusters were on‐the‐whole reported in more central anatomical regions*.*”*

*p*.*3381 “While far from perfect*, *the ds000120 AFNI and SPM thresholded results have the best Dice similarity score*, *likely due to the use of a very strong main effect as an outcome of interest”*.

Should be removed.

*p*.*3381 “It is only when analysing these results over the whole brain*, *that we discover broad differences in these activation patterns*, *for example*, *positive activation identified in the auditory cortex in FSL that was not reported by AFNI and SPM*, *and significant deactivation determined only by AFNI and FSL*.*”*

Should instead appear as:

*“It is only when analysing these results over the whole brain*, *that we discover broad differences in these activation patterns*, *for example*, *positive activation identified in the cerebellum in AFNI and SPM that was not reported by FSL*, *and significant deactivation determined only by AFNI*.*”*

**Figure 4 Caption**

*p*.*3376 “For ds000001 increases*, *FSL permutation obtained no significant results*, *thus generating Dice coefficients of zero Dice coefficients of zero*; *for ds000109 decreases*, *only AFNI and FSL parametric obtained a result and hence only one coefficient is displayed*. *Dice coefficients are mostly below 0*.*5*, *parametric‐nonparametric intrasoftware results are generally higher*; *ds000120”s F‐statistic results are notably high*, *at 0*.*684*, *perhaps because it is testing a main effect with ample power’*.

Should instead appear as:

“*For ds000001 increases*, *FSL permutation obtained no significant results*, *thus generating Dice coefficients of zero Dice coefficients of zero*; *for ds000109 decreases*, *no comparisons are shown as only AFNI parametric obtained a result*. *However*, *this effectively means the AFNI/FSL and AFNI/SPM Dice coefficient is also zero here*. *Dice coefficients range between 0 and 0*.*75*, *and are commonly below 0*.*5 for comparisons between software packages*. *Parametric‐nonparametric intrasoftware comparisons are higher*, *with most of the dice coefficients above 0*.*8*.*”*

**FIGURE 1:**
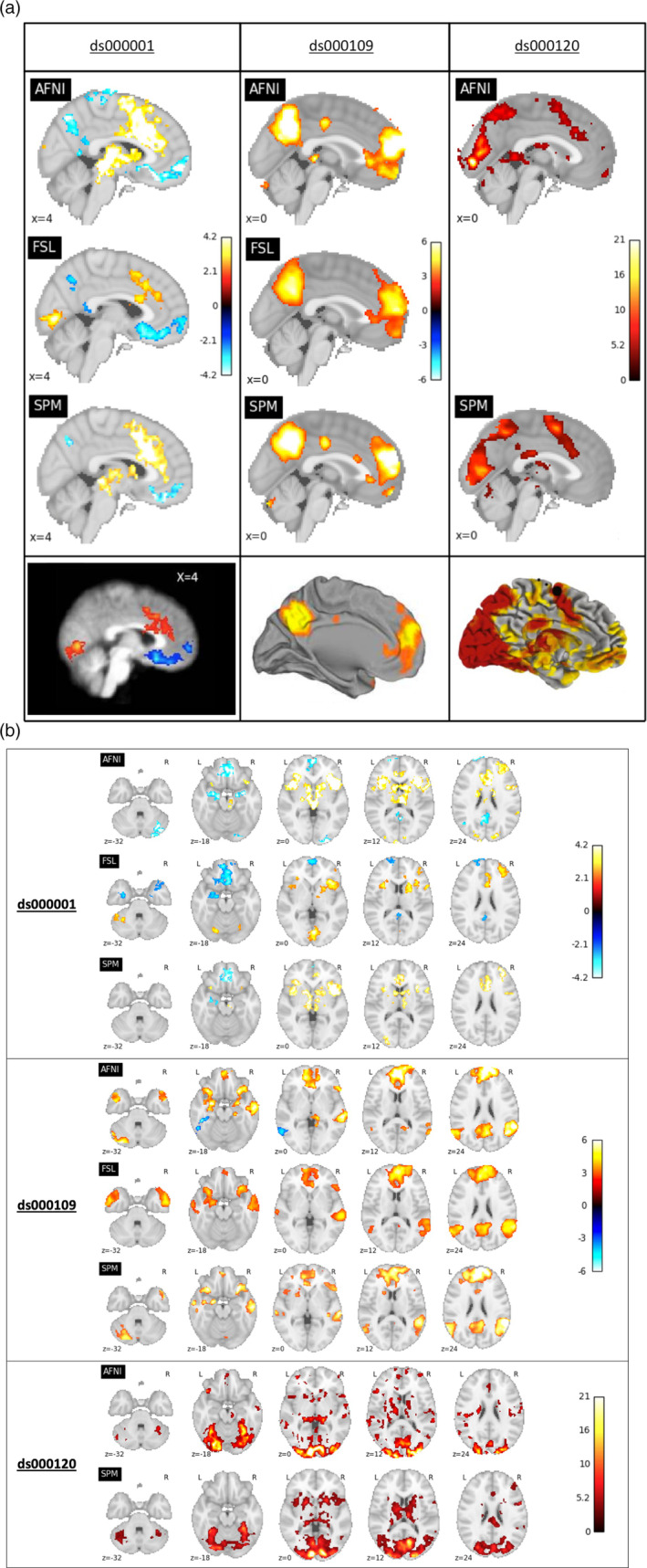
(a) Comparison of the thresholded statistic maps from our reanalysis with the main figures from each of the three publications. Left: For ds000001 data, thresholded T‐statistic images contrasting the parametric modulation of pumps of reward balloons versus the parametric modulation of the control balloon; beneath, a sagittal slice taken from Figure 3 in Schonberg et al. (2012). Middle: For ds000109, thresholded T‐statistic maps of the false belief versus false photo contrast; beneath, a midsagittal render from Moran et al. (2012). Right: For ds000120, thresholded F‐statistic images of the main effect of time contrast; beneath, a midsagittal render from Figure 3 in Padmanabhan et al. (2011). Note that for ds000109 and ds000120 the publication’s figures are renderings onto the cortical surface while our results are slice views. While each major activation area found in the original study exists in the reanalyses, there is substantial variation between each reanalysis. (b) Comparison of the thresholded statistic maps from our reanalysis displayed as a series of axial slices. Top: ds000001's thresholded T‐statistic maps contrasting parametric modulations of the reward balloons versus pumps of the control balloons. Middle: ds000109's thresholded T‐statistic maps of the false belief versus false photo contrast. Bottom: ds000120's thresholded F‐statistic maps of the main effect of time contrast. This figure complements the single slice views shown in Figure 1(a)

**FIGURE 2:**
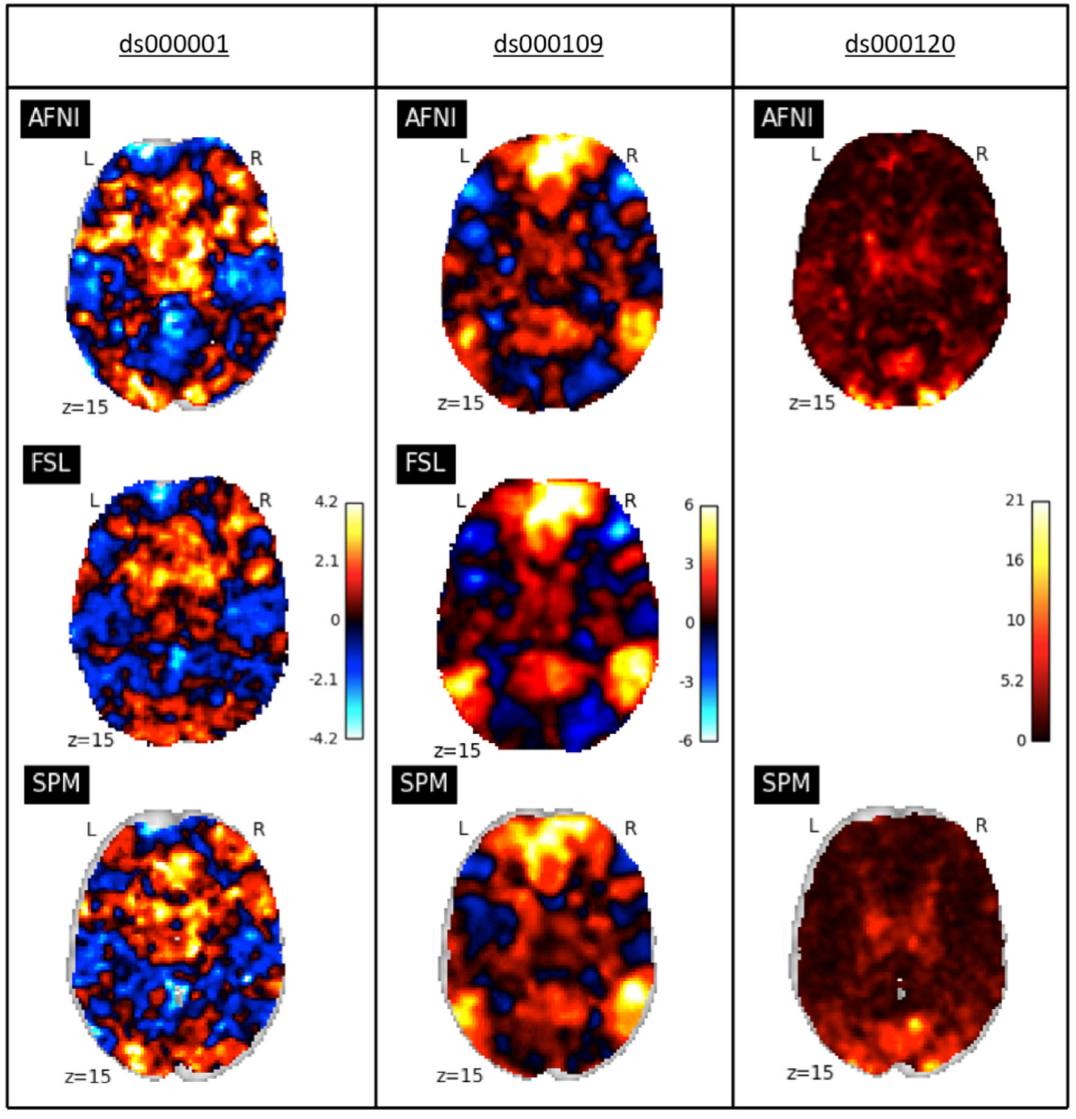
Comparison of the unthresholded statistic maps from our reanalysis of the three studies within each software package. Left: ds000001's unthresholded T‐statistic maps of the parametric modulation of pumps of reward balloons versus the parametric modulation of the control balloon contrast. Middle: ds000109's unthresholded T‐statistic maps of the false belief versus false photo contrast. Right: ds000120's unthresholded F‐statistic maps of the main effect of time contrast. While areas of strong activation are somewhat consistent across all three sets of reanalyses, there is substantial variation in nonextreme values

**FIGURE 3:**
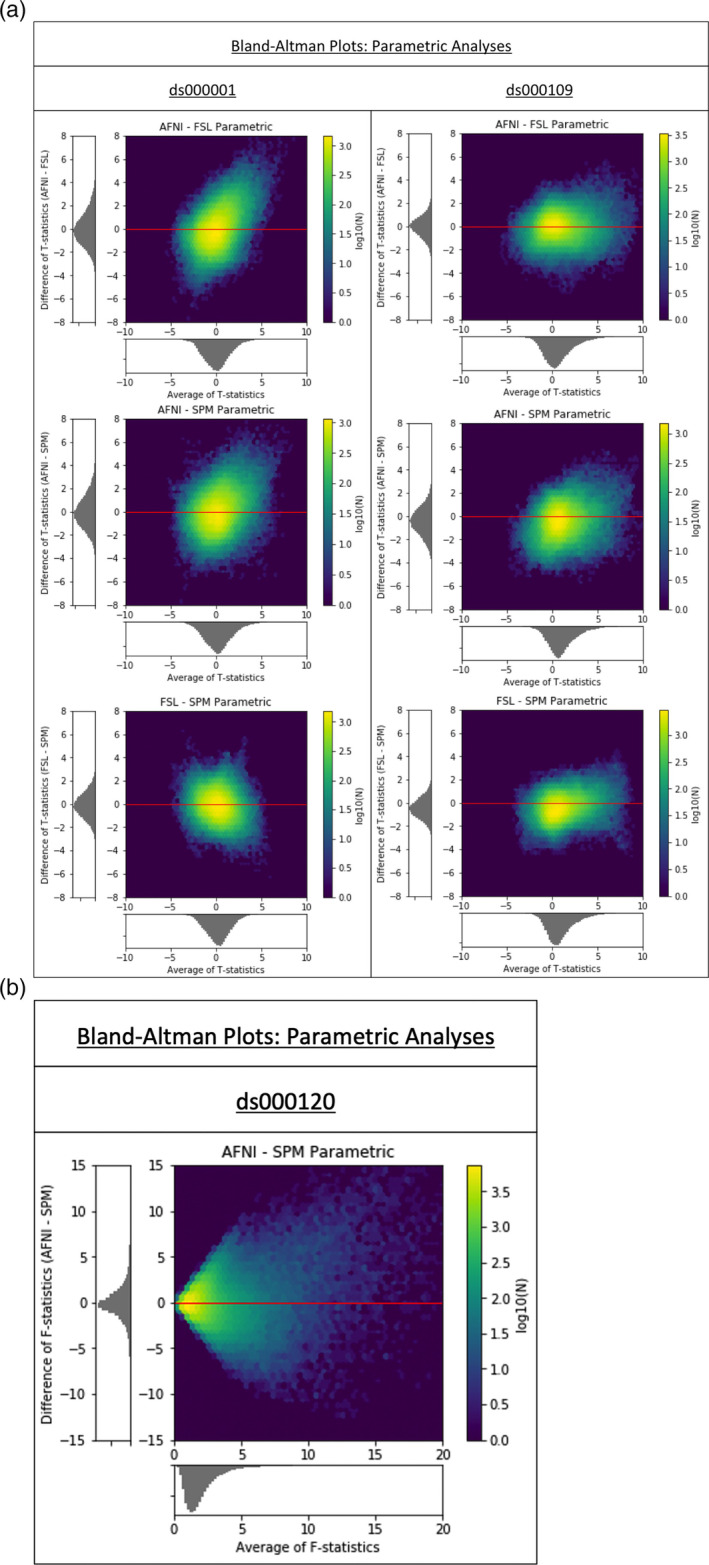
(a) Cross‐software Bland–Altman 2D histograms comparing the unthresholded group‐level T‐statistic maps computed as part our reanalyses of the ds000001 and ds000109 studies within AFNI, FSL, and SPM. Left; Comparisons for ds000001's balloon analog risk task, T‐statistic images contrasting the parametric modulation of pumps of the reward balloons versus parametric modulation of pumps of the control balloon. Right; Comparisons for ds000109's false belief task, T‐statistic images contrasting the false belief versus false photo conditions. Density images show the relationship between the average T‐statistic value (abscissa) and difference of T‐statistic values (ordinate) at corresponding voxels in the unthresholded T‐statistic images for each pairwise combination of software packages. While there is no particular pattern of bias, as the T‐statistic differences are centered about zero, there is remarkable range, with differences exceeding ±4 in all comparisons. (b) Cross‐software Bland‐Altman 2D histogram comparing the unthresholded main effect of time F‐statistic maps computed in AFNI and SPM for reanalyses of the ds000120 study. The differences are generally centered about zero, with a trend of large F‐statistics for AFNI. (The funnel ‐like pattern is a consequence of the F‐statistic taking on only positive values)

**FIGURE 4:**
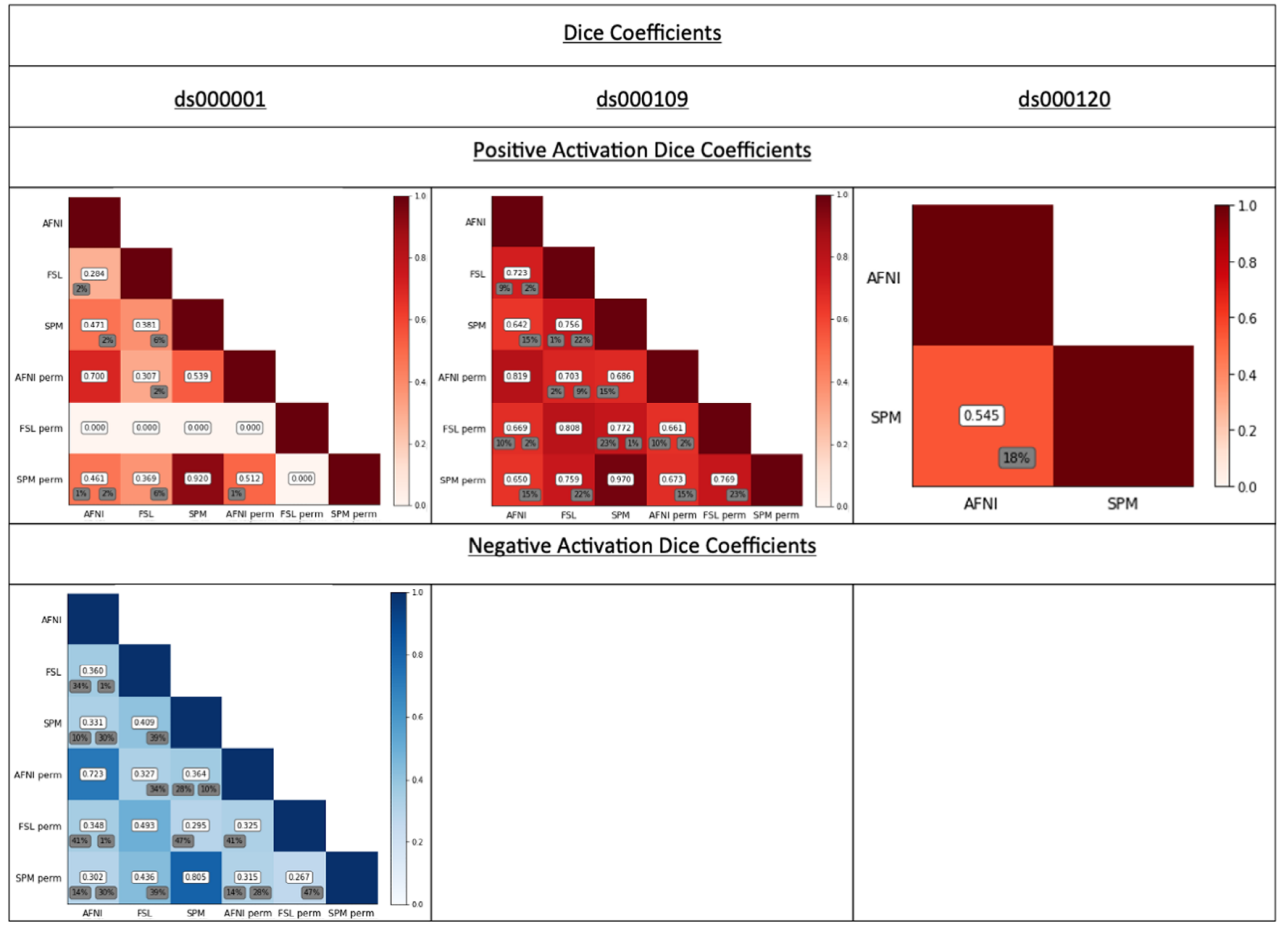
Dice coefficients comparing the thresholded positive and negative T‐statistic maps computed for each pair of software package and inference method for each of the three reproduced studies. Dice coefficients were computed over the intersection of the pair of analysis masks, to assess only regions where activation could occur in both packages. Percentage of “spill over” activation, that is, the percentage of activation in one software’s thresholded statistic map that fell outside of the analysis mask of the other software is displayed in grey; left value for row software, right value for column software. For ds000001 increases, FSL permutation obtained no significant results, thus generating Dice coefficients of zero Dice coefficients of zero; for ds000109 decreases, no comparisons are shown as only AFNI parametric obtained a result. However, this effectively means the AFNI/FSL and AFNI/SPM Dice coefficient is also zero here. Dice coefficients range between 0 and 0.75, and are commonly below 0.5 for comparisons between software packages. Parametric‐nonparametric intrasoftware comparisons are higher, with most of the dice coefficients above 0.8

**FIGURE 5:**
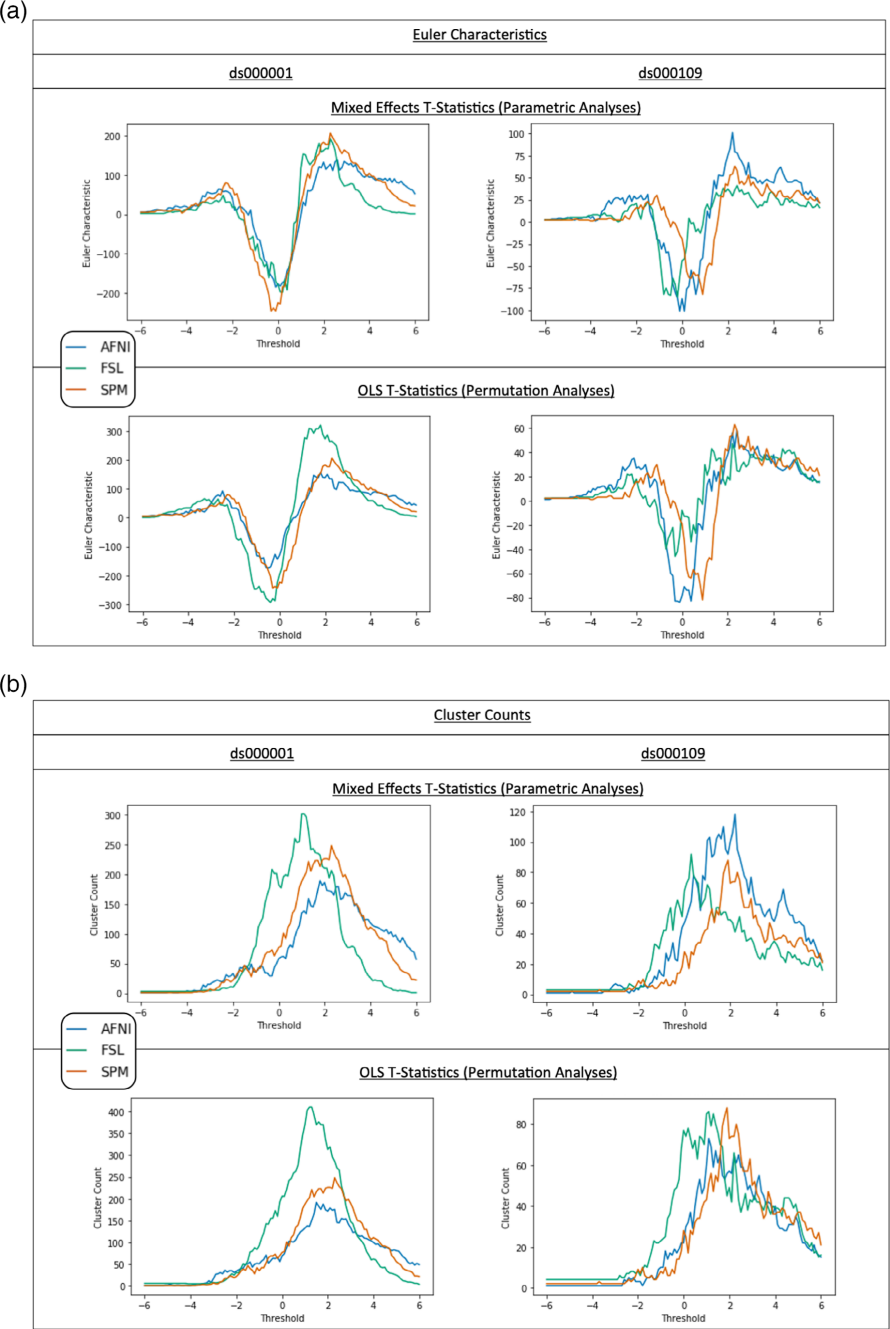
(a) Euler characteristic (EC) plots for ds000001 and ds000109. On top, comparisons of the Euler characteristic computed for each software’s T‐statistic map from our reanalyses using a range of T‐value thresholds between −6 and 6. Below, comparisons of the ECs calculated using the same thresholds on the corresponding T‐statistic images for permutation inference within each package. For each T‐value the EC summarises the topology of the thresholded image, and the curves provide a signature of the structure of the entire image. For extreme thresholds the EC approximates the number of clusters, allowing a simple interpretation of the curves: For example, for ds000001 parametric analyses, FSL clearly has the fewest clusters for positive thresholds. (b) Cluster count plots for ds000001 and ds000109. On top, comparisons of the number of cluster found in each software’s T‐statistic map from our reanalyses using a range of T‐value thresholds between −6 and 6. Below, comparisons of the cluster counts calculated using the same thresholds on the corresponding T‐statistic images for permutation inference within each package

**FIGURE 6:**
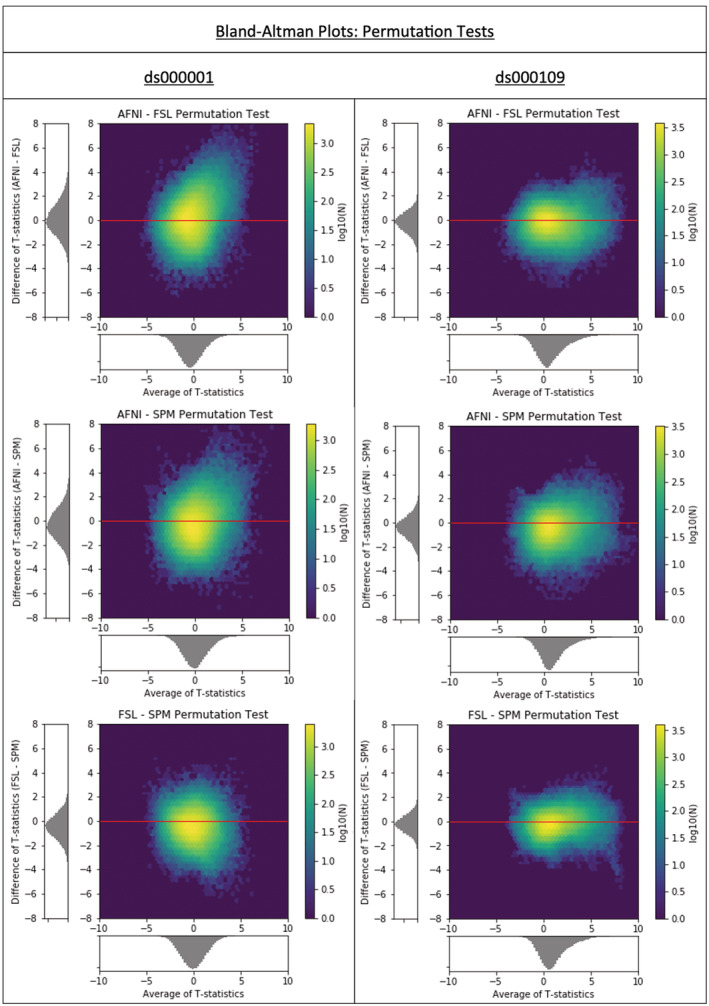
Cross ‐software Bland–Altman 2D histograms for the ds000001 and ds000109 studies comparing the unthresholded group‐level T‐statistic maps computed using permutation inference methods within AFNI, FSL, and SPM. Similar to the results obtained using parametric inferences in Figure 3, all of the densities indicate large differences in the size of activations determined within each package

**FIGURE 7:**
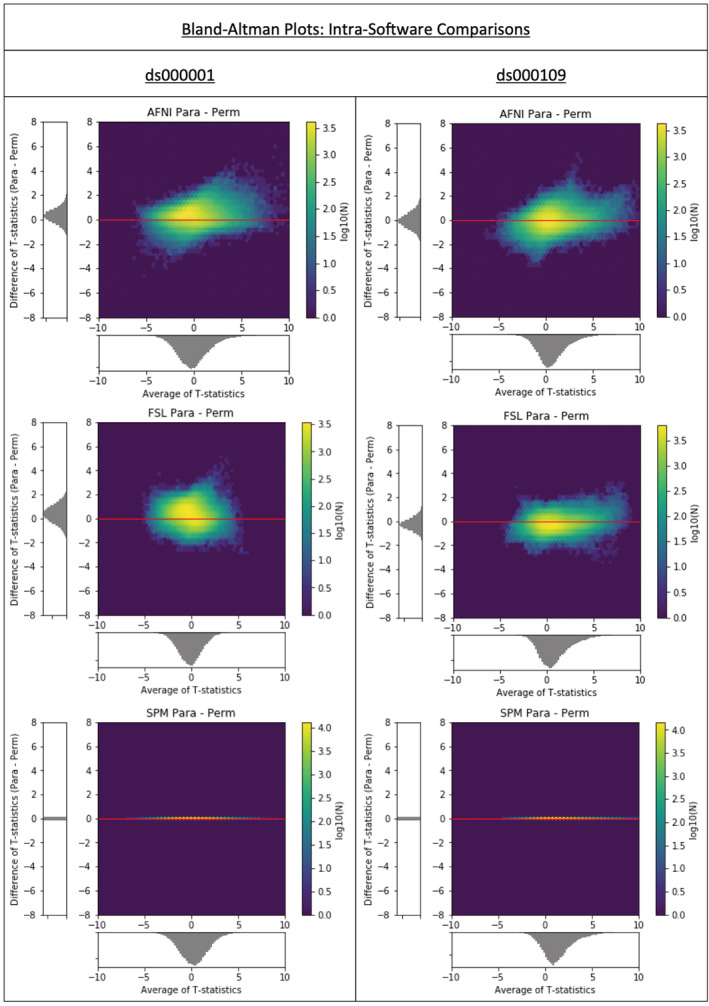
Intrasoftware Bland–Altman 2D histograms for the ds000001 and ds000109 studies comparing the unthresholded group‐level T‐statistic maps computed for parametric and nonparametric inference methods in AFNI, FSL and SPM. Each comparison here uses the same preprocessed data, varying only the second level statistical model. SPM’s parametric and nonparametric both use the same (unweighted) one‐sample T‐test, and thus show no differences. AFNI and FSL’s parametric models use iterative estimation of between‐subject variance and weighted least squares and thus show some differences, but still smaller than between‐software comparisons

**TABLE 2:** Neurosynth analyses

	AFNI		FSL		SPM	
	Neurosynth analysis	Corr.	Neurosynth analysis	Corr.	Neurosynth analysis	Corr.
**ds000001**	Anterior insula	0.359	Anterior insula	0.240	Anterior insula	0.322
	Insula	0.276	**Task**	0.233	Anterior	0.245
	Anterior	0.243	**Tasks**	0.203	Insula	0.240
	Insula anterior	0.233	Parietal	0.190	**Goal**	0.229
	Thalamus	0.221	**Goal**	0.188	**Task**	0.225
	**Goal**	0.211	**Working memory**	0.184	Insula anterior	0.214
	**Pain**	0.198	**Working**	0.181	Thalamus	0.201
	Supplementary	0.197	Basal ganglia	0.173	Acc	0.199
	Premotor	0.196	Ganglia	0.172	Anterior cingulate	0.196
	Anterior cingulate	0.192	Basal	0.169	Ganglia	0.188
**ds000109**	Medial prefrontal	0.422	Medial prefrontal	0.440	Medial prefrontal	0.361
	Medial	0.381	**Theory mind**	0.405	**Theory mind**	0.331
	**Default**	0.366	Medial	0.378	**Default**	0.329
	**Theory mind**	0.348	**Default**	0.377	Precuneus	0.314
	**Default mode**	0.341	**Mind**	0.370	**Default mode**	0.310
	Precuneus	0.334	**Social**	0.367	Medial	0.301
	Posterior cingulate	0.327	**Default mode**	0.356	**Mind**	0.296
	**Social**	0.322	**Mental states**	0.341	Prefrontal	0.294
	**Mind**	0.311	**Mind tom**	0.339	**Mind tom**	0.289
	**Mind tom**	0.287	**Tom**	0.331	Posterior cingulate	0.287
**ds000120**	**Visual**	0.386			**Visual**	0.481
	Occipital	0.358			Occipital	0.367
	v1	0.300			v1	0.340
	Early visual	0.276			Visual cortex	0.267
	Visual cortex	0.229			**Spatial**	0.248
	Occipito	0.221			Spl	0.245
	Fusiform	0.213			**Eye**	0.242
	Occipital cortex	0.190			Early visual	0.238
	Primary visual	0.189			Lingual	0.238
	Occipito temporal	0.177			Intraparietal	0.237

*Note*: The Neurosynth analysis terms most strongly associated (via Pearson Correlation) to each software’s group‐level statistic map across the three studies. Non‐anatomical terms are shown in bold.

**TABLE 3:** Summary of test statistics

		ds000001		ds000109		ds000120	
		Mean diff	Corr	Mean diff	Corr	Mean diff	Corr
**AFNI vs. FSL**	Parametric	0.009	0.616	−0.047	0.858		
	Non‐parametric	0.110	0.579	−0.231	0.857		
**AFNI vs. SPM**	Parametric	0.061	0.614	−0.490	0.747	−0.046	0.650
	Non‐parametric	−0.280	0.620	−0.450	0.802	n/a	n/a
**FSL vs. SPM**	Parametric	−0.047	0.684	−0.452	0.870		
	Non‐parametric	−0.479	0.720	−0.222	0.896		
**AFNI**	Para. Vs. NonP.	0.314	0.934	−0.041	0.924		
**FSL**	Para. Vs. NonP.	0.382	0.844	−0.226	0.955		
**SPM**	Para. Vs. NonP.	0.000	1.000	0.000	1.000		

*Note*: Mean differences and correlations for each pair of test statistic images; mean differences correspond to the y‐axes of the Bland Altman plots displayed in Figures 3(a), (b), & 7. Each mean difference is the first item minus second; for example, AFNI vs. FSL mean difference is AFNI‐FSL. Correlation is the Pearson’s r between the test statistic values for the pair compared. Inter‐software differences are greater than intra‐software.

 

## Supporting information


**Appendix** **S1**. Supporting Figures

## Data Availability

N/A

