# Supplementary material for: Exploring the impact of analysis software on task fMRI results
Source: Hum Brain Mapp. 2019 May 2;40(11):3362–84. doi: 10.1002/hbm.24603 (PMC6618324; doi:10.1002/hbm.24603)
Supplement: Supplementary file 1 — Appendix S1. Percentage BOLD change Maps Appendix S2. Partial R 2 Maps [file HBM-40-3362-s001.pdf]

Appendix

Appendix 1. Percentage BOLD change Maps

While each of the three software packages provide contrast of parameter estimate maps (going forth "contrast estimate"), the units of the analysis differs between software. We first review the issue of units for fMRI then how we addressed this for each software.

Raw fMRI data has arbitrary units, and a normalization step is required to produce effect estimates that are both comparable across subjects and give an interpretable magnitude of the BOLD effect. The final units of the contrast estimates depend on how the data, design matrix and contrast vectors are scaled.

Consider arbitrary first level data at a voxel $\boldsymbol{Y}$ (N-vector), fMRI design matrix $\boldsymbol{X}$ ($N \times P$ matrix), related as $\mathrm{E}(\boldsymbol{Y}) = \boldsymbol{X}\boldsymbol{\beta}$, where $\boldsymbol{\beta}$ (P-vector) are the regression coefficients and the effect of interest is $\boldsymbol{c}\hat{\boldsymbol{\beta}}$ where $\boldsymbol{c}$ (P-row-vector) is the contrast. The following scaling is needed to ensure interpretable contrasts of parameter estimates.

- **Data**: The data needs to be scaled so that 1 unit change corresponds to 1%,

$$\boldsymbol{Y}^* = \frac{100}{B}\boldsymbol{Y} \tag{1}$$

  where B is an estimate of the mean or baseline.

- **Design**: The design needs to be scaled such that a unit change in a coefficewnt gives rise to a unit effect change in the fitted data,

$$\boldsymbol{X}^* = \frac{1}{h}\boldsymbol{X} \tag{2}$$

  where h is the (assumed common) predictor baseline-to-peak (or baseline-to-plateau for long blocks).

- **Contrast**: The contrast needs to be scaled to preserve the units of the coefficients,

$$\boldsymbol{c}^* = \frac{1}{s}\boldsymbol{c}, \tag{3}$$

  where s is the sum of the positive contrast elements (or, if all less than zero, minus the sum of the negative elements).

The ideally scaled data, design and contrast gives rise to contrast estimate $\boldsymbol{c}^*\hat{\boldsymbol{\beta}}^*$ which we can relate to the arbitrarily scaled data:

$$\begin{aligned}
\boldsymbol{c}^*\hat{\boldsymbol{\beta}}^* &= \boldsymbol{c}^*(\boldsymbol{X}^{*\top}\boldsymbol{X}^*)^{-1}\boldsymbol{X}^{*\top}\boldsymbol{Y}^* && (4)\\
&= \frac{100h}{Bp}\boldsymbol{c}(\boldsymbol{X}^\top\boldsymbol{X})^{-1}\boldsymbol{X}^\top\boldsymbol{Y} && (5)\\
&= \frac{100h}{Bp}\boldsymbol{c}\hat{\boldsymbol{\beta}}. && (6)
\end{aligned}$$

This scaling is described in terms of first level models, but can be applied to a 2nd level contrast estimate if the values of h, B and p are the same for all subjects.

In AFNI, a localized (i.e. voxelwise) approach is taken to overcome this hurdle (Chen, Taylor, and Cox, 2017). By including the 'scale' block in our subject-level analysis scripts, each voxel's time series was normalized (in our notation, using a voxel-wise B) to a voxelwise mean of 100 for all subjects. Scaling of the design matrix is conducted implicitly within AFNI, and as the sum of the contrast elements was 1 in all our analyses, no contrast scaling was necessary. Because of this, the effect estimate maps obtained at the group-level in our AFNI analyses could be directly interpreted as percentage BOLD change maps.

In FSL and SPM a global (i.e. brain-wide) approach is taken for data scaling, so that the modelled data ($\boldsymbol{Y}$) has a typical mean value of $B = 100$ for SPM, and $B = 10,000$ for FSL. In practise, it is known that SPM can underestimate the global mean intensities of each subject, leading to a grand mean intensity *larger* than 100 (Nichols, 2012). To account for this, in SPM we calculated B empirically by computing the mean image of all subject-level functional maps and then finding the median value over subjects. We computed h directly for the block design (ds000109) and based on an isolated event for the event-related design (ds00001); in FSL this required creating dummy designs with high-pass filtering disabled. Contrast scaling was only required in SPM ($s = 3$ for ds000001's 3-run design, $s = 2$ for ds000109's 2-run design).

Appendix 2. Partial R^2 Maps

Since ds000120 used a sine basis HRF, this study's general linear model contained multiple predictor variables which hampered the computation of a percent BOLD change measure. Instead we computed partial R^2 maps to assess the explained variance (relative to data variance not already explained by other terms) of the main effect of the saccade condition.

With ν_1 and ν_2 as the numerator and denominator degrees of freedom of the F-statistic, respectively, we used the relationship between R^2 and the F-statistic given by the identity:

$$F = \frac{R^2}{1 - R^2} \frac{\nu_2}{\nu_1}, \tag{7}$$

which solves for R^2 as

$$R^2 = 1 - \frac{1}{1 + \frac{\nu_1}{\nu_2} F}. \tag{8}$$

References

Chen, G., P.A. Taylor, and R.W. Cox (2017). "Is the statistic value all we should care about in neuroimaging?" In: *Neuroimage* 147, pp. 952–959. DOI: 10.1016/j.neuroimage.2016.09.066.

Nichols, Thomas (2012). *SPM plot units.* Accessed on 5 September 2018. URL: https://blog.nisox.org/2012/07/31/spm-plot-units.
