# Supplementary material for: Exploring the impact of analysis software on task fMRI results
Source: Hum Brain Mapp. 2019 May 2;40(11):3362–84. doi: 10.1002/hbm.24603 (PMC6618324; doi:10.1002/hbm.24603)

Supplementary Figures

Figure S1. Registration QC: Mean and standard deviation of anatomical and mean functional images

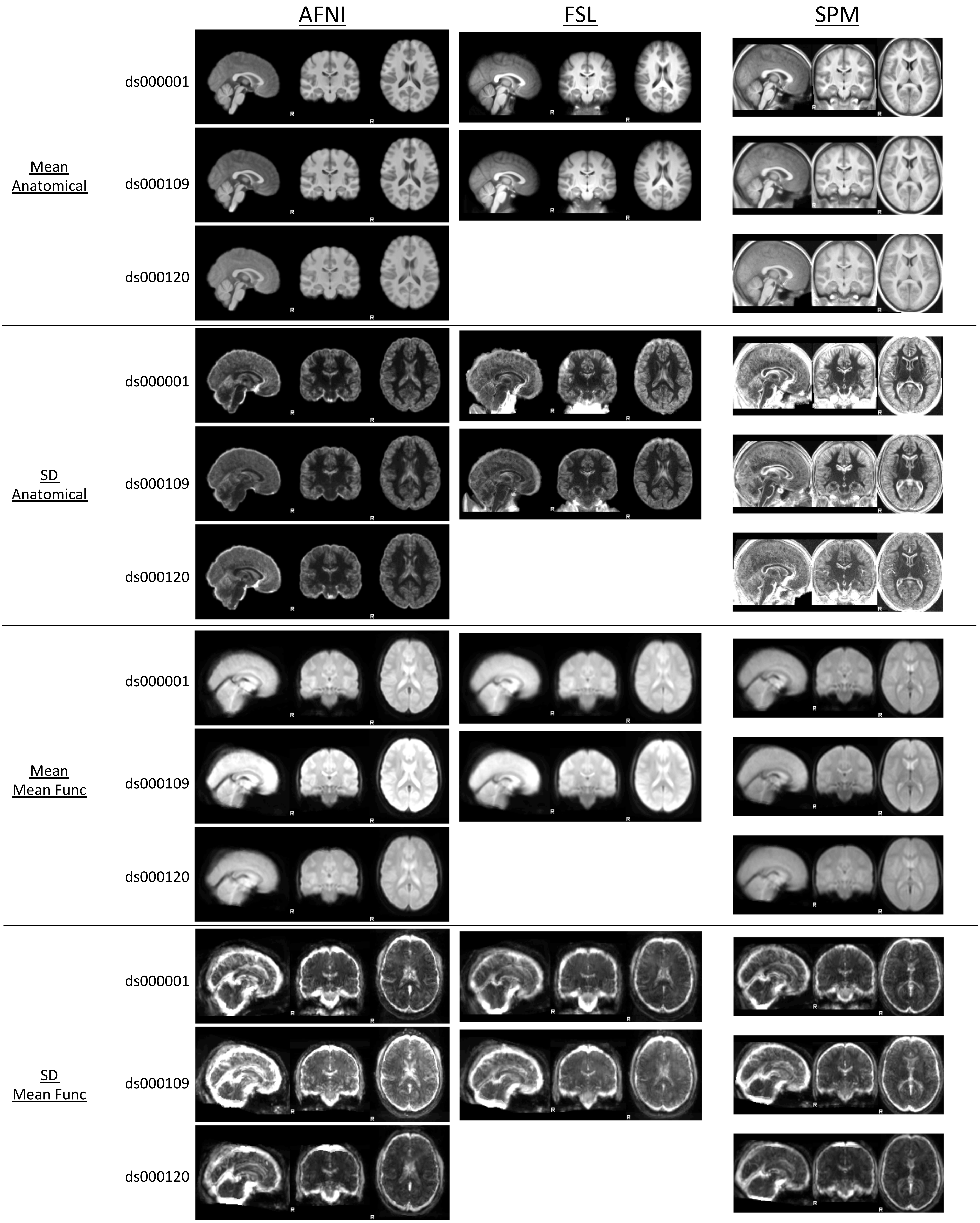

Figure S2. ds000001 Inter-Software Comparison, 5% FWE Clusterwise Inference

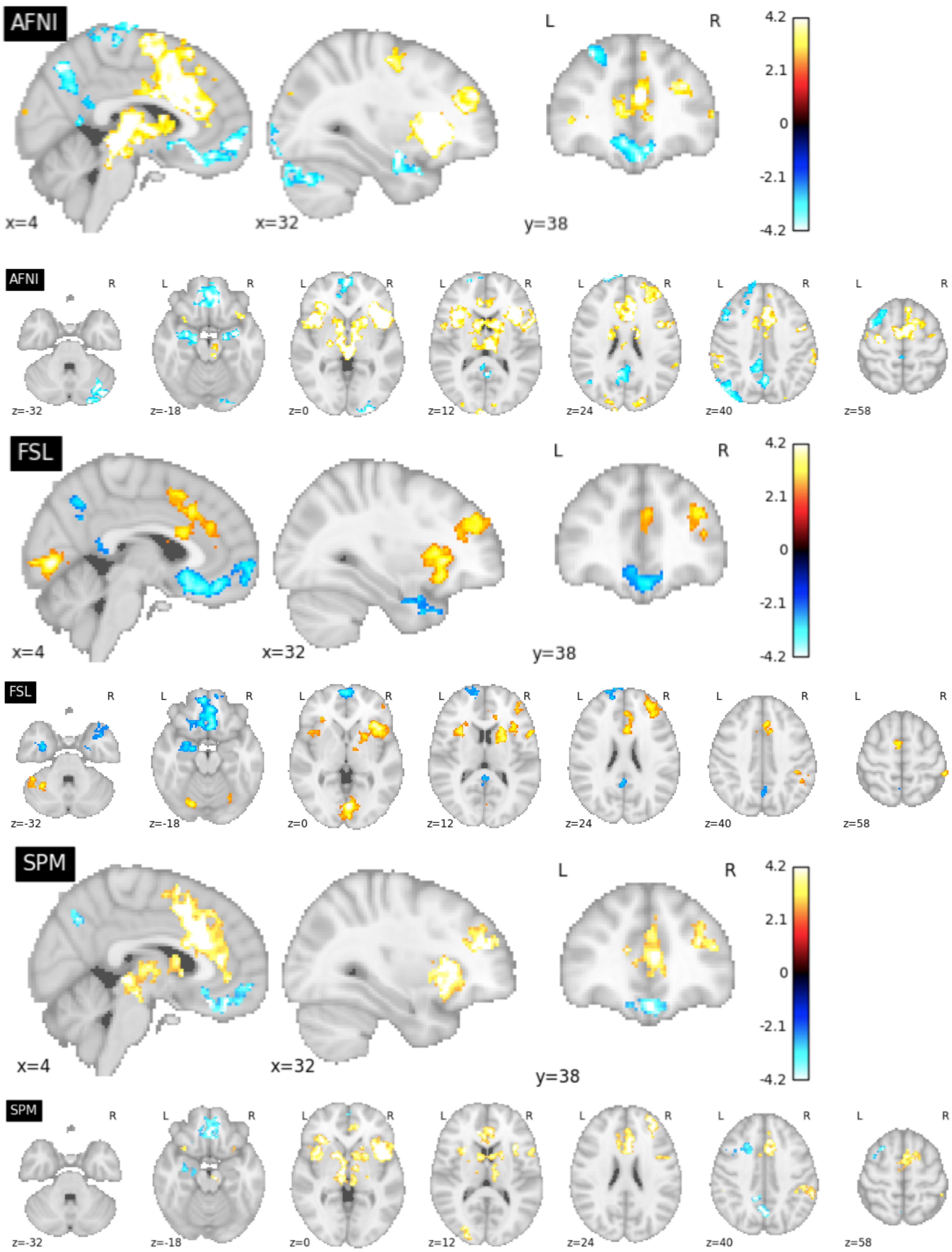

Figure S3. ds000001 Inter-Software Comparison, 5% FWE Clusterwise Permutation Inference

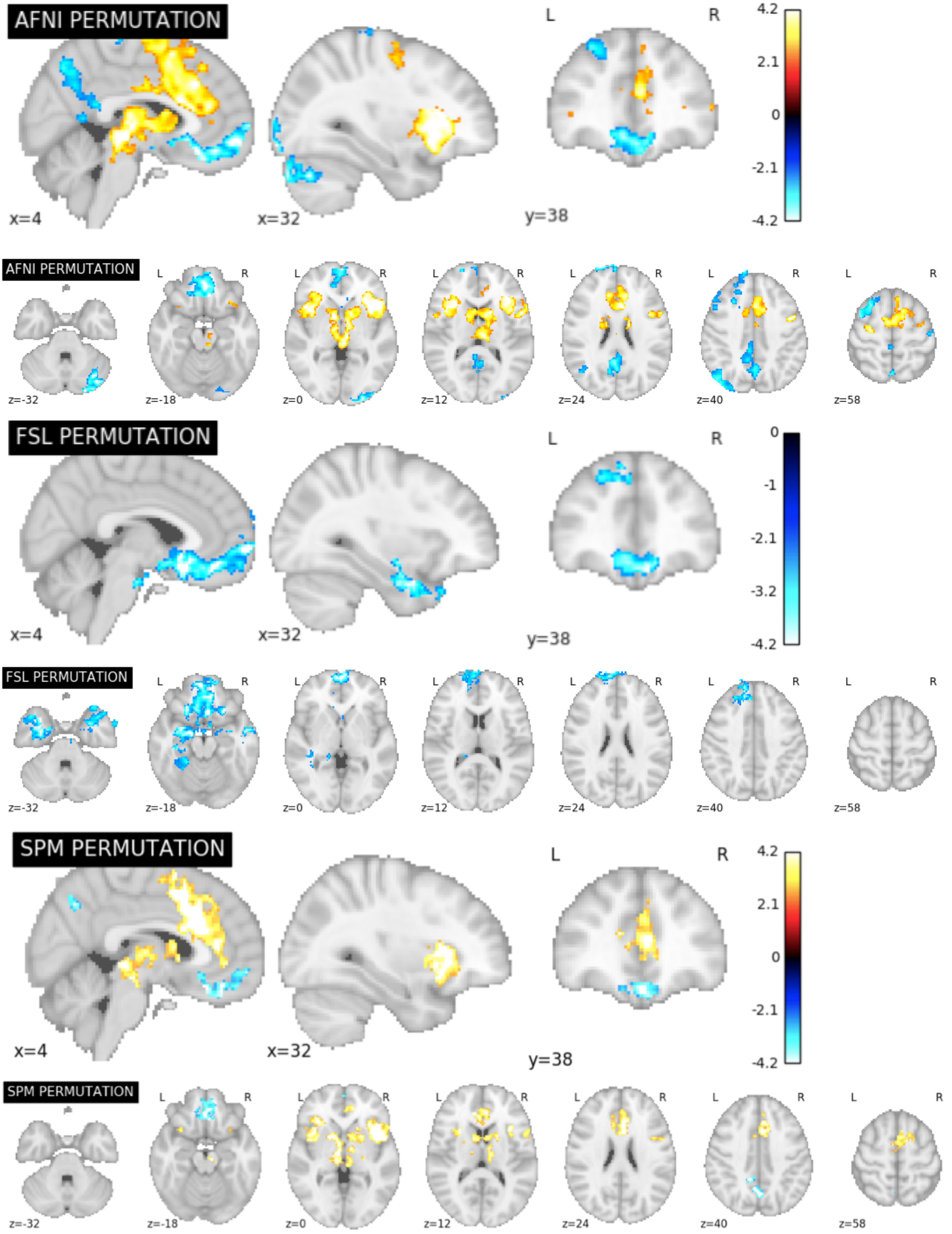

Figure S4. ds000109 Inter-Software Comparison, 5% FWE Clusterwise Inference

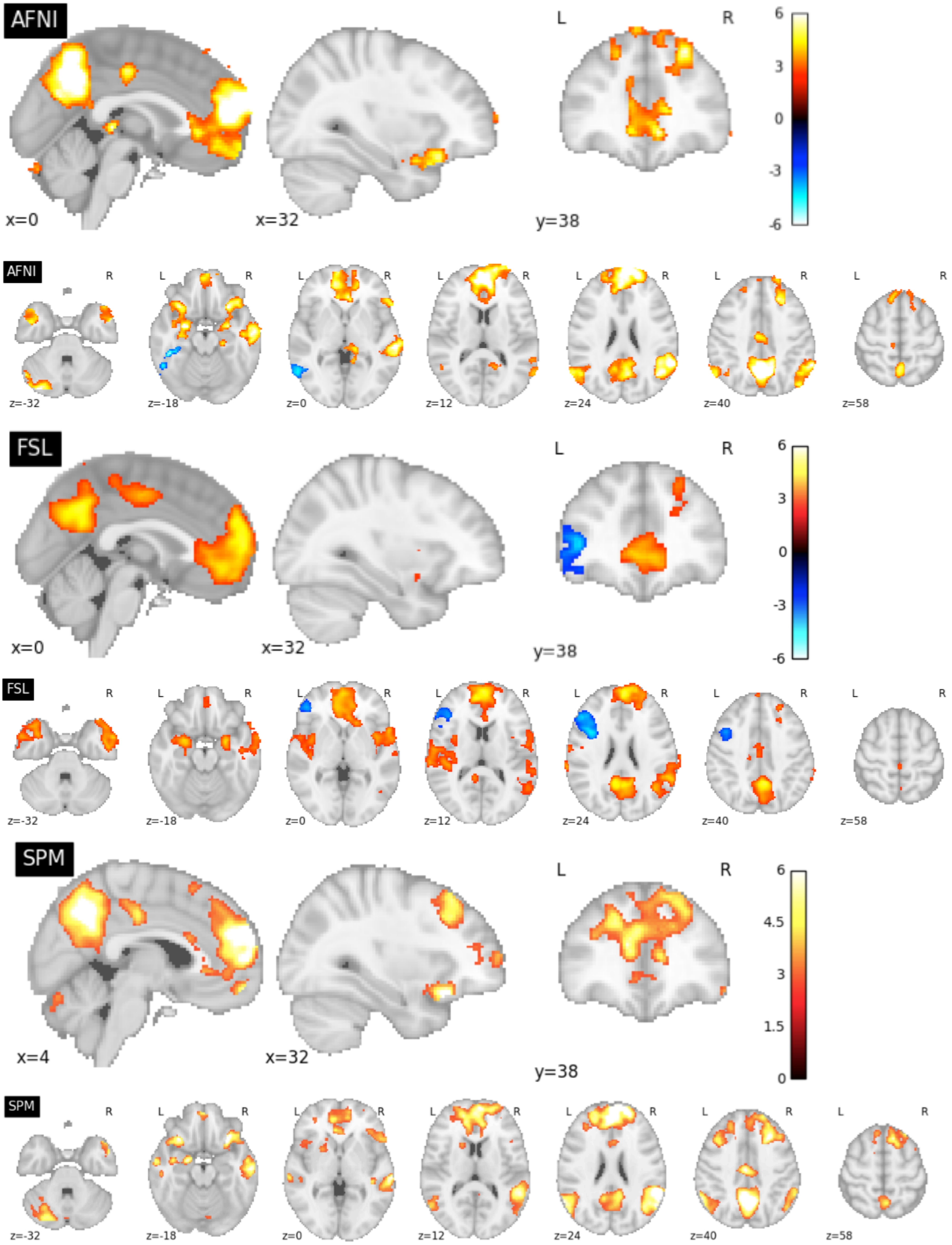

Figure S5. ds000109 Inter-Software Comparison, 5% FWE Clusterwise Permutation Inference

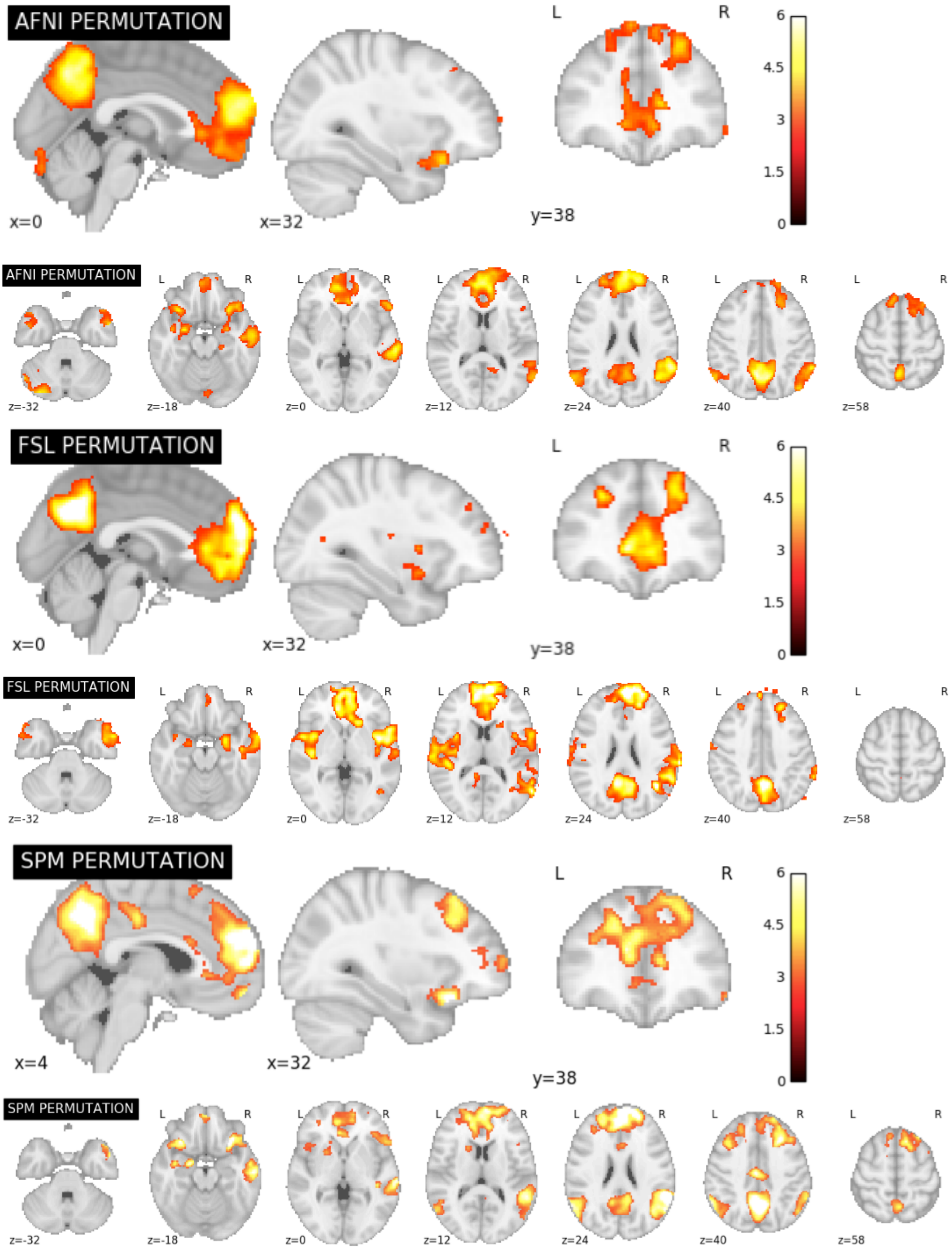

Figure S6. ds000120 Inter-Software Comparison, 5% FWE Clusterwise Inference

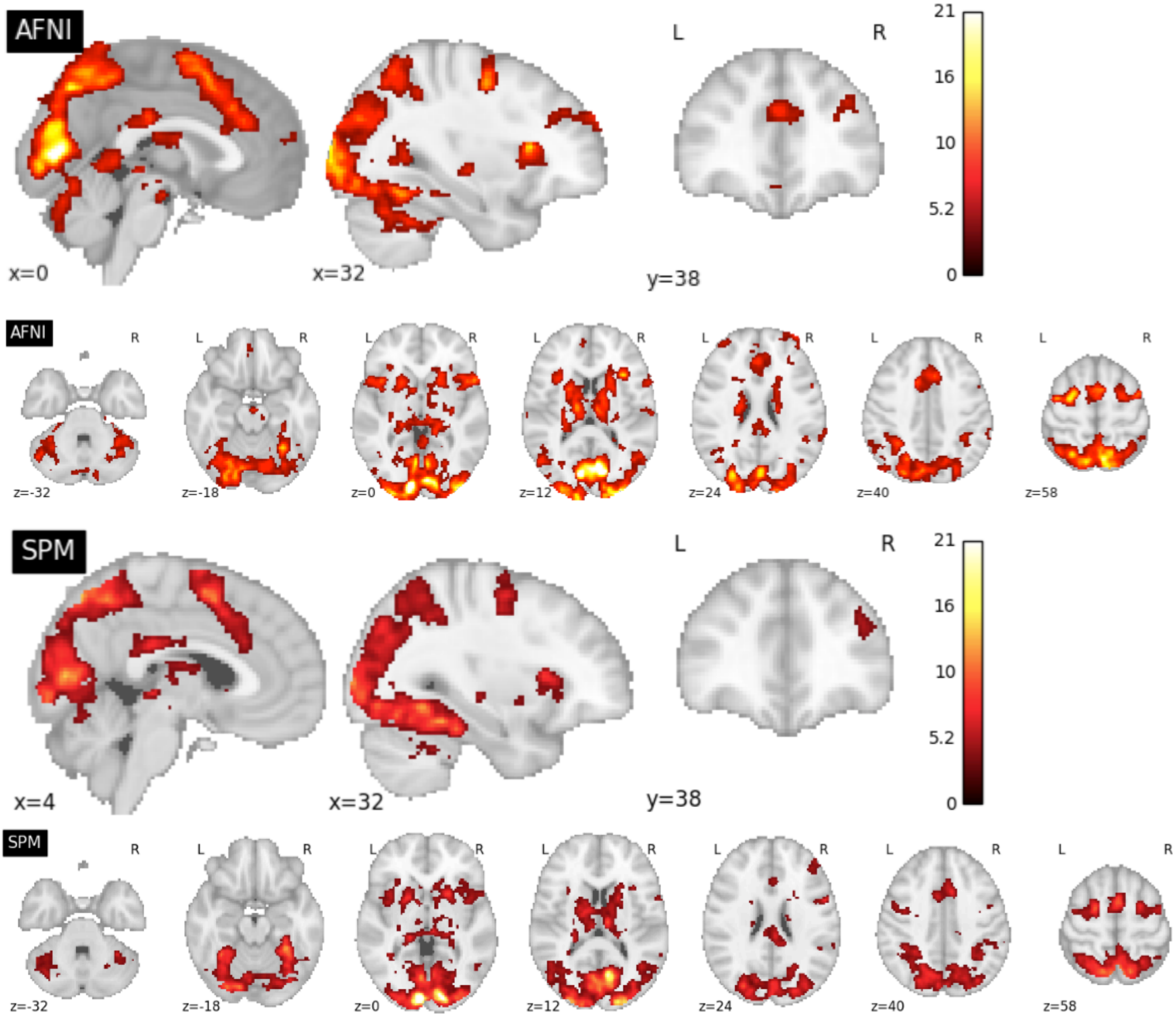

Figure S7. ds000001 Inter-Software Comparison, T-Statistic Maps

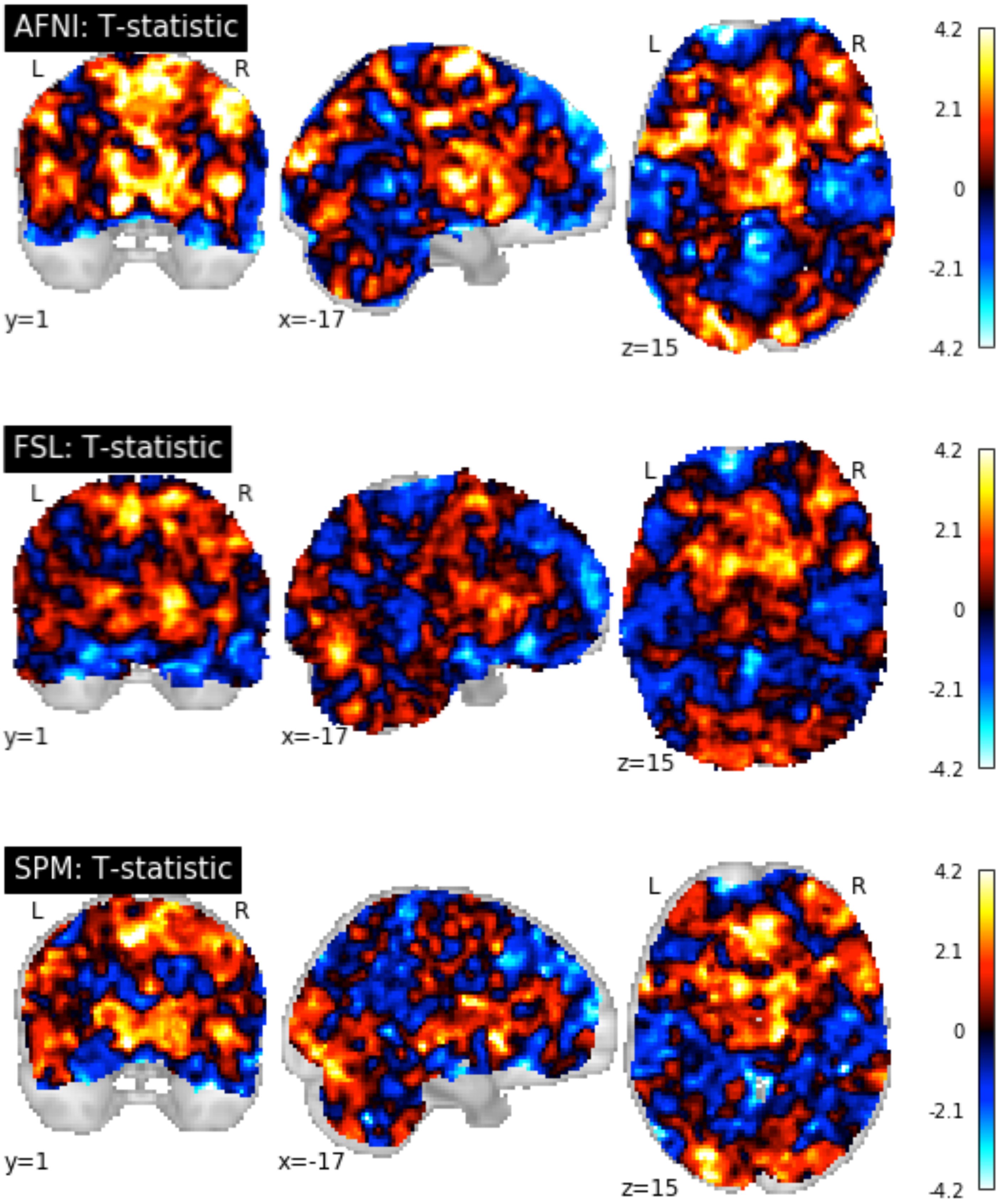

Figure S8. ds000001 Inter-Software Comparison, T-Statistic Maps from Permutation

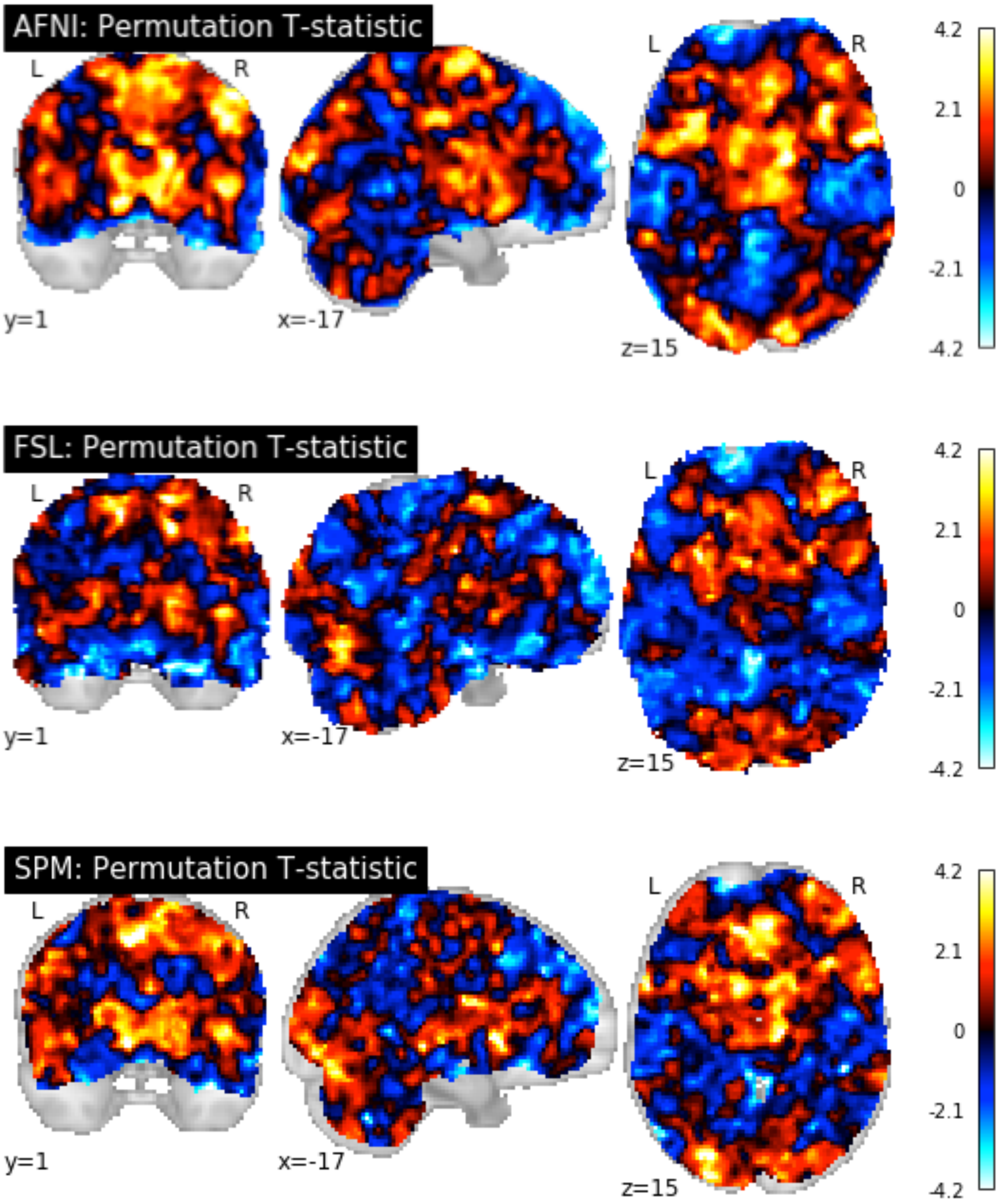

Figure S9. ds000109 Inter-Software Comparison, T-Statistic Maps

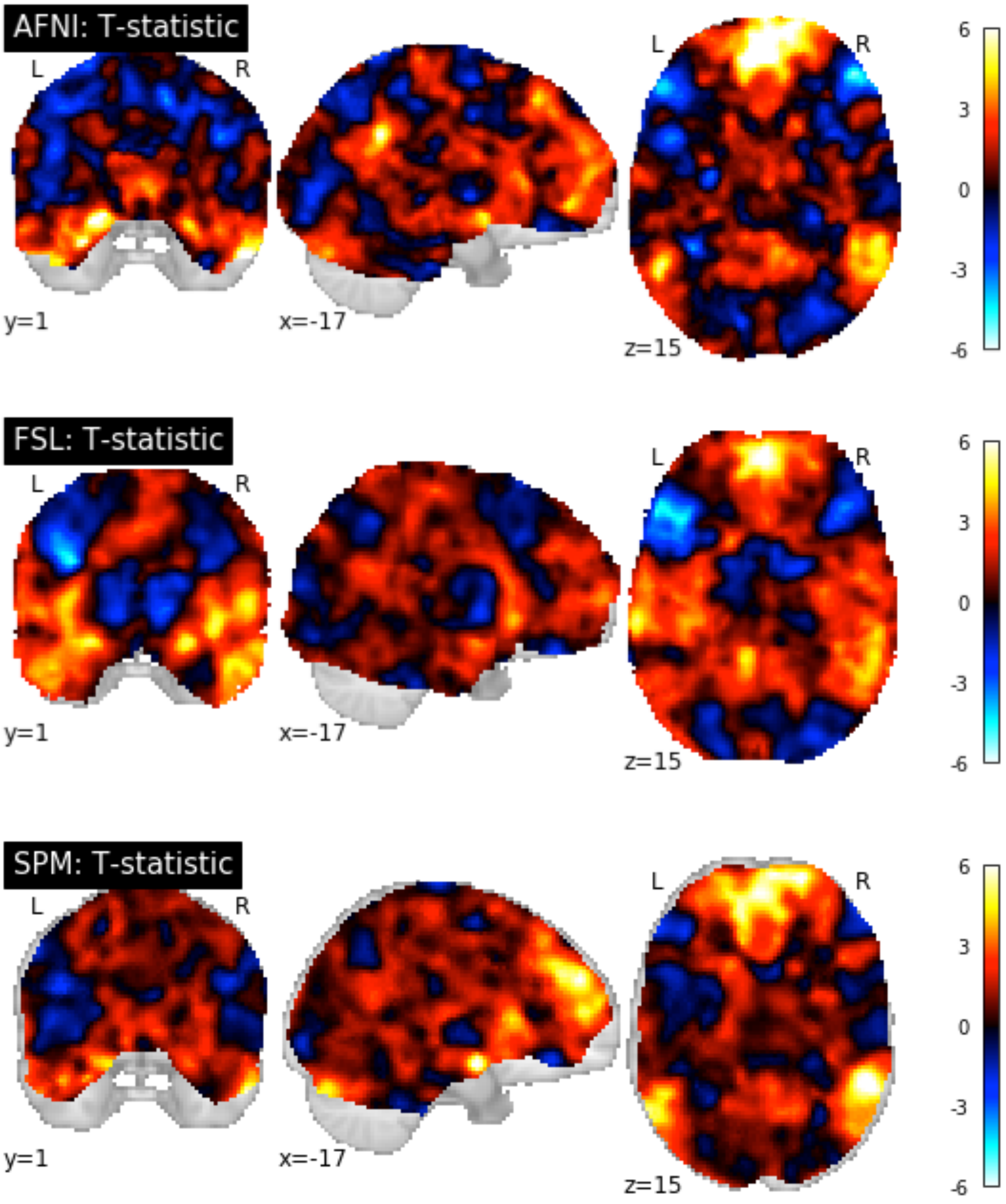

Figure S10. ds000109 Inter-Software Comparison, T-Statistic Maps from Permutation

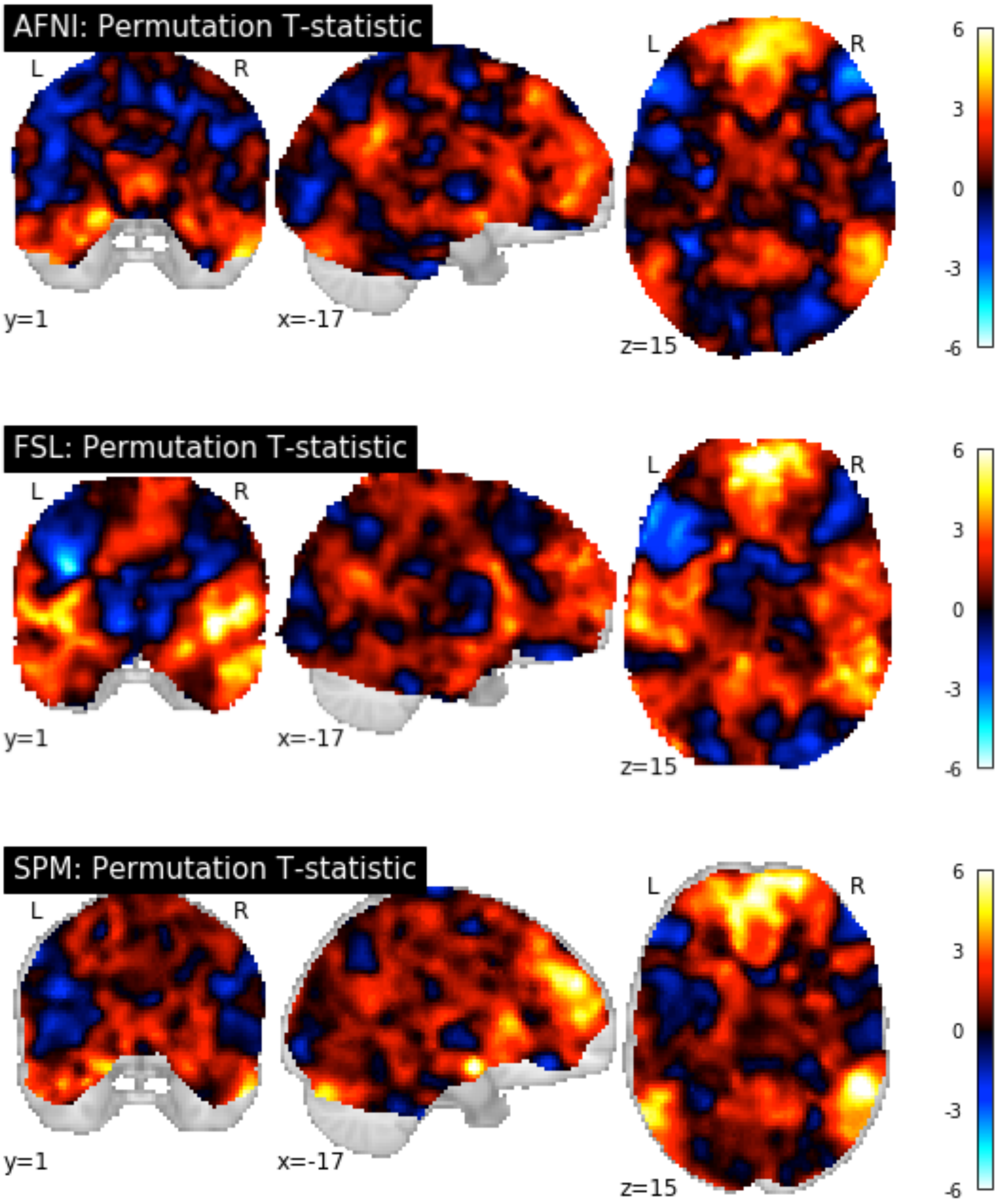

Figure S11. ds120 Inter-Software Comparison, F-Statistic Maps

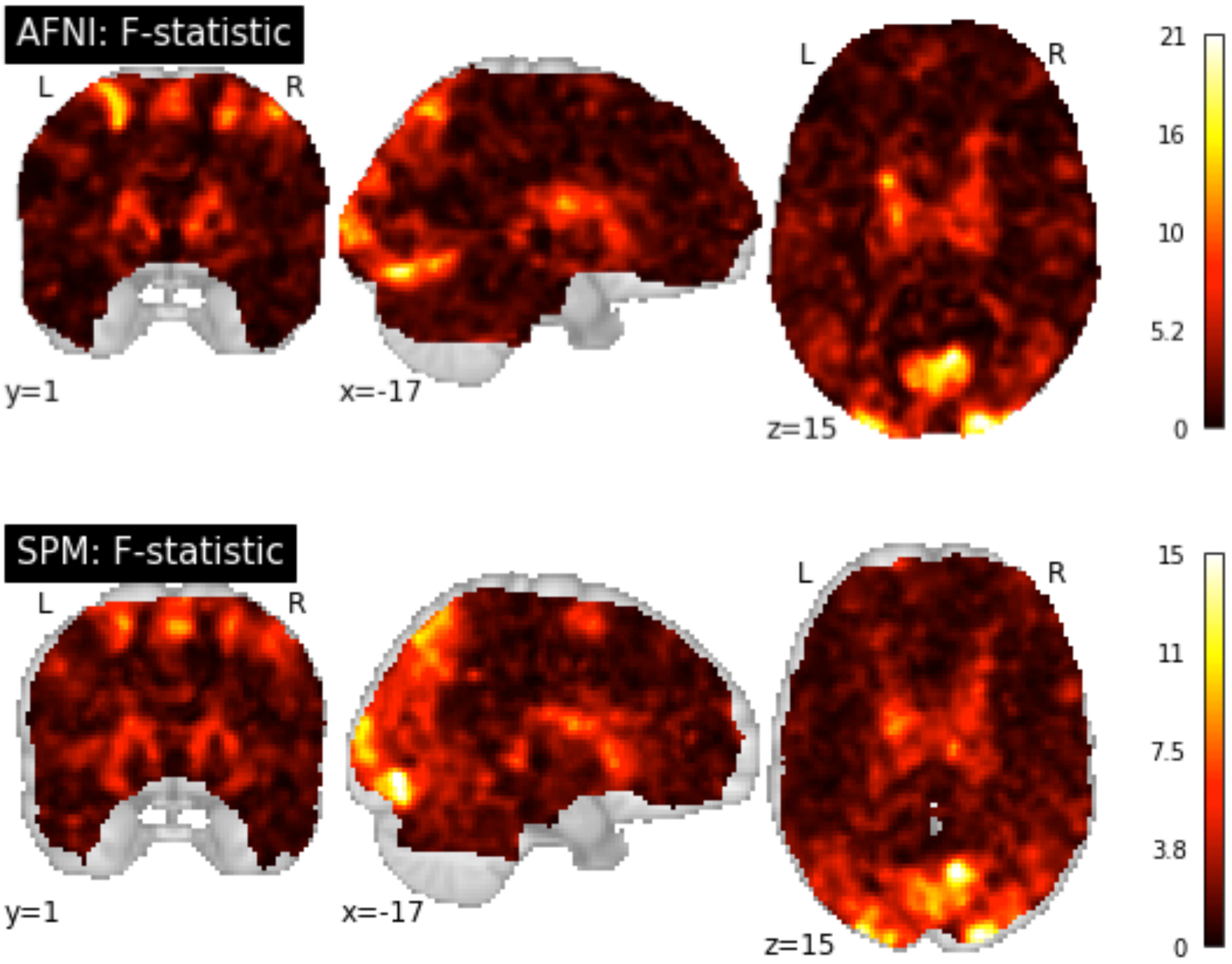

Figure S12. ds000120 Inter-Software Comparison, Euler Characteristic and Cluster Count Curves for F-Statistic Maps

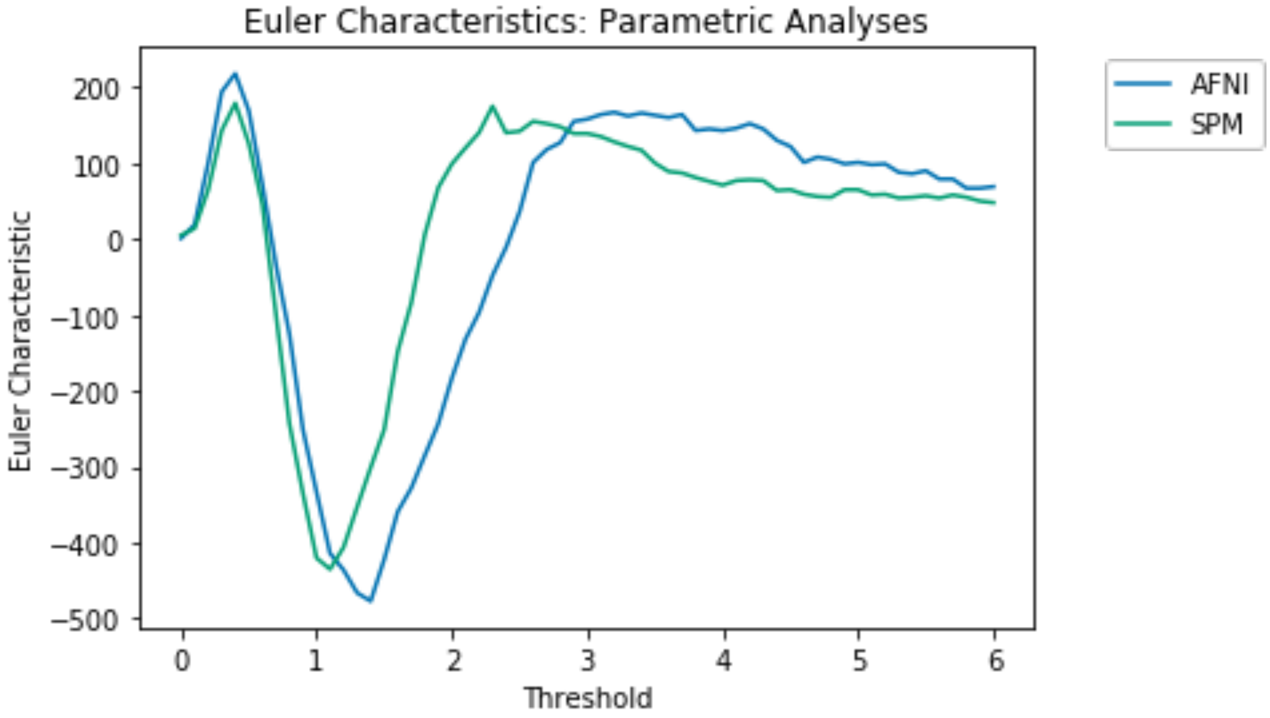

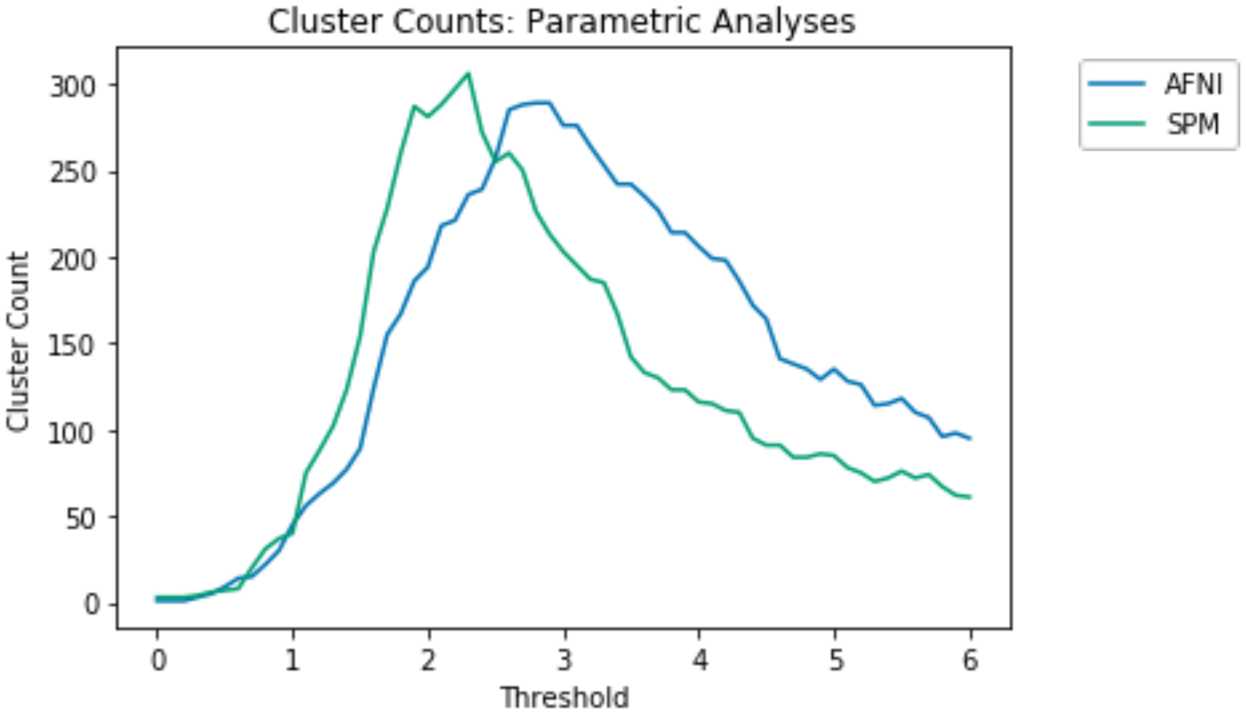

Figure S13. Bland-Altman Percent BOLD Comparisons

Bland-Altman Plots: Percent BOLD Comparisons

ds000001	ds000109

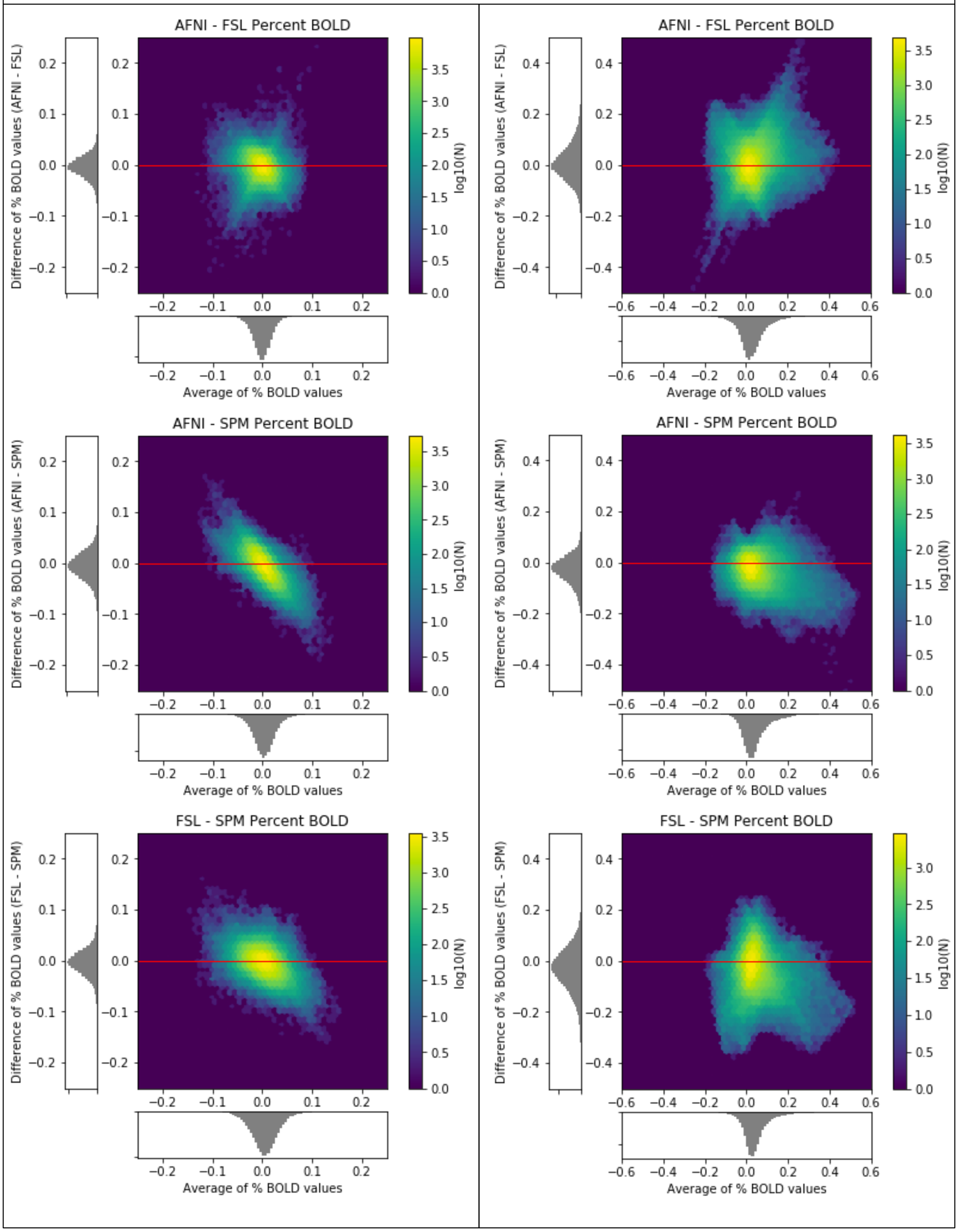

Figure S14. ds000120 R^2 Comparisons

Bland-Altman Plots: R^2 Comparisons
ds000120

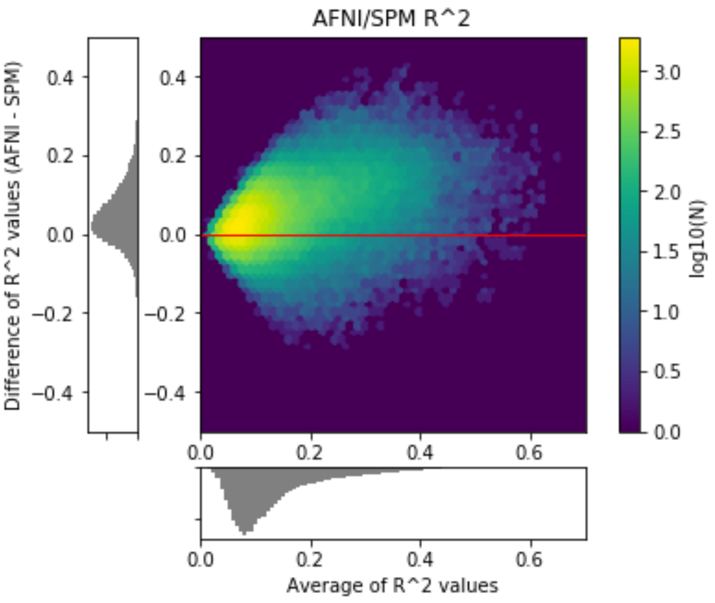

Supplement: Supplementary file 2 — Figure S1 Registration QC: Mean and standard deviation of anatomical and mean functional images Figure S2. ds000001 Inter‐Software Comparison, 5% FWE Clusterwise Inference Figure S3. ds000001 Inter‐Software Comparison, 5% FWE Clusterwise Permutation Inference Figure S4. ds000109 Inter‐Software Comparison, 5% FWE Clusterwise Inference Figure S5. ds000109 Inter‐Software Comparison, 5% FWE Clusterwise Permutation Inference Figure S6. ds000120 Inter‐Software Comparison, 5% FWE Clusterwise Inference Figure S7. ds000001 Inter‐Software Comparison, T‐Statistic Maps Figure S8. ds000001 Inter‐Software Comparison, T‐Statistic Maps from Permutation Figure S9. ds000109 Inter‐Software Comparison, T‐Statistic Maps Figure S10. ds000109 Inter‐Software Comparison, T‐Statistic Maps from Permutation Figure S11. ds120 Inter‐Software Comparison, F‐Statistic Maps Figure S12. ds000120 Inter‐Software Comparison, Euler Characteristic and Cluster Count Curves for F‐Statistic Maps Figure S13. Bland–Altman Percent BOLD Comparisons Figure S14. ds000120 R2 Comparisons [file HBM-40-3362-s002.pdf]
