# Supplementary material for: Erratum: Exploring the impact of analysis software on task fMRI results
Source: Hum Brain Mapp. 2020 Dec 14;42(5):1564–78. doi: 10.1002/hbm.25302 (PMC7927288; doi:10.1002/hbm.25302)
Supplement: Supplementary file 1 — Appendix S1. Supporting Figures [file HBM-42-1564-s001.pdf]

<u>Supplementary Figures</u>

Figure S1. Registration QC: Mean and standard deviation of anatomical and mean functional images

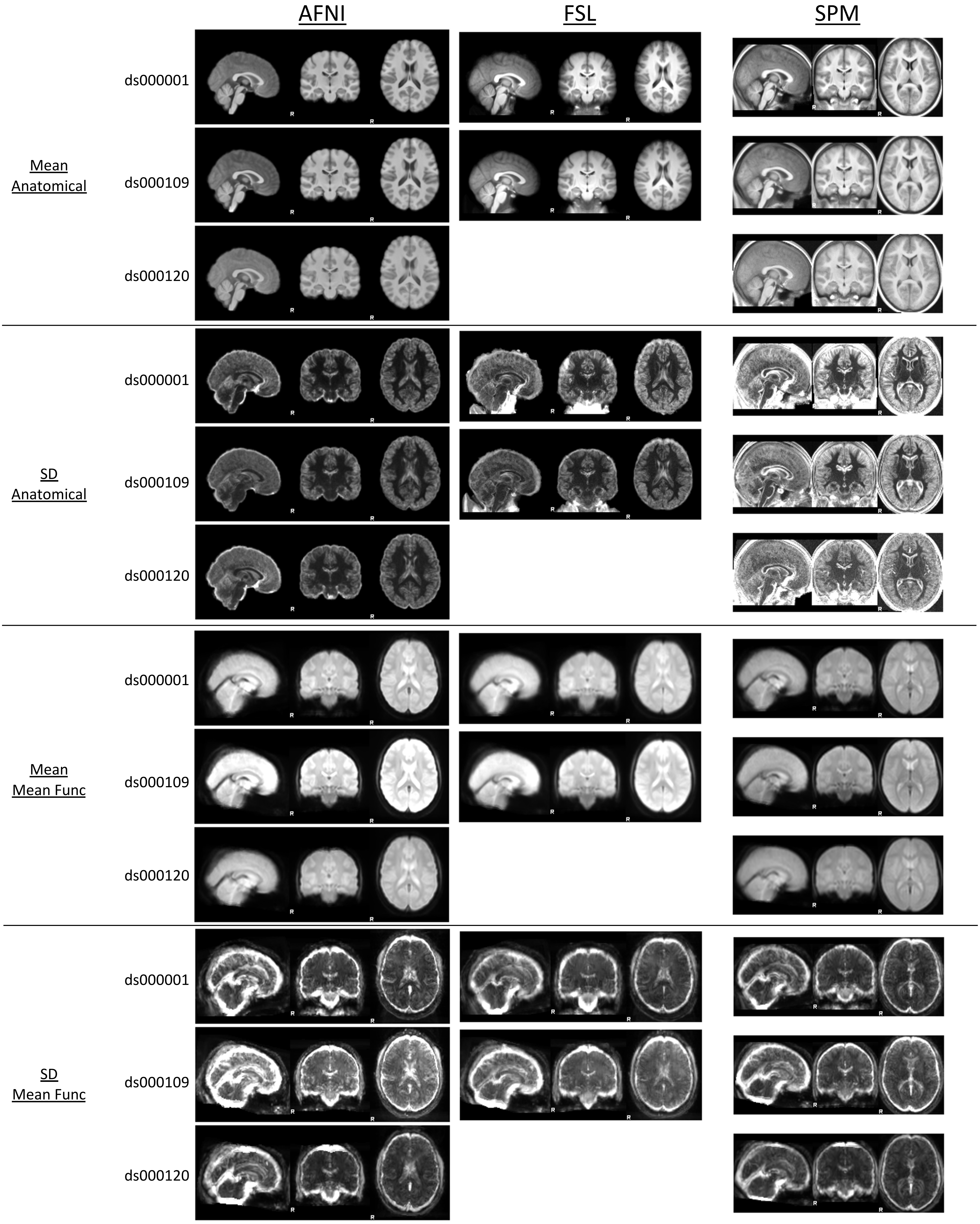

Figure S2. ds000001 Inter-Software Comparison, 5% FWE Clusterwise Inference

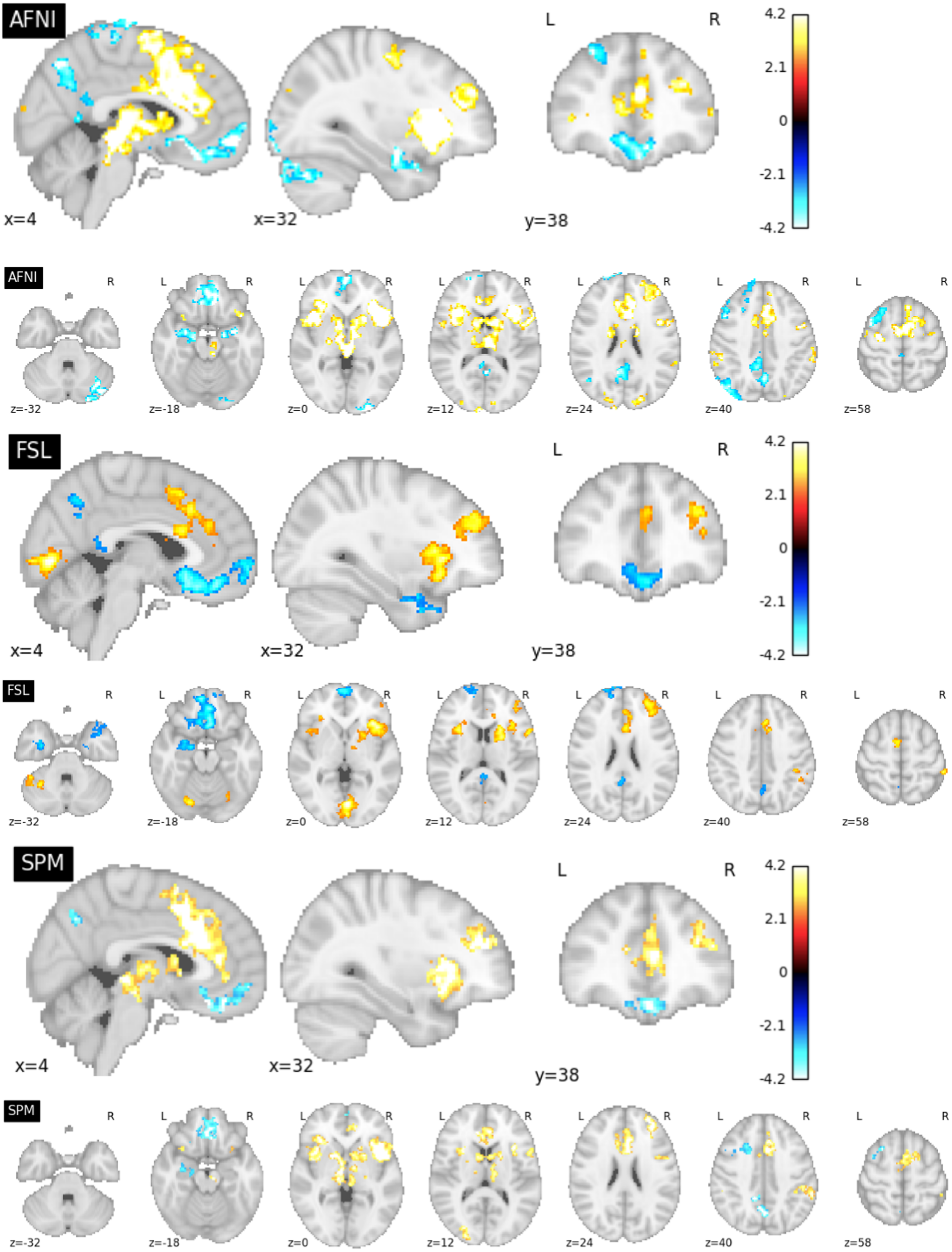

Figure S3. ds000001 Inter-Software Comparison, 5% FWE Clusterwise Permutation Inference

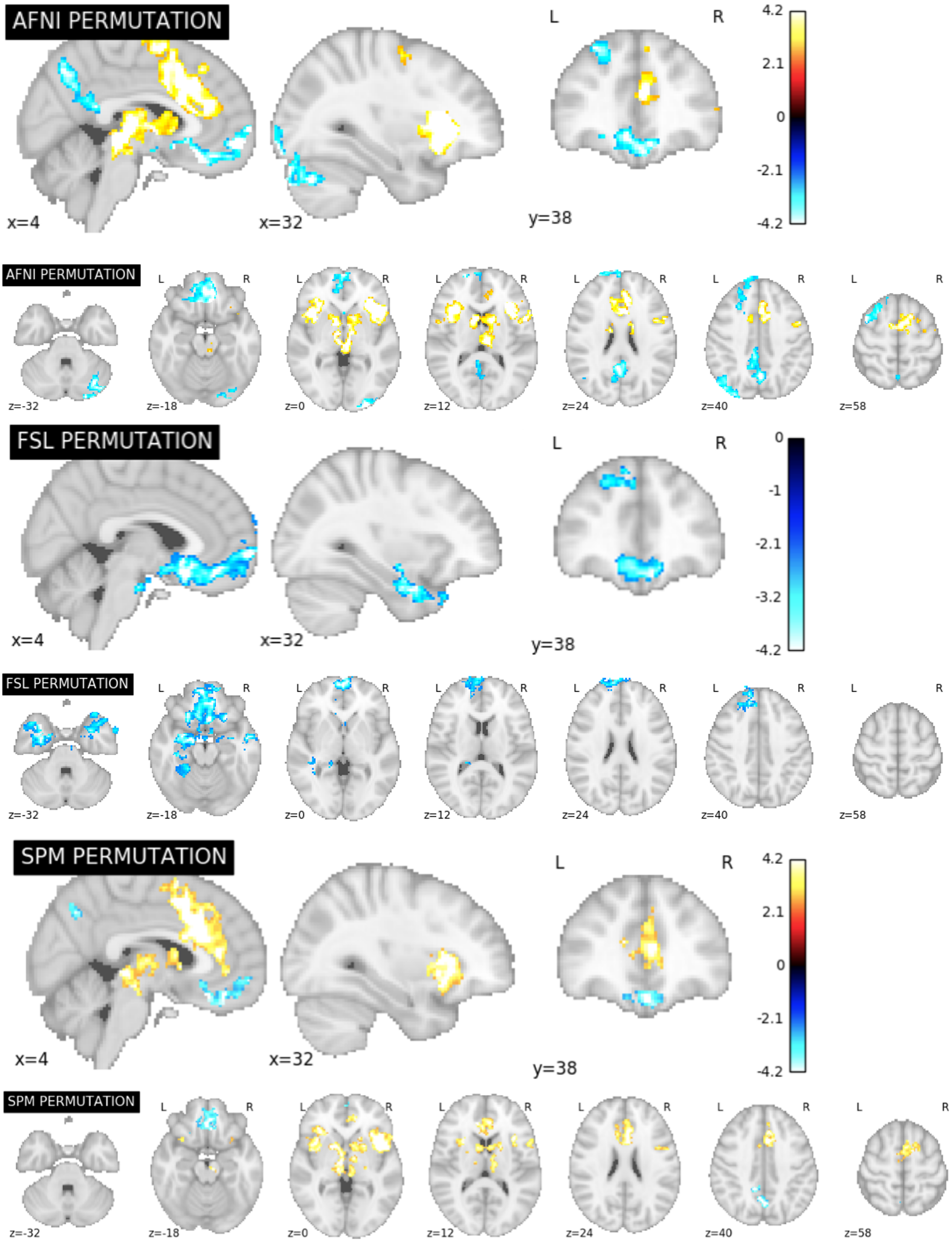

Figure S4. ds000109 Inter-Software Comparison, 5% FWE Clusterwise Inference

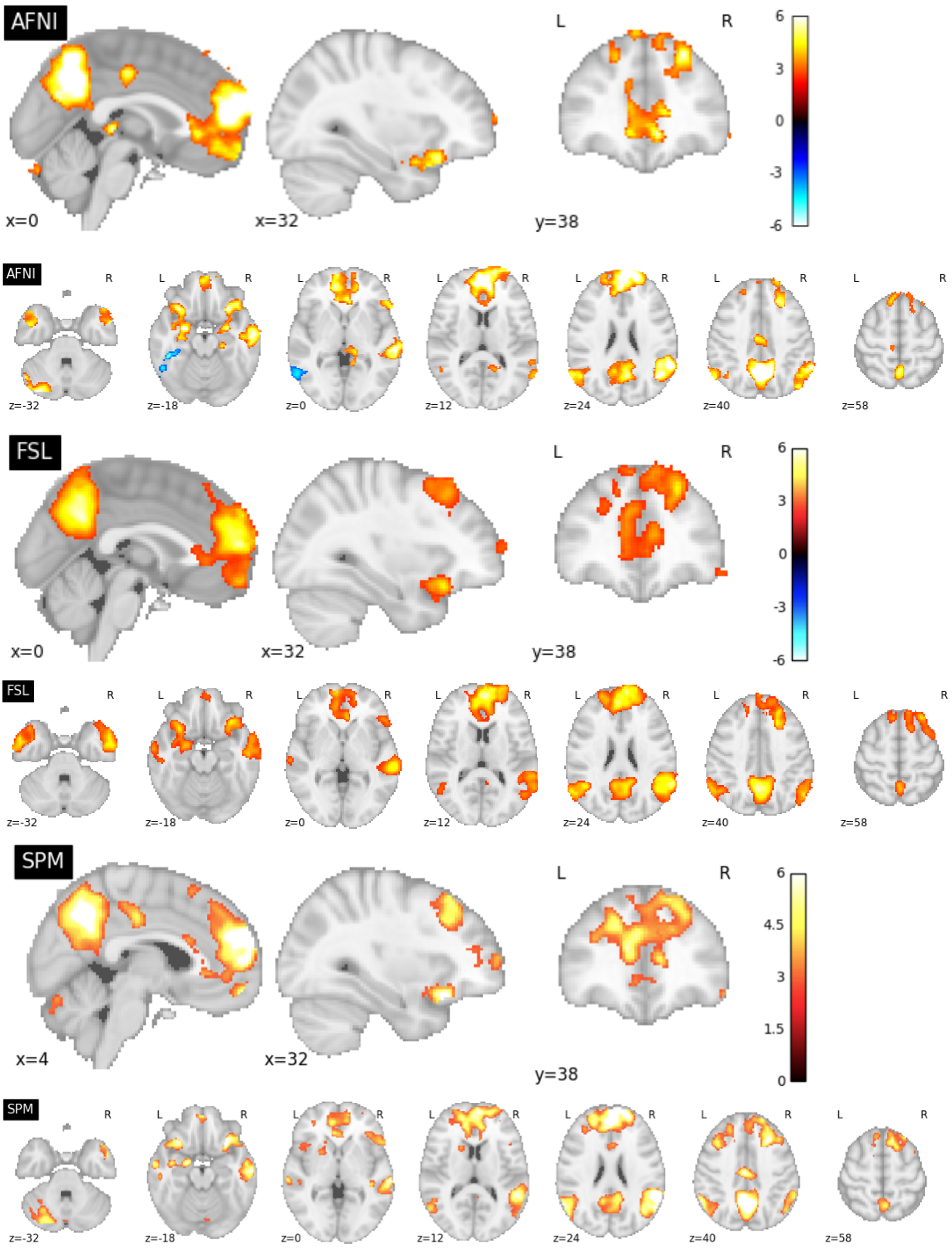

Figure S5. ds000109 Inter-Software Comparison, 5% FWE Clusterwise Permutation Inference

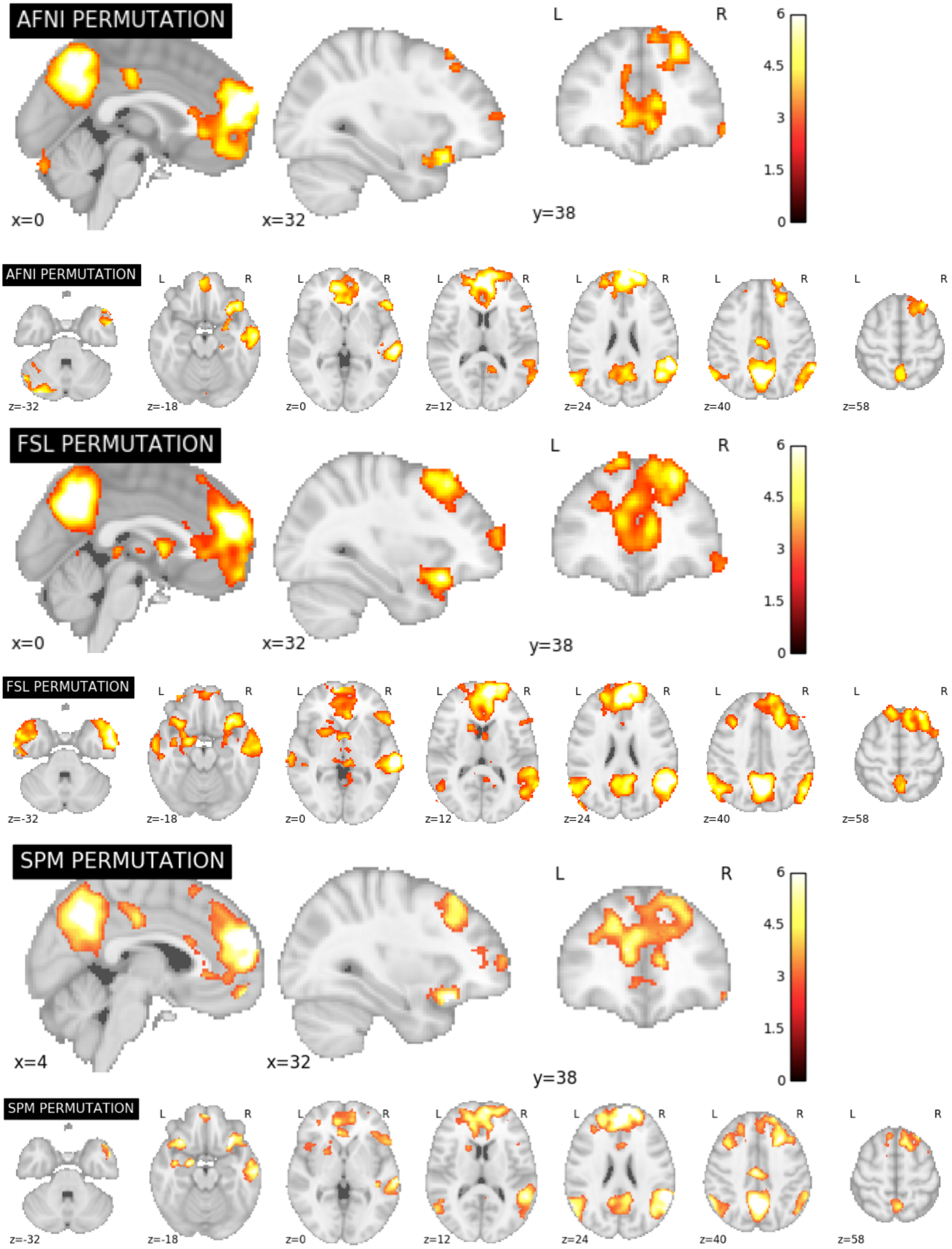

Figure S6. ds000120 Inter-Software Comparison, 5% FWE Clusterwise Inference

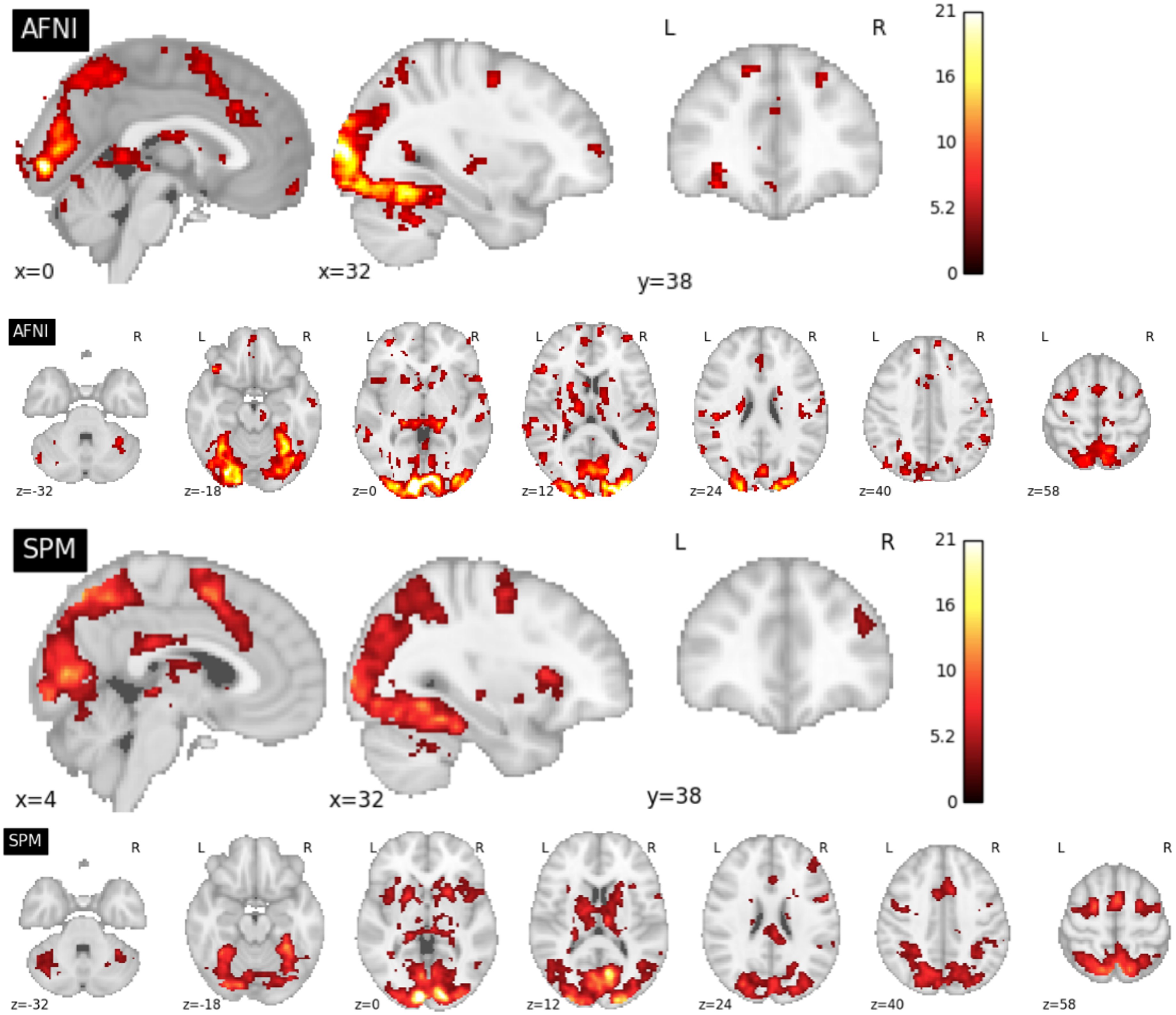

Figure S7. ds000001 Inter-Software Comparison, T-Statistic Maps

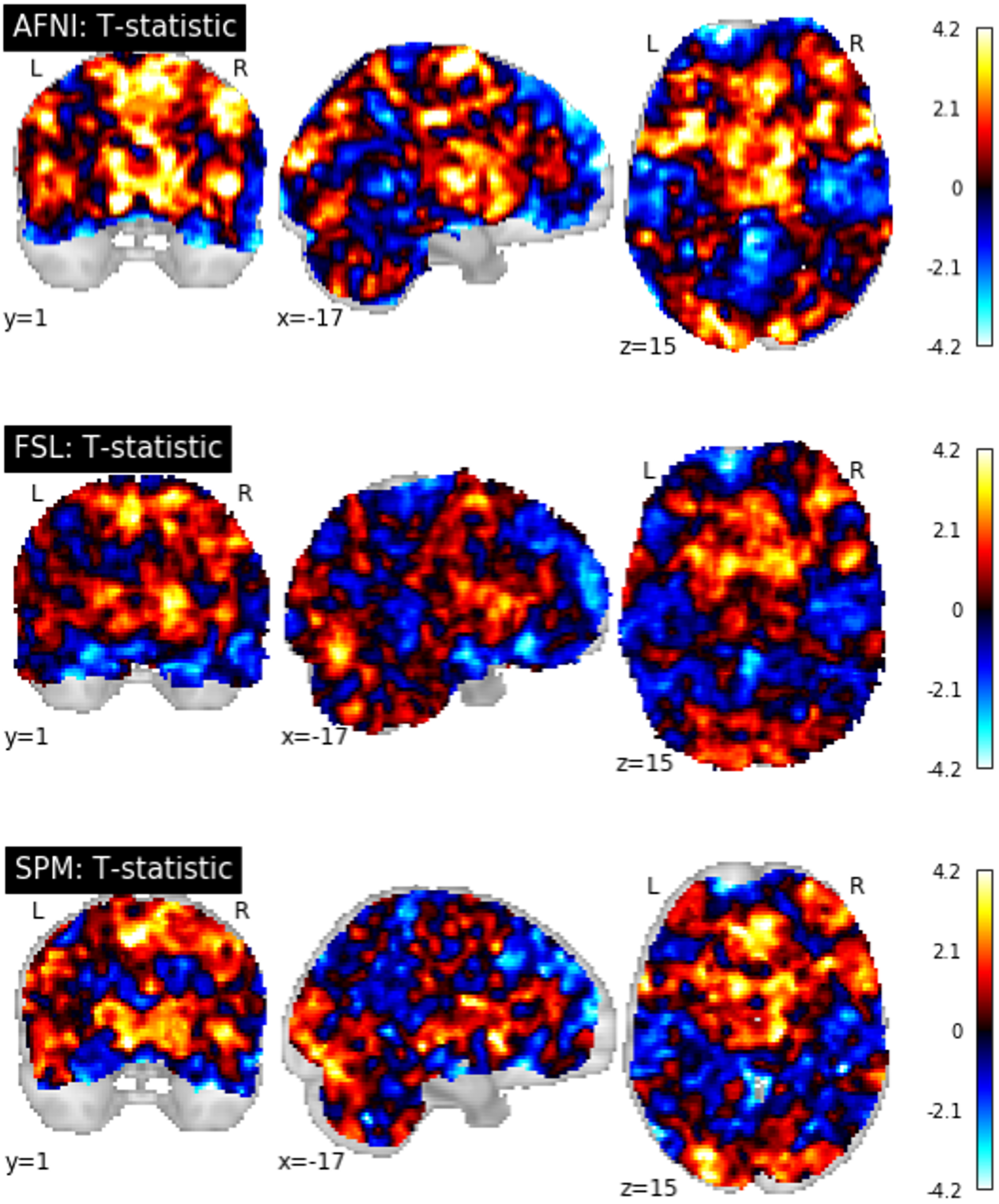

Figure S8. ds000001 Inter-Software Comparison, T-Statistic Maps from Permutation

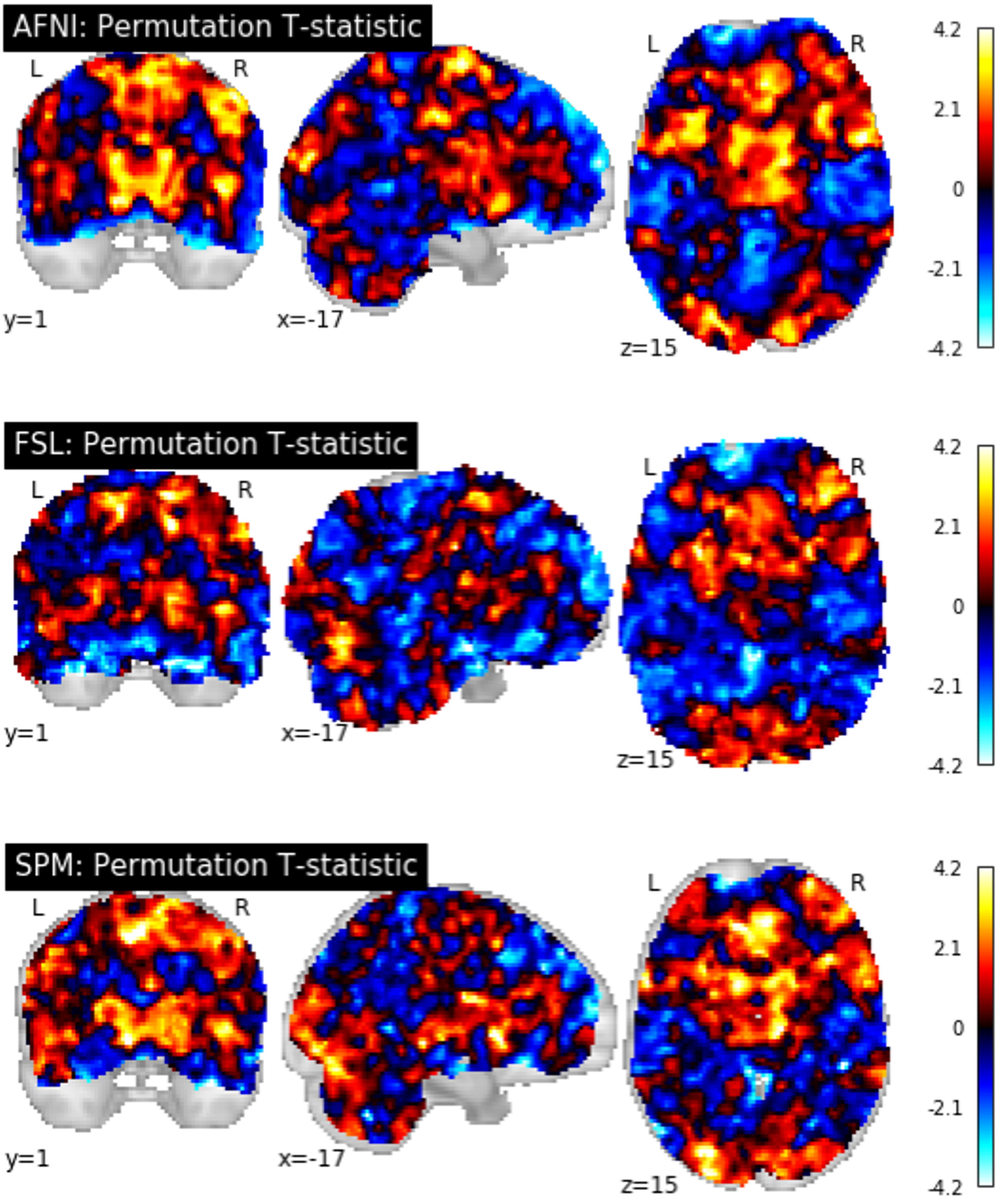

Figure S9. ds000109 Inter-Software Comparison, T-Statistic Maps

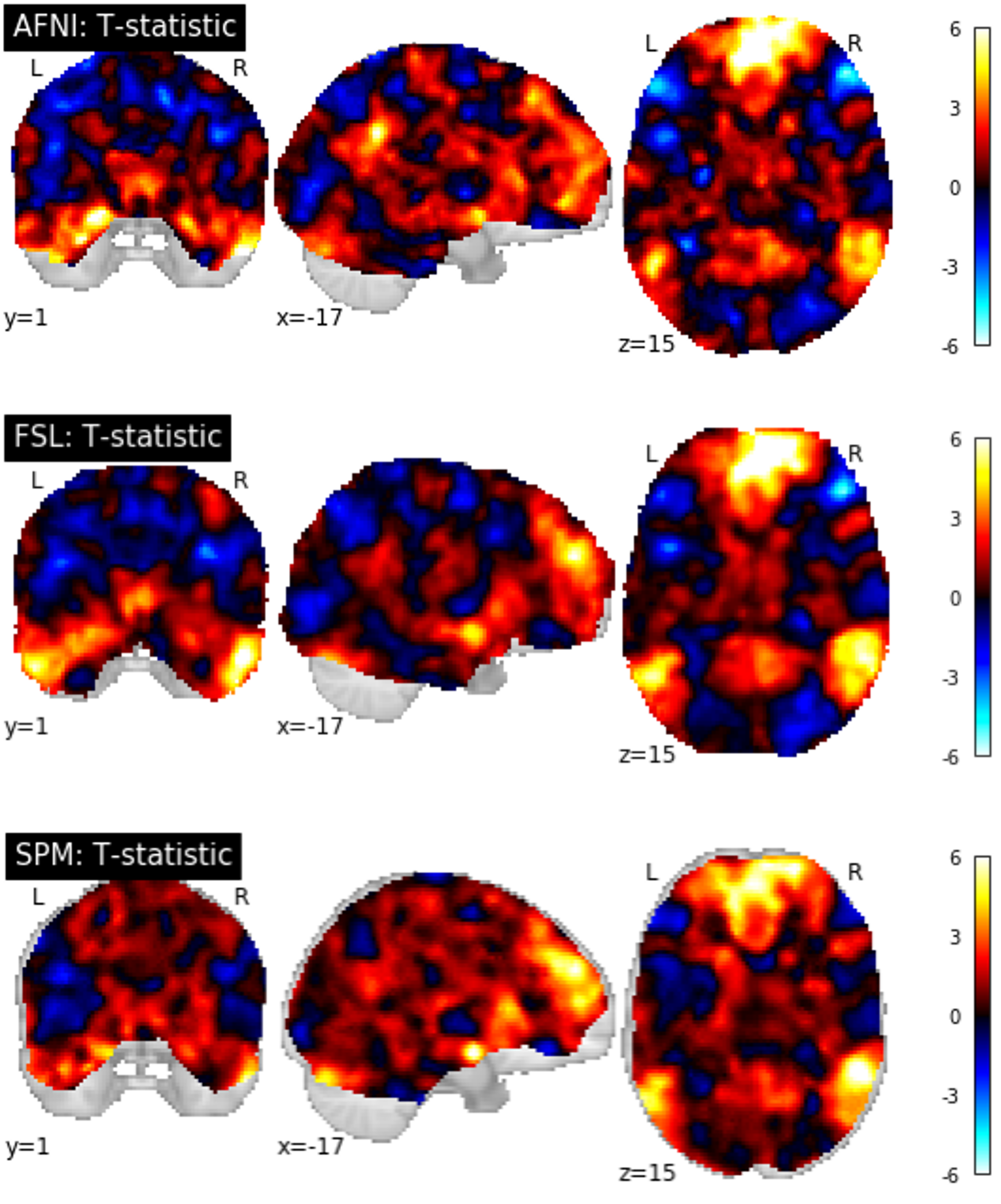

Figure S10. ds000109 Inter-Software Comparison, T-Statistic Maps from Permutation

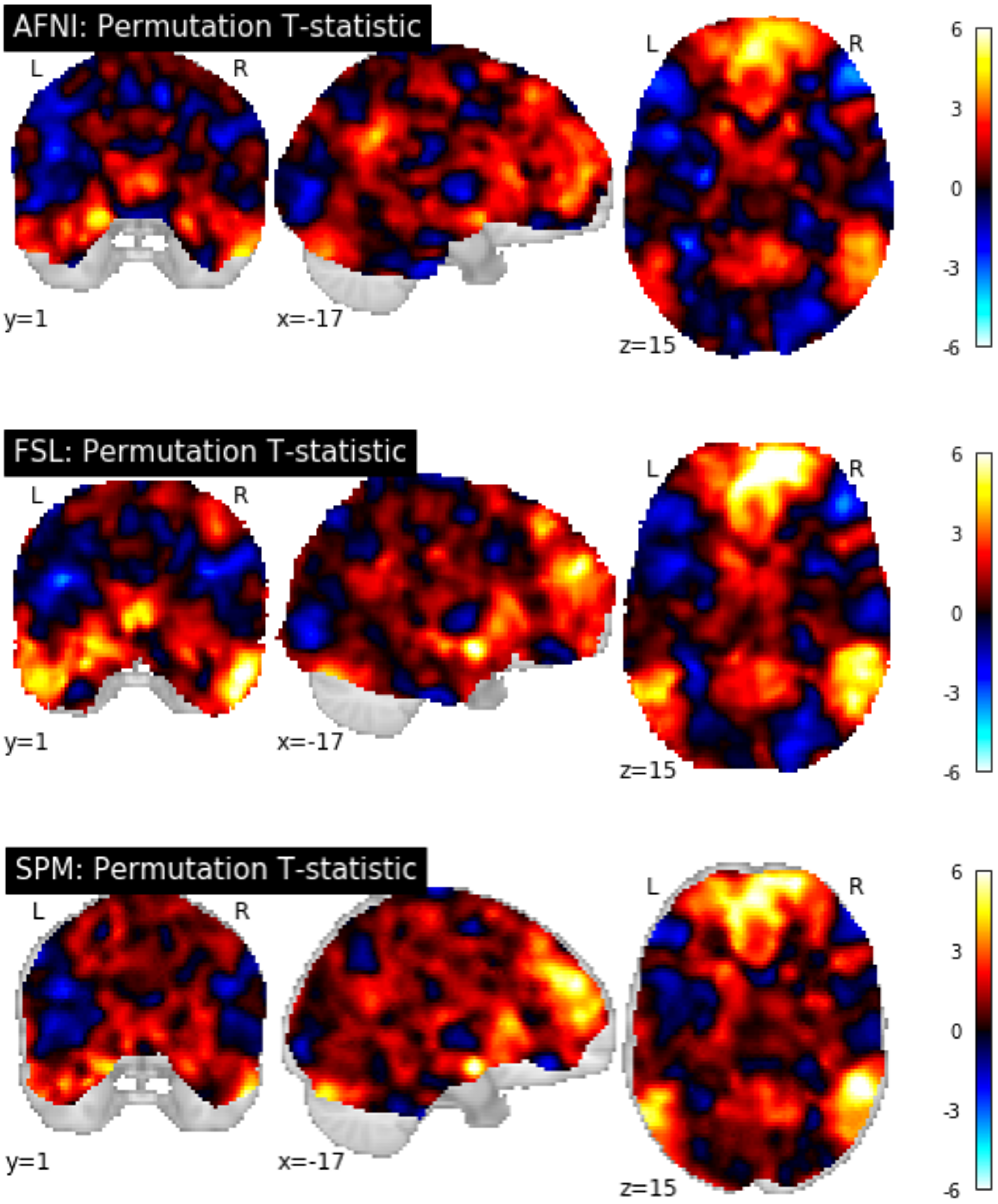

Figure S11. ds120 Inter-Software Comparison, F-Statistic Maps

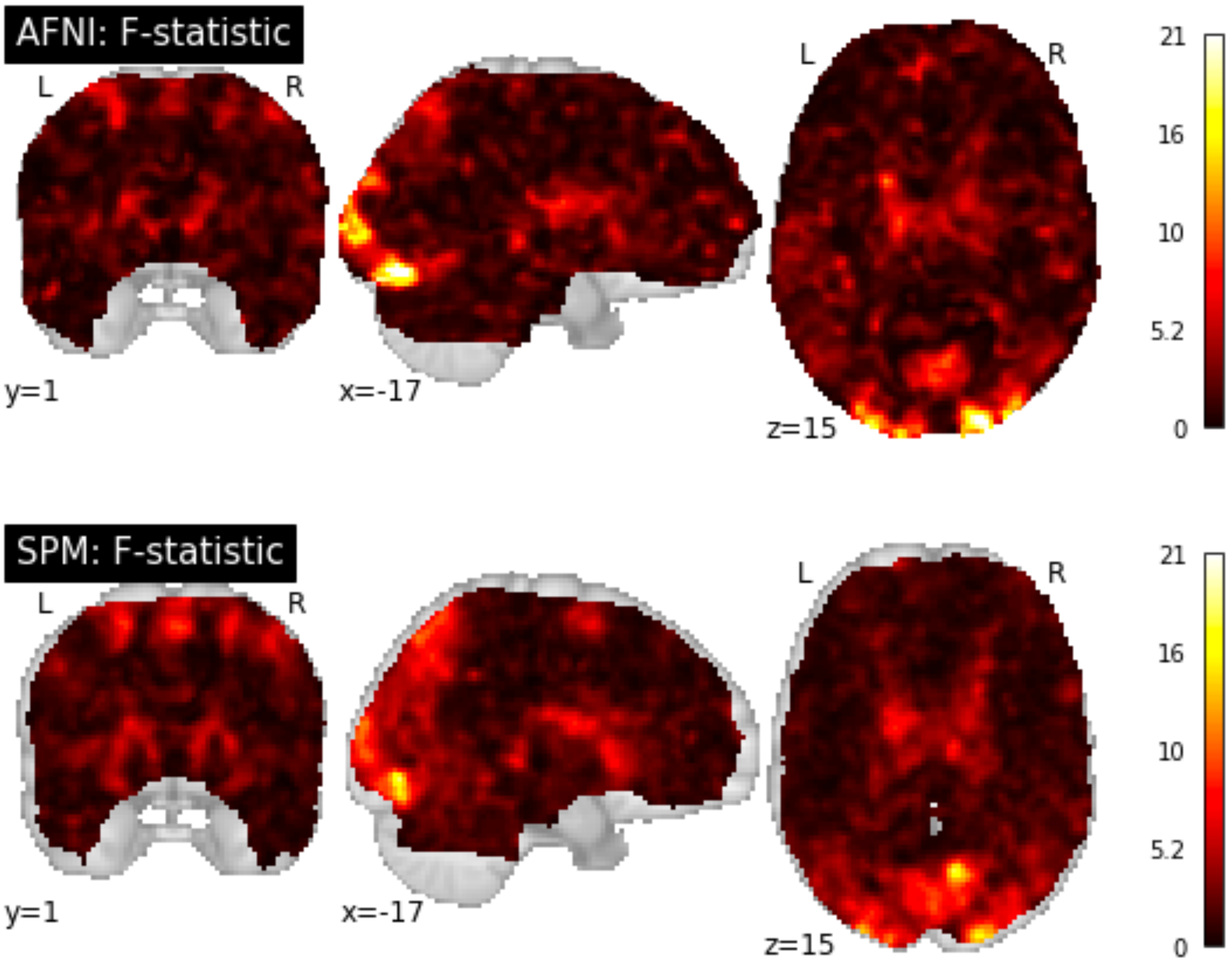

Figure S12. ds000120 Inter-Software Comparison, Euler Characteristic and Cluster Count Curves for F-Statistic Maps

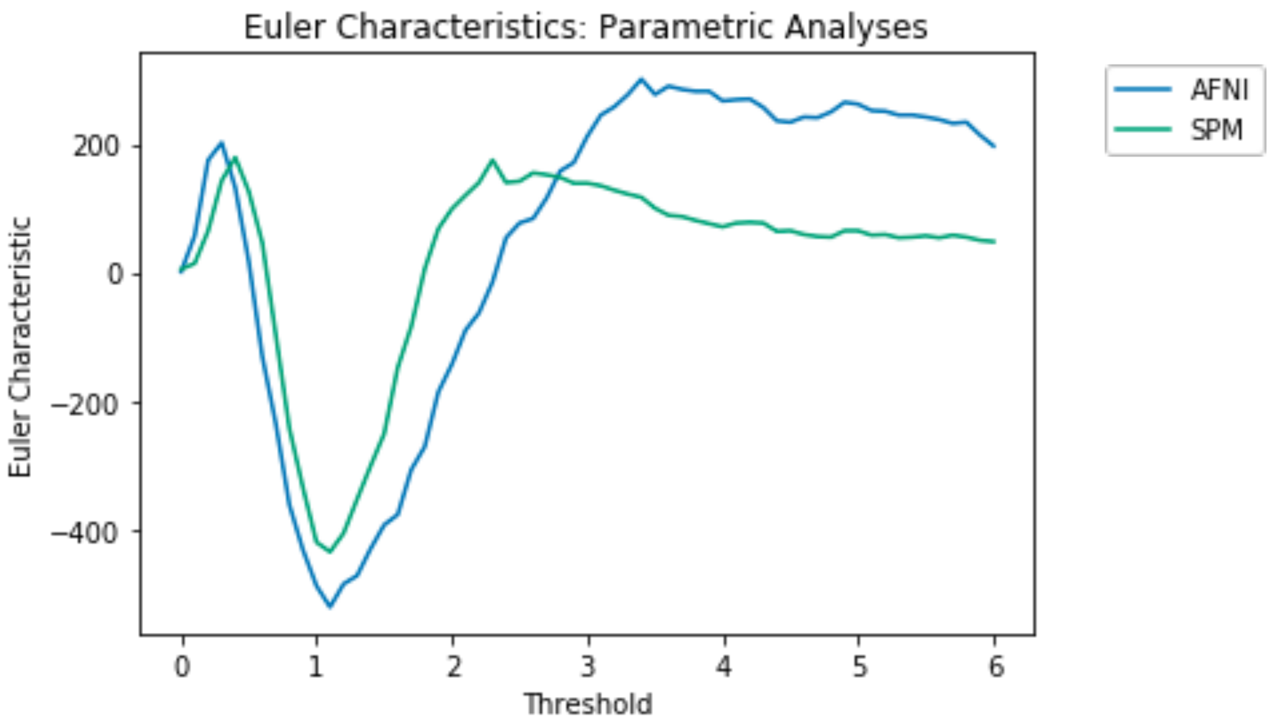

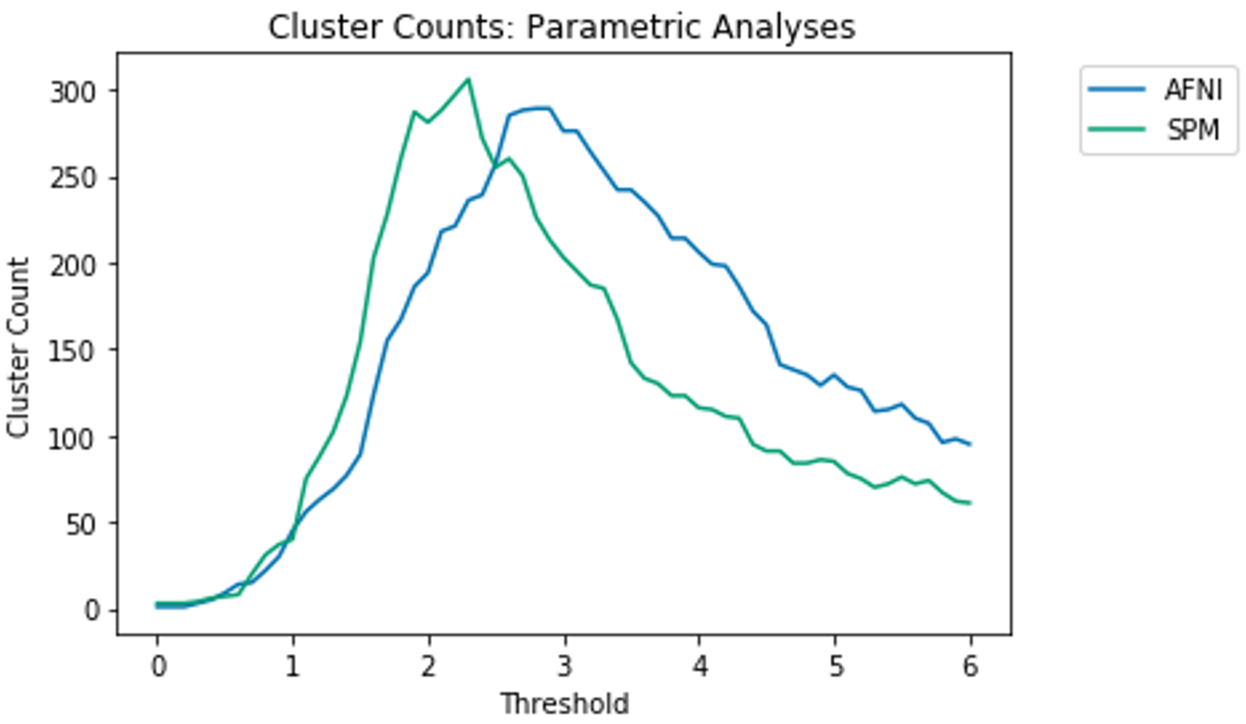

Figure S13. Bland-Altman Percent BOLD Comparisons

Bland-Altman Plots: Percent BOLD Comparisons

ds000001	ds000109

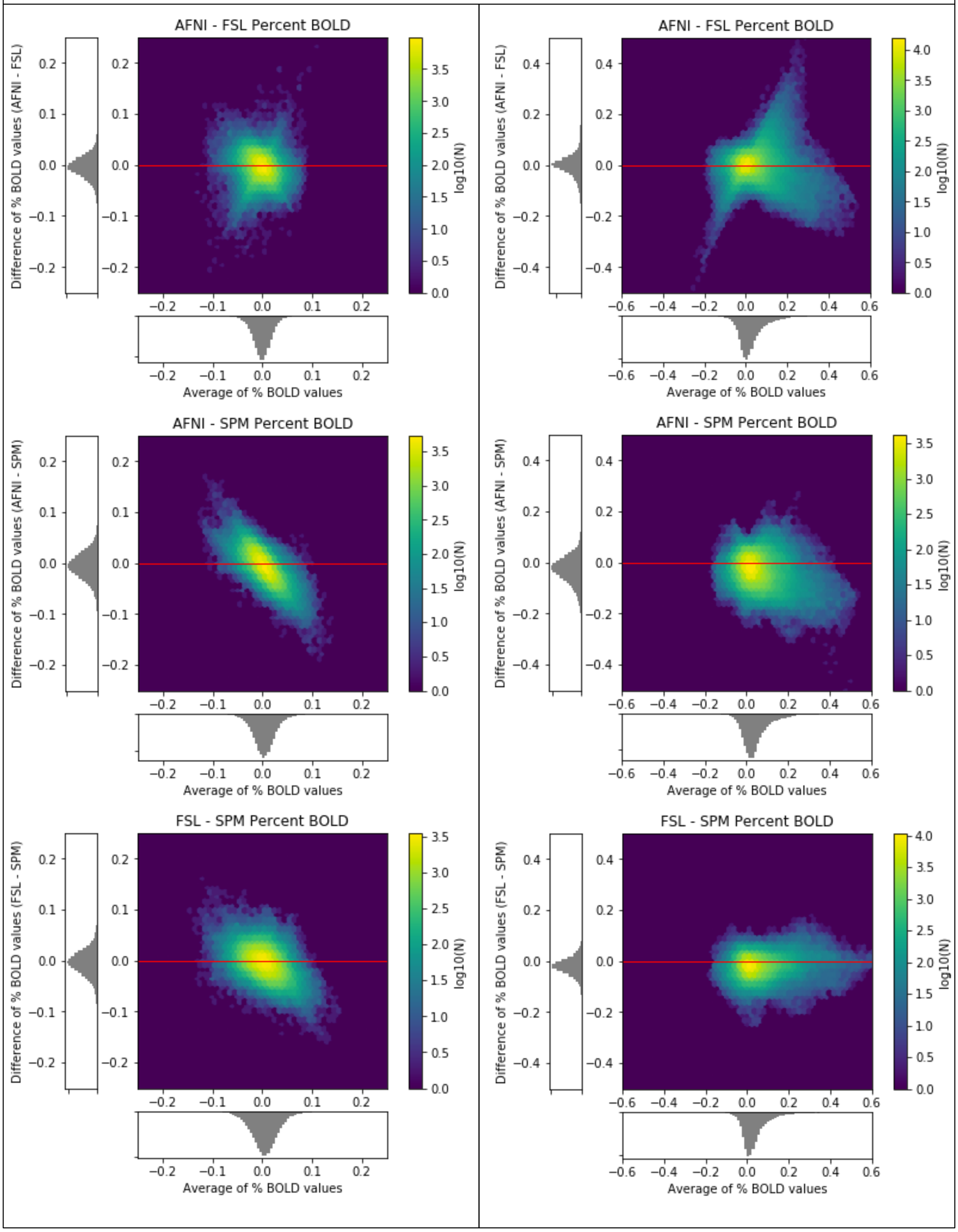

Figure S14. ds000120 R^2 Comparisons

Bland-Altman Plots: R^2 Comparisons
ds000120
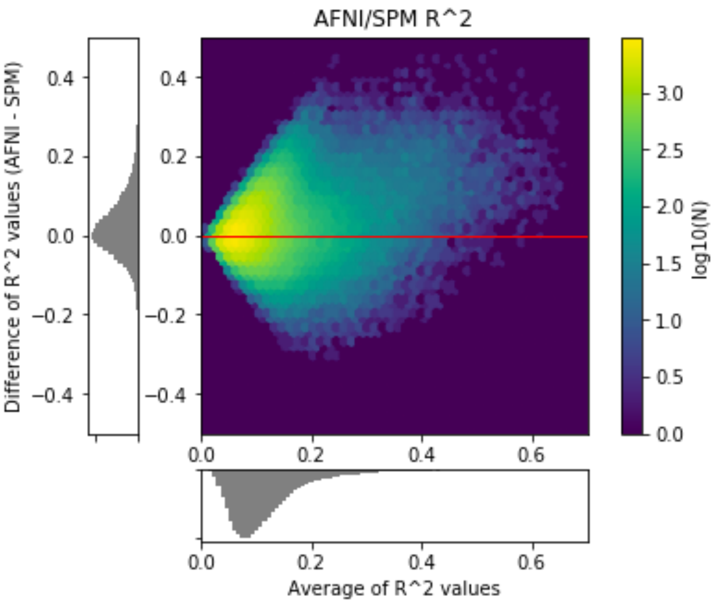
